# Tas2R signaling enhances mouse neutrophil migration *via* a ROCK-dependent pathway

**DOI:** 10.3389/fimmu.2022.973880

**Published:** 2022-08-18

**Authors:** Daichi Kobayashi, Tomoya Watarai, Madoka Ozawa, Yasuhiro Kanda, Fumihiro Saika, Norikazu Kiguchi, Arata Takeuchi, Masahito Ikawa, Shinsuke Matsuzaki, Tomoya Katakai

**Affiliations:** ^1^ Department of Immunology, Niigata University Graduate School of Medical and Dental Sciences, Niigata, Japan; ^2^ Department of Pharmacology, Wakayama Medical University, Wakayama, Japan; ^3^ Department of Physiological Sciences, School of Pharmaceutical Sciences, Wakayama Medical University, Wakayama, Japan; ^4^ Department of Immunology, Tokyo Medical University, Tokyo, Japan; ^5^ Department of Experimental Genome Research, Research Institute for Microbial Diseases, Osaka University, Suita, Japan; ^6^ Department of Radiological Sciences, Faculty of Medical Science Technology, Morinomiya University of Medical Sciences, Osaka, Japan; ^7^ Department of Child Development and Molecular Brain Science, United Graduate School of Child Development, Suita, Japan

**Keywords:** Tas2R, cell migration, bitter taste substances, ROCK, neutrophils

## Abstract

Type-2 bitter taste receptors (Tas2Rs) are a large family of G protein-coupled receptors that are expressed in the oral cavity and serve to detect substances with bitter tastes in foods and medicines. Recent evidence suggests that Tas2Rs are also expressed extraorally, including in immune cells. However, the role of Tas2Rs in immune cells remains controversial. Here, we demonstrate that Tas2R126, Tas2R135, and Tas2R143 are expressed in mouse neutrophils, but not in other immune cells such as macrophages or T and B lymphocytes. Treatment of bone marrow-derived neutrophils from wild-type mice with the Tas2R126/143 agonists arbutin and d-salicin led to enhanced C-X-C motif chemokine ligand 2 (CXCL2)-stimulated migration *in vitro*, but this response was not observed in neutrophils from *Tas2r126/135/143*-deficient mice. Enhancement of CXCL2-stimulated migration by Tas2R agonists was accompanied by increased phosphorylation of myosin light chain 2 (MLC2) and was blocked by pretreatment of neutrophils with inhibitors of Rho-associated coiled-coil-containing protein kinase (ROCK), but not by inhibitors of the small GTPase RhoA. Taken together, these results demonstrate that mouse neutrophils express functional Tas2R126/143 and suggest a role for Tas2R126/143–ROCK–MLC2-dependent signaling in the regulation of neutrophil migration.

## Introduction

Type-2 bitter taste receptors (TAS2Rs (human) or Tas2Rs (mouse)) are a large family of G protein-coupled receptors that are expressed in taste receptor cells in the oral cavity. The Tas2R family comprises approximately 30 members in humans and rodents (1, 2). In the oral cavity, activation of Tas2Rs (or TAS2Rs) induced by bitter taste substances transduces downstream molecules such as specific G proteins and TRPM5 (3, 4), and the bitter taste are recognized. Some of these substances are subsequently rejected from the oral cavity due to their bitter taste being potentially harmful to our health. Increasing evidence suggests that Tas2Rs are also expressed extraorally, including in airway smooth muscle cells, intestinal tuft cells, and immune cells, and their roles at these extraoral sites have been studied intensively in recent years (5).

Several groups have recently reported on the roles of Tas2Rs in the immune response. For example, mouse Tas2R138 (ortholog of human TAS2R38) in neutrophils binds with acyl-homoserine lactone (AHL)-12, a quorum sensing molecule (QSM) produced by *Pseudomonas aeruginosa* (*P. aeruginosa*). The study has indicated that the Tas2R138/AHL-12 binding promotes the degradation of lipid droplets to facilitate AHL-12 clearance from neutrophils *in vitro* and also shown suggestive evidence that the binding plays a role in protecting against bacterial infection using *P. aeruginosa* infection model mice (6). In addition, human TAS2R14 (ortholog of mouse Tas2R140) signaling induced by QSMs was found to enhance phagocytosis by macrophages *via* nitric oxide production and to promote the secretion of cytokines including IL-8, TNF-α, and IL-6 from gingival epithelial cells *in vitro* (7, 8). In contrast, other studies have suggested that Tas2R (or TAS2R) signaling suppresses immune responses (9–12), raising the possibility that each Tas2R subtype may play a different role in the immune system. Thus, further work is necessary to determine the pattern of expression of Tas2R subtypes in immune cells and to examine the contribution of these receptors in the immune response. Such an undertaking would be facilitated by the availability of Tas2R-deficient animal models. However, to the best of our knowledge, only two Tas2R-deficient mouse lines have been established: Tas2R105-deficient mice (13) and, more recently, triple-deficient mice that lack Tas2R126, Tas2R135, and Tas2R143 (*Tas2r126/135/143* ^−/−^) (14). The *Tas2r105* ^−/−^ mouse strain was shown to exhibit strong and selective impairment in the ability to taste one specific bitter taste substance, cycloheximide (15), whereas the *Tas2r126/135/143^−/−^* strain exhibited impaired responses to the Tas2R126/143 agonist d-salicin (16, 17) and the Tas2R135 agonist acesulfame potassium (16). However, whether these receptors are functionally expressed extraorally, including in immune cells, is unknown.

In the present study, we examined the expression profile of mouse Tas2R family members in immune cells *in silico* and then confirmed that *Tas2r126/135/143* mRNA is expressed at high levels in neutrophils relative to other immune cell types. In addition, we show that Tas2R126/143-mediated signaling enhances C-X-C motif chemokine ligand 2 (CXCL2)-stimulated migration of mouse neutrophils in a Rho-associated coiled-coil-containing protein kinase (ROCK)-dependent manner. This enhancement of migration was not observed in neutrophils from *Tas2r126/135/143^−/−^* mice, confirming that these Tas2R family members play a functional role in mouse neutrophils.

## Materials and methods

### Reagents

Arbutin, d-salicin, phenyl-β-d-glucopyranoside (PBD-Gluco), denatonium, and 5-propyl-2-thiouracil were purchased from Sigma-Aldrich (MO, USA). Recombinant CXCL2 was obtained from BioLegend (CA, USA), CXCL1 was from R&D system (MN, USA), N-formylmethionyl-leucyl-phenylalanine (fMLP) and Y-27632 were from Nacalai-Tesque (Kyoto, Japan), KD025 was from MedChemExpress (NJ, USA), and Tat-C3 was from Cytoskeleton (CO, USA). FITC-conjugated anti-Ly6G (1A8), APC/eFluor 780-anti-Ly6G (1A8), APC-anti-Ly6G (1A8), and PE-anti-CD11b (M1/70) monoclonal antibodies (mAbs) were purchased from Thermo Fisher Scientific (MA, USA). BV421-anti-CD8 (53-6.7), BV421-anti-F4/80 (BM8), Alexa Fluor 647-anti-CD4 (GK1.5), PE-anti-B220 (RA3-6B2), APC/Cy7-anti-Ly6G (1A) mAbs, and Dylight 649-anti-rabbit IgG polyclonal antibody (pAb) (Poly4064) were purchased from BioLegend. Anti-phospho-myosin light chain 2 (MLC2; Ser19) pAb was purchased from Cell Signaling Technology (MA, USA).

### Animals

Wild-type C57BL/6 mice were purchased from Japan SLC (Shizuoka, Japan) or Charles River Laboratories (Yokohama, Japan) and housed under specific pathogen-free conditions. *Tas2r126/135/143* heterozygous (^+/−^) or triple-deficient (^−/−^) mice were produced by introducing guide (g) RNAs and Cas9 enzyme (Thermos Fisher Scientific) into fertilized eggs using an electroporator (NEOA21, Nepagene, Chiba, Japan) as described previously (18). On-target and potential off-target sequences were screened using Benchling (https://benchling.com) or CRISPRdirect software (https://crispr.dbcls.jp/). The gRNA on-target sequences are listed in **Supplementary Table 1**. The resulting mice were screened by polymerase chain reaction (PCR) of genomic DNA followed by direct sequencing. Detailed genotypes are shown in **Figure 4**, and the PCR primer sequences are listed in **Supplementary Table 1**. The animal experimental protocols were approved by the Animal Research Committees of Wakayama Medical University, Niigata University, and Osaka University. *Tas2r126/135/143* ^-/-^ mouse line is available through the Riken BioResource Center (Riken BRC, Tsukuba, Japan; #11633) and the Center for Animal Resources and Development, Kumamoto University (CARD, Kumamoto, Japan; #3187).

### Cell purification

Macrophages were purified from the peritoneal cavity, neutrophils from bone marrow (BM), and T and B lymphocytes from spleens. For this, single cell suspensions were prepared in RPMI-1640 medium by gentle dissociation using 40-or 35-μm cell strainers. The cells were then stained by incubation with specific antibodies as described in section 2-1 for 30 min at 4°C, washed, and sorted using a FACSAria III flow cytometer (BD Biosciences, CA, USA). For some experiments, neutrophils were also purified from BM samples using an Easysep Mouse Neutrophil Enrichment Kit (STEMCELL Technologies, BC, Canada) or MojoSort Mouse Neutrophil Isolation Kit (BioLegend) in accordance with the manufacturers’ instructions. The purity of neutrophils isolated using the kits was approximately 80% (**Supplementary Figure 1**).

### Reverse transcription quantitative polymerase chain reaction (RT-qPCR)

RT-qPCR was performed as described previously (19) with several modifications. Total RNA was isolated from purified cell populations using a PureLink RNA isolation kit (Thermo Fisher Scientific) with PureLink DNase (Thermo Fisher Scientific), and first-strand cDNA was synthesized using M-MLV Reverse Transcriptase (Promega, WI, USA). qPCR was performed using a QuantStudio 7 Real-Time PCR system (Thermo Fisher Scientific) with TB Green^®^ Premix Ex Taq™ II (Takara Bio, Shiga, Japan) or SYBR^®^ Green Master Mix (Thermo Fisher Scientific). The qPCR primers have been described previously (19, 20) and are listed in **Supplementary Table 1**.

### Neutrophil migration assay

This assay was performed as described previously (21, 22) with minor modifications. Briefly, total BM cells were treated with ACK buffer to lyse red blood cells, then serum-starved in RPMI-1640 medium containing 0.1% bovine serum albumin (BSA) for 30 min, and then BM cells were stimulated with the indicated concentrations of the Tas2R agonists arbutin, d-salicin, denatonium, 5-propyl-2-thiouracil, and/or PBD-Gluco for 30 min. The cells were placed in the upper chamber of a 24-well Transwell plate containing 3.0-μm pore inserts (Corning, NY, USA). The chemoattractant CXCL2 (20 ng/ml), CXCL1 (20 ng/ml) or fMLP (3 μM) in RPMI-1640/0.1% BSA were then placed in the lower chambers and the plates were incubated for 1 h at 37°C in a CO_2_ incubator. The migrated cells were collected from the lower chamber, stained with APC/eFluor 780-anti-Ly6G and PE-anti-CD11b mAbs for 30 min at 4°C, and enumerated using a FACSVerse or FACSCelesta flow cytometer (BD Biosciences). Purified neutrophils and peritoneal neutrophils were also prepared for neutrophil migration assay. For purified neutrophils, the cells were collected from BM samples using the Easysep Mouse Neutrophil Enrichment Kit and used for migration assay as described above. For peritoneal neutrophils, mice were injected intraperitoneally (IP) with 200 μg zymosan (Wako pure Chemica, Osaka, Japan) in 0.5 ml sterile saline. 2 h after injection, the peritoneal cells were collected and used for migration assay as described above. For the ROCK inhibitor experiments, serum-starved BM cells were treated with 10 μM Y-27632 or 20 μM KD025 in the presence or absence of arbutin for 30 min at 37°C before being placed in the upper chamber. For the RhoA inhibitor experiments, serum-starved BM cells were treated with 0.5 μg/ml Tat-C3 for 4 h at 37°C before pretreatment of arbutin.

### Zymosan-induced peritonitis model *in vivo*

Mice were injected IP with zymosan (Wako pure Chemica) in 0.5 ml sterile saline. 6 h after injection, the peritoneal cavities were washed with 2 ml ice cold PBS. The neutrophils in the peritoneal lavage fluid were stained with APC/eFlour 780-anti-Ly6G and PE-anti-CD11b for analysis by flow cytometry. Subsequently, the number of neutrophils was determined using Count Bright Absolute Counting Beads (Thermo Fischer Scientific).

### FACS analysis of CXCR2 and phosphorylated MLC2 expression levels

CXCR2 expression was analyzed by staining of total BM cells with FITC-anti-Ly6G, PE-anti-CD11b, and Alexa Fluor 647-anti-CXCR2 mAbs for 30 min at 4°C. Phosphorylated MLC2 levels were determined using neutrophils purified with kits as described above. The purified cells were serum-starved in RPMI-1640/0.1% BSA for 4 h, treated with arbutin for 30 min, and then stimulated with CXCL2 for 2 min. The cells were fixed with Fixation Buffer (BioLegend) for 30 min, permeabilized with 0.1% Triton-X for 10 min, and stained with anti-phospho-MLC2 pAb for 18 h at 4°C. After washing, the cells were incubated with Dylight 649-anti-rabbit IgG pAb for 60 min at room temperature. Data were acquired using a FACSCelesta flow cytometer and analyzed with FlowJo software (Tree Star Inc. OR, USA).

### Neutrophil polarization assay

Neutrophil polarization assay was performed using neutrophils purified with kits as described above. The purified neutrophils were serum-starved in RPMI-1640/0.1% BSA for 4 h, treated with or without arbutin for 30 min at 37°C in an 8-well chamber slide (Merck, CA, USA), and then stimulated with CXCL2 for 2 min at 37°C. The cells were fixed with 4% PFA (Wako pure Chemica) for 10 min, permeabilized with 0.1% Triton-X for 10 min, and then stained with Flash Phalloidin Green 488 (BioLegend) for 20 min at room temperature. The nucleus was stained with DAPI (Nacalai-Tesque). The images were randomly acquired using an FV1200 confocal laser microscope with a 40x objective lens (Olympus, Tokyo, Japan). The number of total neutrophils was counted with Duolink Image Tool software (Sigma). Polarized neutrophils were determinized by nonuniform phalloidin staining (F-actin).

### Statistical analysis

Data are presented as the mean ± standard deviation (SD) or standard error of the mean (SEM). Statistical analysis was performed as described previously (22) using Prism software (GraphPad, CA, USA). Briefly, differences between groups were evaluated by Student’s *t*-test for single comparisons or by one-way analysis of variance (ANOVA) followed by Tukey’s *post hoc* test for multiple comparisons.

## Results

### Neutrophils express *Tas2r126*, *Tas2r135*, and *Tas2r143*

**Figure 1:**
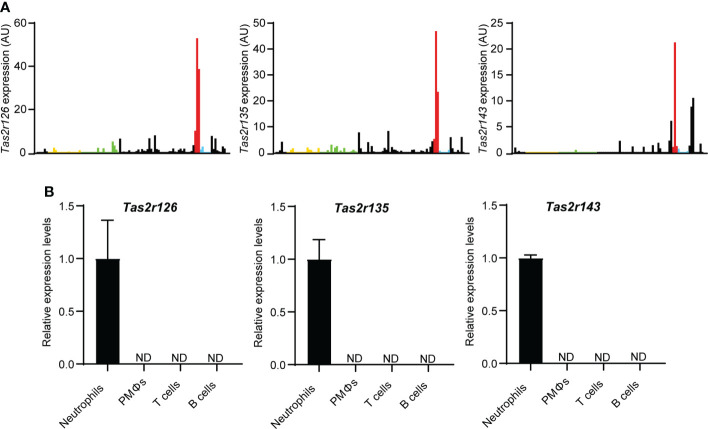
Neutrophils express *Tas2r126*, *Tas2r135*, and *Tas2r143*. **(A)** Analysis of *Tas2r* expression in immune cells from the ImmGen RNA-seq dataset. The data shown is *Tas2r126*, *Tas2r135*, and *Tas2r143* mRNA levels in immune cells. Yellow, green, red, and blue bars show *Tas2r126*, *Tas2r135*, or *Tas2r143* mRNA expression in B cells, T cells, neutrophils, or macrophages. Black bars show the mRNA expression in immune cells other than B cells, T cells, neutrophils, and macrophages. **(B)** RT-qPCR analysis of *Tas2r126*, *Tas2r135*, and *Tas2r143* mRNA levels in purified spleen-derived B220^+^ B cells or CD4^+^/CD8^+^ T cells; F4/80^+^/CD11b^+^ peritoneal macrophages (PMΦs); and bone marrow-derived CD11b^+^/Ly6G^+^ neutrophils. Data are presented as the mean ± SD of triplicates from one experiment and are representative of three independent experiments. ND, not detected.

To explore the expression of Tas2Rs in immune cells, we surveyed 36 mouse *Tas2r* genes by analysis of the Immunological Genome Project (ImmGen, https://www.immgen.org/) RNA-seq database. As shown in **Figure 1A** and **Supplementary Data Set 1**, *Tas2r126*, *Tas2r135*, and *Tas2r143* were highly expressed in neutrophils compared with other immune cell types, whereas other *Tas2rs* were low expressed in neutrophils. To confirm *Tas2r126*, *Tas2r135*, and *Tas2r143* expression in neutrophils, we performed RT-qPCR analysis of FACS-sorted neutrophils isolated from BM, macrophages isolated from the peritoneal cavity, and T and B cells from the spleen of wild-type C57BL/6 mice. We detected *Tas2r126, Tas2r135*, and *Tas2r143* mRNAs in neutrophils, whereas expression was undetectable in the other cells examined (**Figure 1B**). Thus, *Tas2r126/135/143* are expressed in mouse neutrophils, consistent with a previous report (23).

### Tas2R126/143 agonists augment chemoattractant-induced neutrophil migration

**Figure 2:**
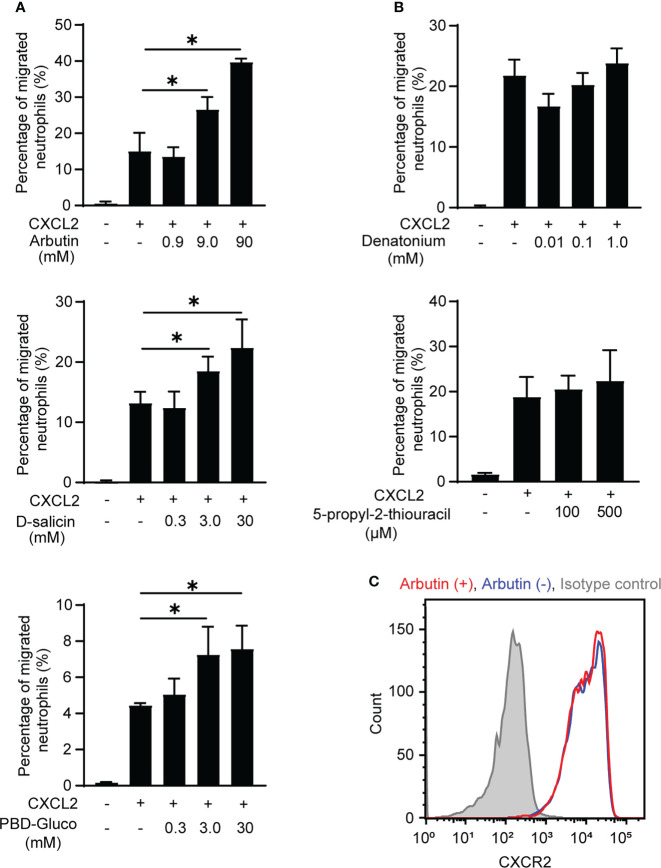
Effects of Tas2R126/135/143 agonists on CXCL2-induced neutrophil migration. **(A)** Transwell migration assays of bone marrow (BM)-derived neutrophils from wild-type C57BL/6 mice after pretreatment with the indicated concentrations of the Tas2R126/143 agonists arbutin and d-salicin or the Tas2R126 agonist PBD-Gluco for 30 min followed by stimulation with 20 ng/ml CXCL2. Data are presented as the mean ± SD of triplicates from one experiment and are representative of three independent experiments. *p < 0.05 by Student’s *t* test. **(B)** Transwell migration assays of BM-derived neutrophils from wild-type C57BL/6 mice after pretreatment with the Tas2R135 agonists denatonium and 5-propyl-2-thiouracil for 30 min followed by stimulation with 20 ng/ml CXCL2. Data are presented as the mean ± SD of triplicates from one experiment and are representative of three independent experiments. **(C)** FACS analysis of CXCR2 expression on purified neutrophils incubated with (red) or without (blue) arbutin. Gray histogram shows the isotype control-stained cells. Data are representative of three independent experiments.

Neutrophils are the most motile cells in higher organisms and efficiently migrate in response to a chemoattractant gradient (24). Therefore, we next performed classical Transwell migration assays to investigate the effects of Tas2R agonists on BM-derived neutrophil migration. We selected the agonists arbutin, d-salicin (both Tas2R126/143 agonists), PBD-Gluco (Tas2R126 agonist), denatonium, and 5-propyl-2-thiouracil (both Tas2R135 agonists) for this study on the basis of a previous report of the 50% effective concentrations (EC_50_) for these compounds (16). However, our preliminary data suggested that treatment of neutrophils with these Tas2R agonists alone had very little effect on migration (**Supplementary Figure 2**). Therefore, we examined whether pretreatment with the agonists affected migration induced by the chemoattractant CXCL2. Indeed, the Tas2R126/143 agonists arbutin (16, 25) and d-salicin (16, 17), and the Tas2R126 agonist PBD-Gluco (16) all significantly enhanced CXCL2-induced neutrophil migration (**Figure 2A**), whereas the Tas2R135 agonists denatonium and 5-propyl-2-thiouracil had no significant effect (**Figure 2B**). We next examined whether Tas2R126/143 agonists affect CXCL2 receptor CXCR2 expression. The most potent agonist, arbutin, had no effect on expression of CXCR2 on the surface of neutrophils (**Figure 2C**). In addition, arbutin did not affect CXCL2-induced neutrophil polarization (**Supplementary Figure 3**). Thus, our results suggest that agonists of Tas2R126/143, but not Tas2R135, enhance CXCL2-stimulated neutrophil migration without modulating CXCR2 expression and cell polarity.

**Figure 3:**
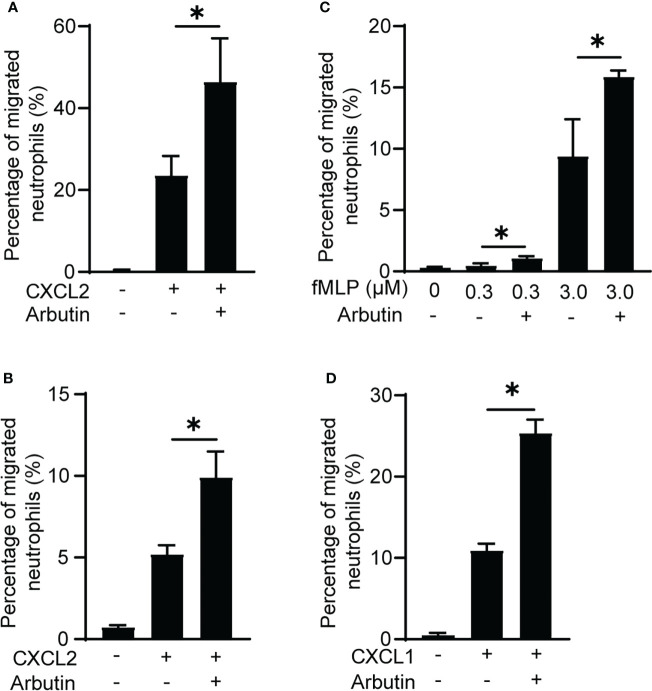
Arbutin enhances neutrophil migration. **(A, B)** Effects of arbutin on purified neutrophil **(A)** or peritoneal neutrophil **(B)** migration to CXCL2 was assessed by Transwell assay. Data are presented as the mean ± SD of triplicates from one experiment and are representative of two independent experiments. *p < 0.05 by Student’s *t* test. **(C, D)** Neutrophil migration in response to fMLP **(C)** or CXCL1 **(D)** with or without arbutin was evaluated by Transwell assay. Data are presented as the mean ± SD of triplicates from one experiment and are representative of two independent experiments. *p < 0.05 by Student’s *t* test.

To rule out the possibility that BM cells other than neutrophils were involved in the enhancement of neutrophil migration by Tas2R126/143 agonists, we used purified neutrophils. As we expected, arbutin enhanced CXCL2-induced purified neutrophil migration (**Figure 3A**). We also found that arbutin enhanced peritoneal neutrophil migration (**Figure 3B**). These results support the idea that arbutin directly acts on neutrophils and enhances CXCL2-induced neutrophil migration. To address whether arbutin affected other chemoattractants-induced neutrophil migration, we analyzed the effect of arbutin on fMLP- or CXCL1-induced neutrophil migration. As shown in **Figures 3C, D**, pretreatment of arbutin significantly enhanced fMLP- or CXCL1-induced neutrophil migration. These observations suggest that arbutin generally enhances chemoattractant-induced neutrophil migration.

### Tas2R agonist-mediated enhancement of neutrophil migration is abolished by deletion of *Tas2r126/135/143* expression

**Figure 4:**
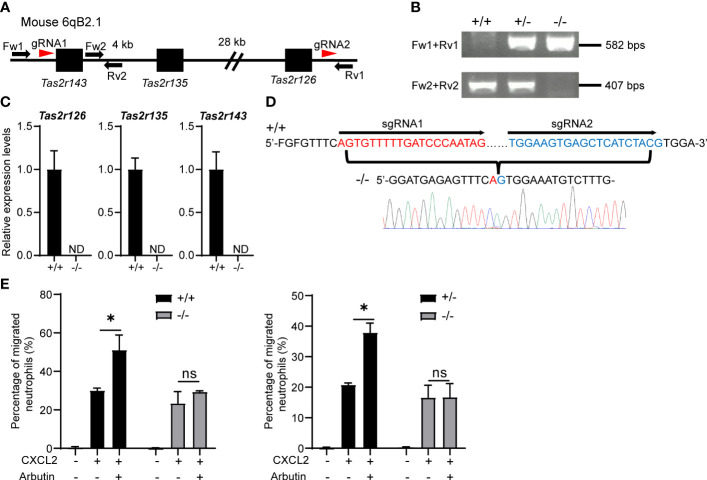
Arbutin-mediated enhancement of CXCL2-stimulated neutrophil migration is attenuated by deletion of *Tas2r126, Tas2r135*, and *Tas2r143*. **(A)** Schematic showing the location of *Tas2r126, Tas2r135*, and *Tas2r143* genes on mouse chromosome 6qB2.1 and the position of gRNAs designed for deletion of the gene cluster between *Tas2r143* and *Tas2r126* (red arrowheads). Fw1, Fw2, Rv1, and Rv2 indicate the primers used for PCR genotyping of the mice. **(B)** DNA fragments amplified from *Tas2r126/135/143* ^+/+^, ^+/−^, and ^−/−^ mice. **(C)** RT-qPCR analysis of *Tas2r126*, *Tas2r135*, and *Tas2r143* mRNA expression levels in bone marrow (BM)-derived neutrophils from *Tas2r126/135/143* ^+/+^ or ^−/−^ mice. Data are presented as the mean ± SD of triplicates from one experiment and are representative of three independent experiments. ND, not detected. **(D)** DNA sequences of PCR amplicons. A total of 35 kb of the genomic region between *Tas2r143* and *Tas2r126* was deleted in the *Tas2r126/135/143* ^−/−^ mice. **(E)** Transwell migration assays of BM-derived neutrophils from *Tas2r126/135/143* ^+/+^ and ^−/−^ mice (left panel) or ^+/−^ and ^−/−^ mice (right panel). Cells were pretreated with 90 mM arbutin for 30 min and then stimulated with 20 ng/ml CXCL2. Data are presented as the mean ± SD of triplicates from one experiment and are representative of three independent experiments. *p < 0.05 by Student’s *t* test. ns, not significant.

To exclude the possibility that arbutin enhanced neutrophil migration in a Tas2R126/143-independent manner, we generated lines of triple-deficient *Tas2r126/135/143^−/−^* mice. These three Tas2Rs are all located on mouse chromosome 6qB2.1; therefore, we deleted the region between *Tas2r143* and *Tas2r126* by introducing two gRNAs and Cas9 into fertilized eggs of C57BL/6 mice (**Figure 4A**). Effective deletion of the genes was confirmed by PCR of genomic DNA (**Figure 4B**), RT-qPCR of BM-derived neutrophils (**Figure 4C**), and direct gene sequencing (**Figure 4D**). As shown in **Supplementary Figure 4**, the expression of *Tas2rs* in heterozygous (^+/−^) and wild-type control (^+/+^) were almost comparable levels. Importantly, arbutin-mediated augmentation of CXCL2-stimulated migration was abolished in neutrophils from homozygous *Tas2r126/135/143* ^−/−^ mice but not those from heterozygous (^+/−^) or wild-type control (^+/+^) mice (**Figure 4E**). These results confirm that arbutin promotes chemoattractant-induced neutrophil migration *via* Tas2R126 and/or Tas2R143 signaling.

### ROCK signaling is involved in Tas2R agonist-mediated enhancement of neutrophil migration

**Figure 5:**
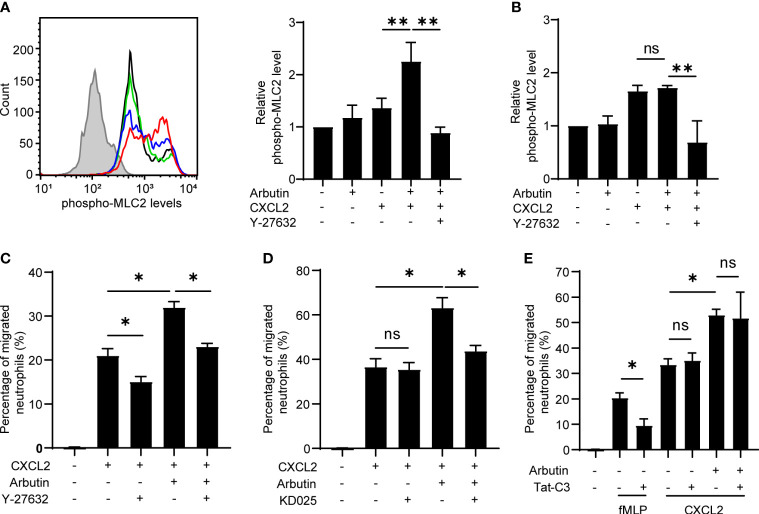
The ROCK–MLC2 pathway is involved in arbutin-mediated enhancement of CXCL2-stimulated neutrophil migration. **(A)** Left panel: FACS histograms of phosphorylated MLC2 expression levels in bone marrow (BM)-derived neutrophils from wild-type C57BL/6 mice. Neutrophils were untreated (black line) or incubated with CXCL2 alone (blue line), arbutin (green line), or both arbutin and CXCL2 (red line), Gray histogram shows control cells (anti-phospho-MLC2 pAb omitted). Right panel: Quantification of phosphorylated MLC2 expression (median fluorescence intensity) in the treated cells. Cells were also analyzed after stimulation with CXCL2 in the presence of Y-27632. Data are presented as the mean ± SD of three to five independent experiments. **p < 0.01 by Student’s *t* test. **(B)** Quantification of phosphorylated MLC2 expression (median fluorescence intensity) in BM-derived neutrophils from *Tas2r126/135/143* ^−/−^ mice after treatment with arbutin and CXCL2. Data are presented as the mean ± SD of three to six independent experiments. **p < 0.01 by Student’s *t* test. **(C, D)** Transwell migration assays of BM-derived neutrophils from *Tas2r126/135/143^+/+^* mice after pretreatment with arbutin and either Y-27632 **(C)** or KD025 **(D)** (both ROCK inhibitors) for 30 min followed by stimulation with CXCL2. Data are presented as the mean ± SD of triplicates from one experiment and are representative of three independent experiments. *p < 0.05 by Student’s *t* test. **(E)** Transwell migration assay of BM-derived neutrophils from *Tas2r126/135/143* ^+/+^ mice after pretreatment with arbutin and/or the RhoA inhibitor Tat-C3 followed by stimulation with fMLP or CXCL2. Data are presented as the mean ± SD of triplicates from one experiment and are representative of three independent experiments. ns, not significant; *p < 0.05 by Student’s *t* test.

Previous studies have shown that bitter taste substances induce smooth muscle contraction and the studies have indicated the involvement of ROCK signaling (26) and MLC activation (27) in the smooth muscle contraction. In addition, we and others have reported that ROCK-dependent MLC2 activation plays a critical role in immune cell migration (28, 29). Therefore, we examined whether the ROCK–MLC2 pathway is involved in Tas2R126/143 signaling for enhanced neutrophil migration. Phosphorylated MLC2 (pMLC2) levels were analyzed and quantified by intracellular staining and flow cytometry of treated neutrophils. We found that pMLC2 levels were not affected by treatment of cells with arbutin alone and were slightly but not significantly increased by CXCL2 alone; however, combination treatment with arbutin and CXCL2 resulted in a striking increase in pMLC2 levels (**Figure 5A**). Moreover, this increase in pMLC2 was competently inhibited by co-treatment of neutrophils with the ROCK1/2 inhibitor, Y-27632 (**Figure 5A**). Notably, the arbutin-mediated increase in pMLC2 levels was not observed in neutrophils from *Tas2r126/135/143* ^−/−^ mice (**Figure 5B**), indicating that arbutin activates ROCK-mediated phosphorylation of MLC2 in neutrophils in a CXCL2- and Tas2R126/143-dependent manner.

To assess the involvement of ROCK signaling in arbutin-enhanced neutrophil migration, we pretreated cells with the ROCK1/2 inhibitor Y-27632 or the ROCK2-selective inhibitor KD025 and examined CXCL2-stimulated migration in the presence or absence of arbutin. Notably, Y-27632, but not KD025, significantly attenuated migration induced by CXCL2 alone, whereas both compounds significantly suppressed arbutin-enhanced CXCL2-dependent migration (**Figures 5C, D**). These results suggest that CXCL2-induced neutrophil migration is likely mediated *via* ROCK1 alone, whereas arbutin-dependent augmentation most likely involves ROCK2. Because ROCK signaling is regulated by the upstream small G protein RhoA (30), we also examined the effect of RhoA inhibition on arbutin-enhanced neutrophil migration. As shown in **Figure 5E**, treatment with the RhoA inhibitor Tat-C3 inhibited migration of cells stimulated by fMLP, but not by CXCL2, similar to a previous report (31). Moreover, Tat-C3 also had no effect on arbutin-enhanced neutrophil migration in response to CXCL2 (**Figure 5E**). Based on these results, we propose a model in which Tas2R126/143 signaling enhances CXCL2-stimulated neutrophil migration *via* a ROCK2-dependent and RhoA-independent mechanism.

## Discussion

Tas2Rs are expressed in the oral cavity where they detect bitter taste substances that may be harmful to health. Increasing evidence indicates that Tas2Rs are also expressed extraorally (5) and, although Tas2Rs expressed in immune cells are assumed to play roles in host defense, this has not been studied in detail. Here, we determined that neutrophils express *Tas2r126, Tas2r135*, and *Tas2r143*, and Tas2R126/143 play a role in promoting neutrophil migration. Specifically, we found that Tas2R126/143 agonists enhanced the chemoattractant-induced neutrophil migration *via* a mechanism involving RhoA-independent ROCK activation and MLC2 phosphorylation. This function of Tas2R126/143 was confirmed by demonstrating that the effects of Tas2R agonists on neutrophil migration and pMLC2 production were markedly diminished in neutrophils isolated from *Tas2r126/135/143*-deficient mice.

Currently, little is known about Tas2R signaling (32). Our results indicate that arbutin facilitates neutrophil migration *via* a Tas2R126/143–ROCK2–MLC2 pathway, which is consistent with findings that bitter taste substances induce ROCK-dependent smooth muscle contraction and MLC phosphorylation in smooth muscle cells (26, 27). As shown in **Figure 5A**, compared to CXCL2 alone, the combination treatment with arbutin and CXCL2 increased pMLC2 levels in a specific neutrophil population rather than total neutrophils. These results likely show that both Tas2R agonist-responsive and nonresponsive neutrophil populations exist. Although our data indicate that Tas2R signaling regulates ROCK2 and pMLC2 in a CXCL2-dependent manner, the precise molecular mechanisms underlying this sequence of events remain to be clarified.

Recent evidence suggests that bitter taste substances produced by pathogens can elicit immune responses. For example, a QSM, heptyl-hydroxyquinoline secreted by *Pseudomonas* was shown to enhance bacterial phagocytosis by human macrophages *via* TAS2R14-dependent nitric oxide production (8). In addition, other QSMs, competence-stimulating peptides secreted by *Streptococcus mutans* can induce secretion of proinflammatory cytokines from human gingival epithelial cells *via* TAS2R14 (7). Another group showed that substances excreted/secreted by the parasitic helminth *Trichinella spiralis* can induce IL-25 production by mouse tuft cells *via* Tas2R-mediated signaling, and, in turn, IL-25 triggers a type 2 immune response and leads to a “weep and sweep” response in the gut (17). Taking these reports into consideration, we speculate that several bitter taste substances produced by pathogens may be potent Tas2R126/143 agonists. The Tas2R126 agonists arbutin, d-salicin, and PBD-Gluco are commonly classified as β-d glucopyranosides (16), and several pathogens, especially fungi, are known to produce β-d glucopyranosides (33, 34). Thus, fungal β-d glucopyranosides could be considered to be members of the pathogen-associated molecular pattern family of molecules that promote responses by innate immune cells; as such, Tas2R126/143 would function as pattern recognition receptors.

To the best of our knowledge, only low-affinity agonists for Tas2R126/143 have been reported to date. Previous studies have reported EC_50_s of 30 mM, 10 mM, and 10 mM arbutin, d-salicin, and PBD-Gluco, respectively, for activation of Tas2R126, and of 3 mM and 0.3 mM arbutin and d-salicin, respectively, for activation of Tas2R143 (16, 17, 25). However, high-affinity agonists have been reported for human TAS2R14; they include heptyl-hydroxyquinoline and competence-stimulating peptides, which activated human TAS2R14 at concentrations of 20 μM (8) and 50 μM (7), respectively. Thus, we speculate that additional pathogen-derived substances may be identified that are high-affinity agonists of Tas2R126/143 and might play additional roles in promoting neutrophil functions, including migration. However, further work will be necessary to establish this.

In humans, β-d glucopyranosides including arbutin, d-salicin, and PBD-Gluco are recognized by TAS2R16 (ortholog of mouse Tas2R118) (35). While the ortholog of mouse *Tas2r143* is a pseudogene in humans, the ortholog of mouse *Tas2r126* is considered to be human *TAS2R41* (16, 36). As TAS2R41 agonist, an antibacterial drug, chloramphenicol, is the only reported (37), and is not classified as a β-d glucopyranoside. Using the neutrophil RNA-seq database (Gene Expression in Neutrophil-like Cell Lines, https://collinslab.ucdavis.edu/neutrophilgeneexpression/) (38), we analyzed mouse *Tas2r126/135*, human *TAS2R41*, human *TAS2R16*, and human *TAS2R60* (ortholog of mouse *Tas2r135*). We found the expression of *TAS2R41* and *TAS2R60* in human neutrophils, though the expression levels of *TAS2R41/60* were lowered compared with mouse *Tas2r* counterparts (**Supplementary Figure 5**). In contrast, *TAS2R16* was at undetectable levels in human neutrophils. These data suggest that the enhancement of neutrophil migration by β-d glucopyranosides may be restricted in specific species such as mice. Given that chloramphenicol is produced by several gram-positive soil actinomycetes (39), TAS2R41 expressed on human neutrophils might sense the bacteria products such as chloramphenicol. These points are fascinating, and future studies should be focused on them.

There are several limitations to this study. First, although we evaluated the effect of arbutin on neutrophil infiltration *in vivo* using the zymosan-induced peritonitis model, we observed no effect of *Tas2r126/135/143* deletion in this model (**Supplementary Figure 6**), suggesting that other mechanisms of arbutin-facilitated neutrophil infiltration exist, at least *in vivo*. Second, we examined neutrophils from triple-deficient *Tas2r126/135/143^−/−^* mice, and it is unclear whether Tas2R126, Tas2R143, or both receptors participate in arbutin-induced enhancement of neutrophil migration. Third, it is unclear whether Tas2R126/143-mediated signaling also affects other crucial neutrophil functions, including phagocytosis, production of reactive oxygen species, and neutrophil extracellular trap formation. Since our current study assessed the effect of Tas2R agonists in a short period (0.5-1.5 h), the longer-term effects of Tas2R agonists on neutrophil function also remains uncertain. Finally, although we showed that neutrophils express *Tas2r135* mRNA, the Tas2R135 agonists denatonium and 5-propyl-2-thiouracil had no effect on CXCL2-induced migration; therefore, it is unclear whether neutrophil-expressed Tas2R135 protein is functional. As another possibility, although neutrophils expressed Tas2R135 at functional levels, the downstream molecules of Tas2R135 might differ from Tas2R126/143 in neutrophils, and thus Tas2R135 agonists might have no effect on neutrophil migration.

Collectively, the results of this study provide evidence that Tas2R126 and/or Tas2R143 signaling induced by exogenous bitter taste substances enhance chemoattractant-induced mouse neutrophil migration *via* ROCK and MLC2-dependent signaling.

## Data availability statement

The datasets presented in this study can be found in online repositories. The names of the repository/repositories and accession number(s) can be found in the article/**Supplementary Material**.

## Ethics statement

The animal study was reviewed and approved by Osaka University, Wakayama Medical University, and Niigata University.

## Author contributions

DK conceived and designed the study; acquired, analyzed, and interpreted the data; and wrote the first draft of the article. TK analyzed, and interpreted the data; and wrote the first draft of the article. TW, MO, and YK acquired, analyzed and interpreted the data. FS, NK, AT, MI, and SM analyzed and interpreted the data. All authors contributed to the article and approved the submitted version.

## Funding

This work was supported by funding from the Japan Dairy Association (J-milk), a Niigata University Interdisciplinary Research (U-go) Grant, the Smoking Research Foundation, Fuji Foundation for Protein Research, and JSPS KAKENHI grants numbers 19K16697, 22K17823, and JP16H06276 (AdAMS).

## Acknowledgments

We thank Dr. Masayuki Miyasaka (Osaka University) for his support of this research. We thank Ms. Eri Hosoyamada (Osaka University) for her technical assistance. We thank Anne M. O’Rourke, PhD, from Edanz (https://jp.edanz.com/ac) for editing a draft of this manuscript.

## Conflict of interest

The authors declare that the research was conducted in the absence of any commercial or financial relationships that could be construed as a potential conflict of interest.

## Publisher’s note

All claims expressed in this article are solely those of the authors and do not necessarily represent those of their affiliated organizations, or those of the publisher, the editors and the reviewers. Any product that may be evaluated in this article, or claim that may be made by its manufacturer, is not guaranteed or endorsed by the publisher.

## Supplementary material

Supplementary Figure 1 The purity of neutrophils after isolation from BM samples. Neutrophils were purified from BM samples by Easysep Mouse Neutrophil Enrichment Kit **(A)** or MojoSort Mouse Neutrophil Isolation Kit **(B)**. The purity of neutrophils was assessed by FACS analysis.

Supplementary Figure 2 Tas2R agonists alone do not affect neutrophil migration. Neutrophil migration in the presence or absence of Tas2R agonists in the upper chamber was examined by Transwell assay. Neutrophil migration to CXCL2 added to the lower chamber was used as a positive control. Data are presented as the mean ± SD of triplicates from one experiment and are representative of two independent experiments. **p* < 0.05 by Student’s *t* test.

Supplementary Figure 3 Effect of arbutin on CXCL2-induced neutrophil polarization. **(A)** Polarized neutrophils after stimulation with CXCL2 in the presence or absence of arbutin were counted (Green; F-actin, Blue; nucleus). White arrowheads show polarized neutrophils. **(B)** Quantification of the percentage of polarized neutrophils. Data are presented as the mean ± SD of four fields. The result shown is representative of two independent experiments, and per experiment 203 or 388 neutrophils (control), 299 or 630 neutrophils (arbutin), 1542 or 2452 neutrophils (CXCL2), and 759 or 1528 neutrophils (arbutin and CXCL2) were evaluated. NS, not significant. Scale bar, 20 μm.

Supplementary Figure 4 Expression levels of *Tas2r126/135/143* in *Tas2r126/135/143* ^+/+^ and ^+/−^ mice derived neutrophils. RT-qPCR analysis of *Tas2r126*, *Tas2r135*, and *Tas2r143* mRNA expression levels in purified neutrophils from *Tas2r126/135/143* ^+/+^ or ^+/−^ mice. Data are presented as the mean ± SD of triplicates from one experiment and are representative of two independent experiments.

Supplementary Figure 5 Ortholog relationships between *mouse Tas2rs* and *human TAS2Rs*. **(A)** Human *TASR41* and human *TAS2R60* are ortholog of mouse *Tas2r126* and mouse *Tas2r135*, respectively. Mouse *Tas2r143* is a pseudogene in human (*TAS2R62P*). Human *TAS2R41/60/62P* are all located on human chromosome 7q35. **(B)** Expression levels of *TAS2R41* and *TAS2R60* in human neutrophils were analyzed by using the RNA-seq database.

Supplementary Figure 6 Arbutin enhances neutrophil infiltration in a zymosan-induced peritonitis model in a Tas2R126/135/143-independent manner. **(A)** Wild-type C57BL/6 mice were injected IP with the indicated amount of zymosan in 0.5 ml sterile saline. Infiltrated neutrophils in peritoneal lavage fluid were collected 6 h later and enumerated by FACS analysis. Data represent the mean ± SEM of three mice. **(B, C)** Wild-type C57BL/6 mice **(B)** and *Tas2r126/135/143* ^−/−^ mice **(C)** were injected IP with saline, 2 μg zymosan, or 2 μg zymosan with 25 mg arbutin, and infiltered neutrophils were enumerated 6 h later by FACS analysis. Data represent the mean ± SEM of 5–9 mice. **p* < 0.05 by Student’s *t* test. ***p* < 0.05 by one-way ANOVA followed by Tukey’s multiple comparison test.

Supplementary Table 1 List of gRNA on-target sequences and PCR primer sequences.

Supplementary Data Set 1 Analysis of 36 mouse *Tas2r* gene expression levels in immune cells by ImmGen RNA-seq database.
