# Supplementary figures and images for: Tas2R signaling enhances mouse neutrophil migration via a ROCK-dependent pathway
Source: Front Immunol. 2022 Aug 18;13:973880. doi: 10.3389/fimmu.2022.973880 (PMC9436316; doi:10.3389/fimmu.2022.973880)

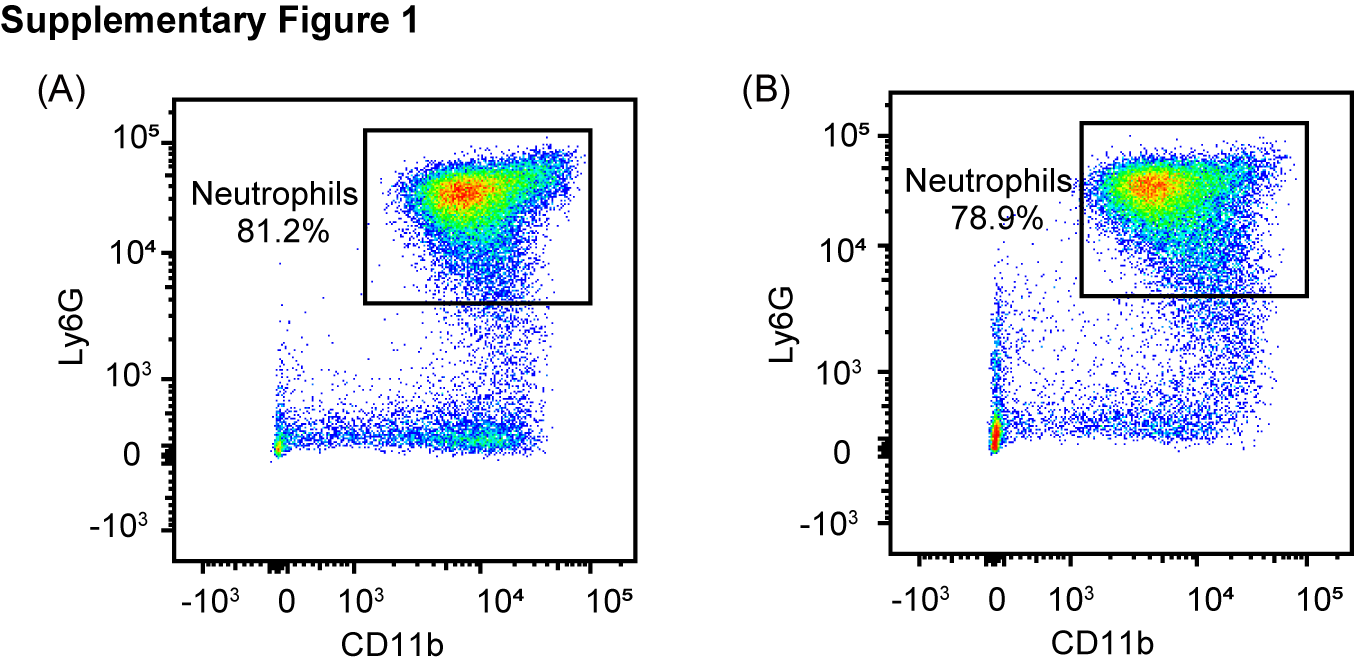

Supplement: Supplementary Figure 1 — The purity of neutrophils after isolation from BM samples. Neutrophils were purified from BM samples by Easysep Mouse Neutrophil Enrichment Kit (A) or MojoSort Mouse Neutrophil Isolation Kit (B). The purity of neutrophils was assessed by FACS analysis. [file Image_1.tif]

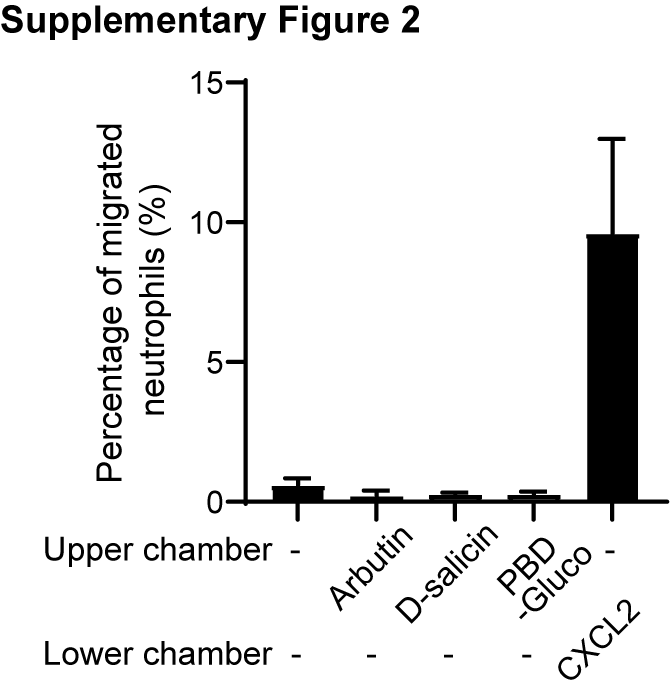

Supplement: Supplementary Figure 2 — Tas2R agonists alone do not affect neutrophil migration. Neutrophil migration in the presence or absence of Tas2R agonists in the upper chamber was examined by Transwell assay. Neutrophil migration to CXCL2 added to the lower chamber was used as a positive control. Data are presented as the mean ± SD of triplicates from one experiment and are representative of two independent experiments. *p < 0.05 by Student’s t test. [file Image_2.tif]

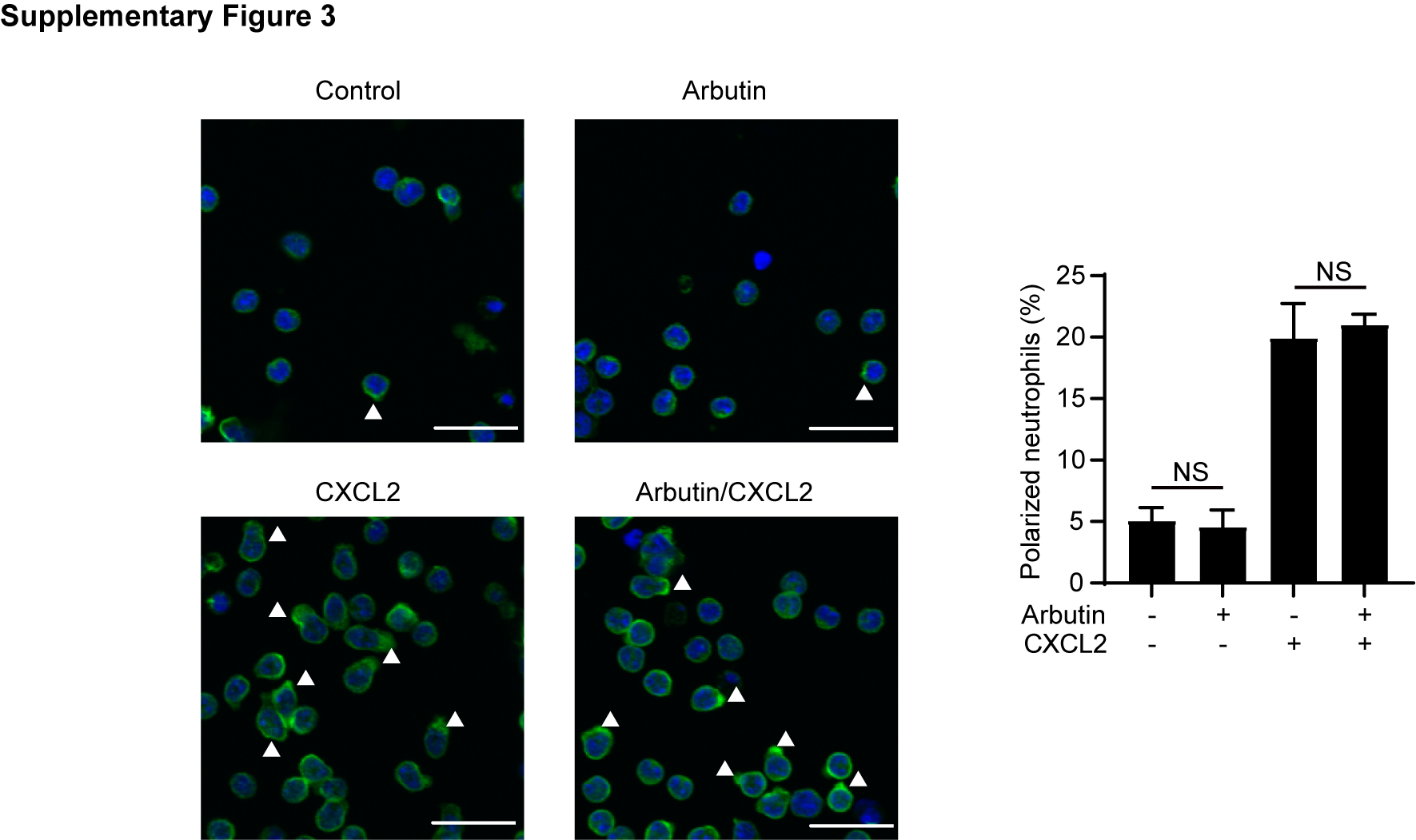

Supplement: Supplementary Figure 3 — Effect of arbutin on CXCL2-induced neutrophil polarization. (A) Polarized neutrophils after stimulation with CXCL2 in the presence or absence of arbutin were counted (Green; F-actin, Blue; nucleus). White arrowheads show polarized neutrophils. (B) Quantification of the percentage of polarized neutrophils. Data are presented as the mean ± SD of four fields. The result shown is representative of two independent experiments, and per experiment 203 or 388 neutrophils (control), 299 or 630 neutrophils (arbutin), 1542 or 2452 neutrophils (CXCL2), and 759 or 1528 neutrophils (arbutin and CXCL2) were evaluated. NS, not significant. Scale bar, 20 μm. [file Image_3.tif]

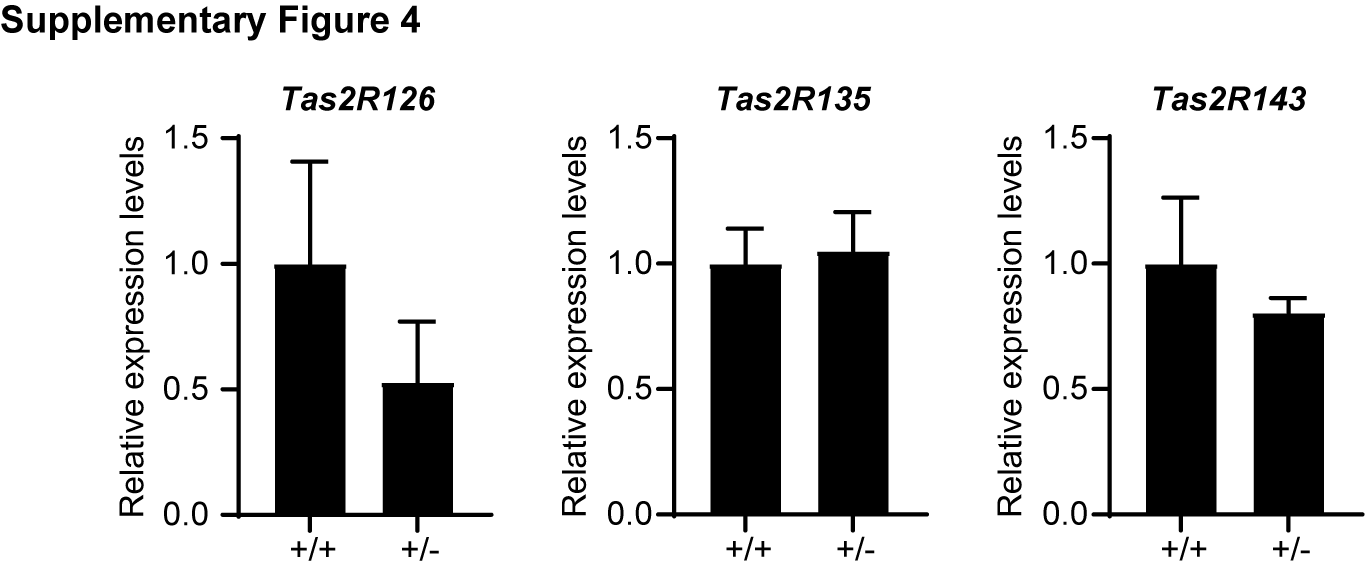

Supplement: Supplementary Figure 4 — Expression levels of Tas2r126/135/143 in Tas2r126/135/143 +/+ and +/− mice derived neutrophils. RT-qPCR analysis of Tas2r126, Tas2r135, and Tas2r143 mRNA expression levels in purified neutrophils from Tas2r126/135/143 +/+ or +/− mice. Data are presented as the mean ± SD of triplicates from one experiment and are representative of two independent experiments. [file Image_4.tif]

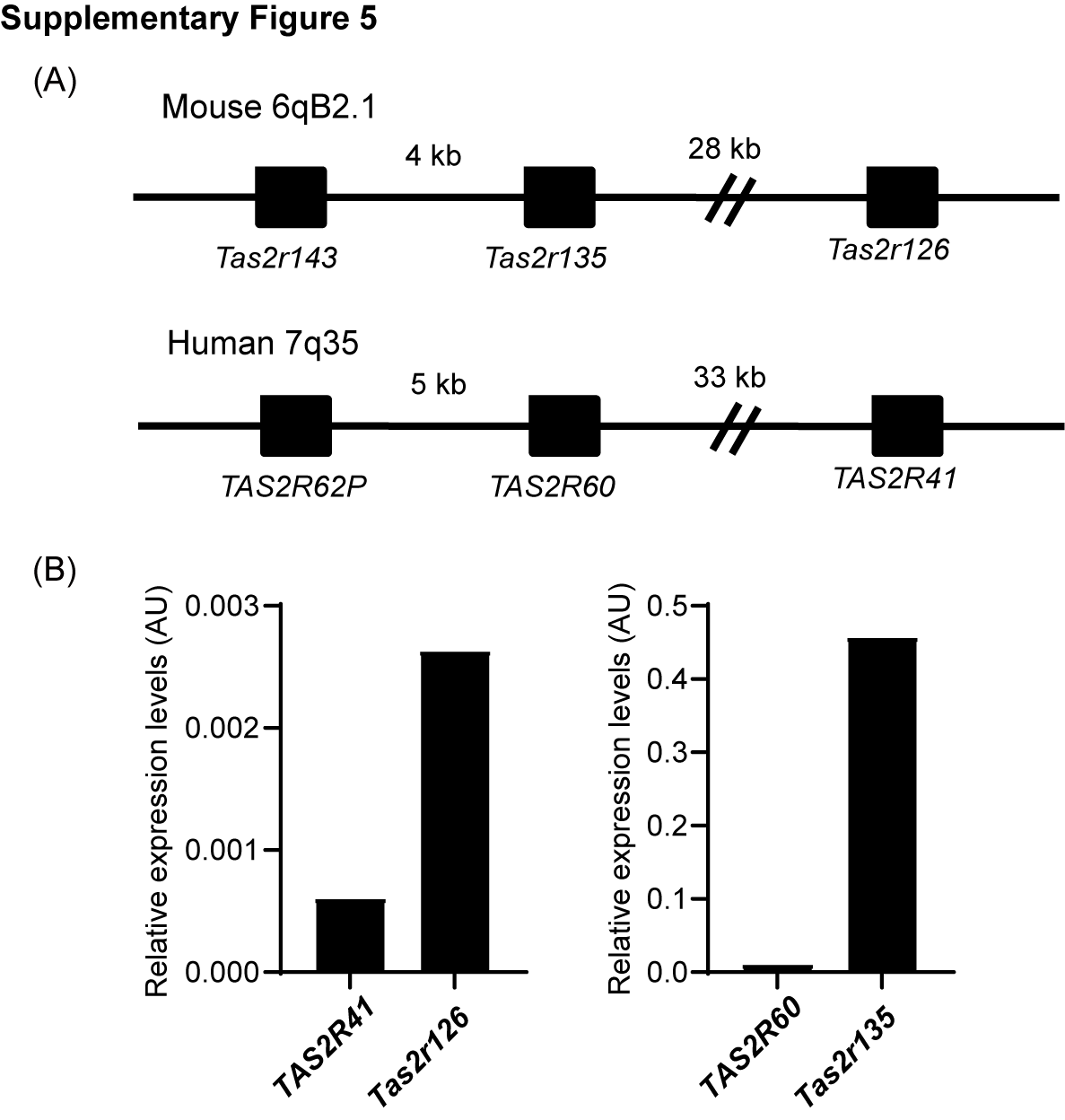

Supplement: Supplementary Figure 5 — Ortholog relationships between mouse Tas2rs and human TAS2Rs. (A) Human TASR41 and human TAS2R60 are ortholog of mouse Tas2r126 and mouse Tas2r135, respectively. Mouse Tas2r143 is a pseudogene in human (TAS2R62P). Human TAS2R41/60/62P are all located on human chromosome 7q35. (B) Expression levels of TAS2R41 and TAS2R60 in human neutrophils were analyzed by using the RNA-seq database. [file Image_5.tif]

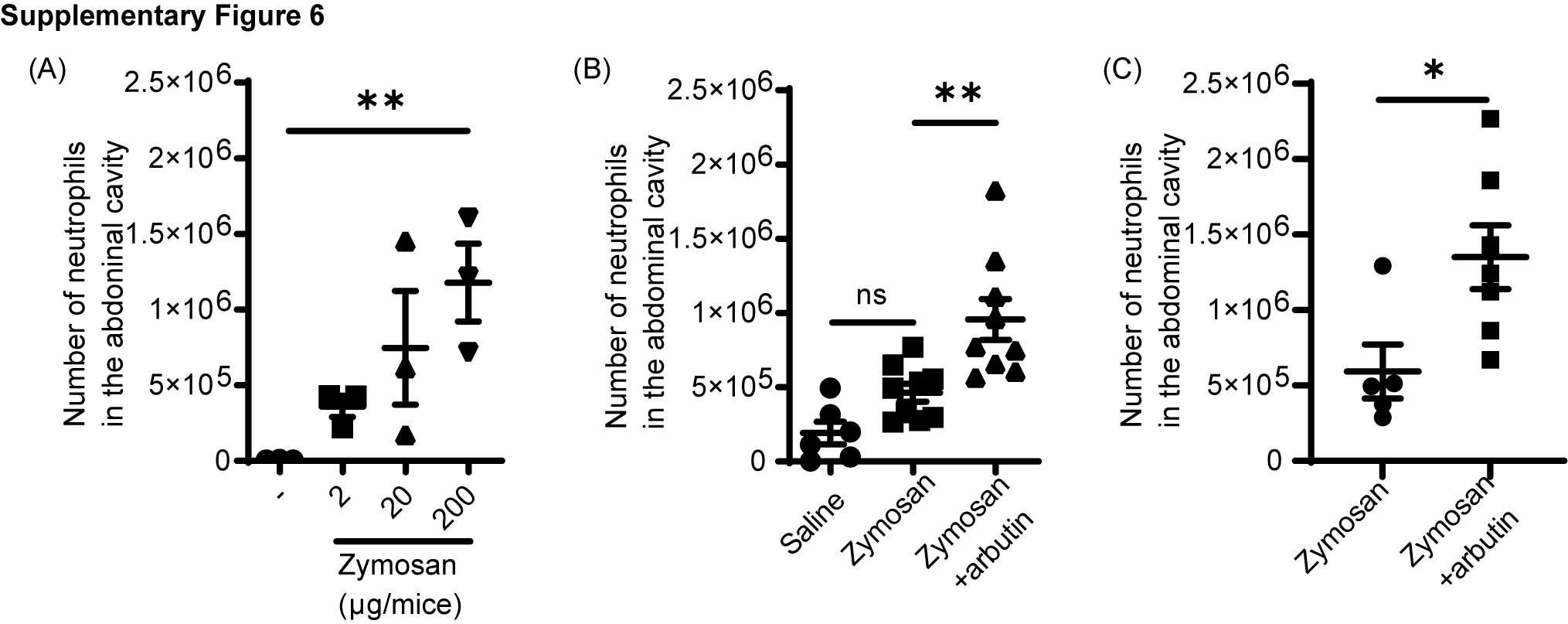

Supplement: Supplementary Figure 6 — Arbutin enhances neutrophil infiltration in a zymosan-induced peritonitis model in a Tas2R126/135/143-independent manner. (A) Wild-type C57BL/6 mice were injected IP with the indicated amount of zymosan in 0.5 ml sterile saline. Infiltrated neutrophils in peritoneal lavage fluid were collected 6 h later and enumerated by FACS analysis. Data represent the mean ± SEM of three mice. (B, C) Wild-type C57BL/6 mice (B) and Tas2r126/135/143 −/− mice (C) were injected IP with saline, 2 μg zymosan, or 2 μg zymosan with 25 mg arbutin, and infiltered neutrophils were enumerated 6 h later by FACS analysis. Data represent the mean ± SEM of 5–9 mice. *p < 0.05 by Student’s t test. **p < 0.05 by one-way ANOVA followed by Tukey’s multiple comparison test. [file Image_6.tif]
