# Supplementary material for: Tas2R signaling enhances mouse neutrophil migration via a ROCK-dependent pathway
Source: Front Immunol. 2022 Aug 18;13:973880. doi: 10.3389/fimmu.2022.973880 (PMC9436316; doi:10.3389/fimmu.2022.973880)
Supplement: Supplementary Table 1 — List of gRNA on-target sequences and PCR primer sequences. [file Table_1.pdf]

Supplementary Table 1

CRISP-Cas9	
gRNA for Tas2r	5′-AGTGTTTTTGATCCCAATAG-3′
gRNA for Tas2r	5′-TGGAAGTGAGCTCATCTACG-3′
Genotyping PCR	
Fw1	5′-AGGGGTCTTTCCAAGCACTC-3′
Rv1	5′-CCACCTGCTCTTTGAGGTTC-3′
Fw2	5′-CACACCTGGGGCTGCTTGGT-3′
Rv2	5′-TTGGCCAGGGCTCTCACTGC-3′
qPCR	
β-actin Fw	5′-CAGCTGAGAGGGAAATCGTG-3′
β-actin Rv	5′-TCTCCAGGGAGGAAGAGGAT-3′
Tas2r126 Fw	5′-GTGTGTGGGATTGGTCAACA-3′
Tas2r126 Rv	5′-GCTCCCGGAGTACTCAACC-3′
Tas2r135 Fw	5′-CCATCATGTCCACAGGAGAA-3′
Tas2r135 Rv	5′-TCAGTAGTCTGACATCCAAGAACTGT-3′
Tas2r143 Fw	5′-CAGGCATCTTTTTGAACTCCA-3′
Tas2r143 Rv	5′-TCTTCAGGGCCTTTCTCAGT-3′
