# Supplementary material for: Tas2R signaling enhances mouse neutrophil migration via a ROCK-dependent pathway
Source: Front Immunol. 2022 Aug 18;13:973880. doi: 10.3389/fimmu.2022.973880 (PMC9436316; doi:10.3389/fimmu.2022.973880)
Supplement: Supplementary Data Set 1 — Analysis of 36 mouse Tas2r gene expression levels in immune cells by ImmGen RNA-seq database. [file DataSheet_1.pdf]

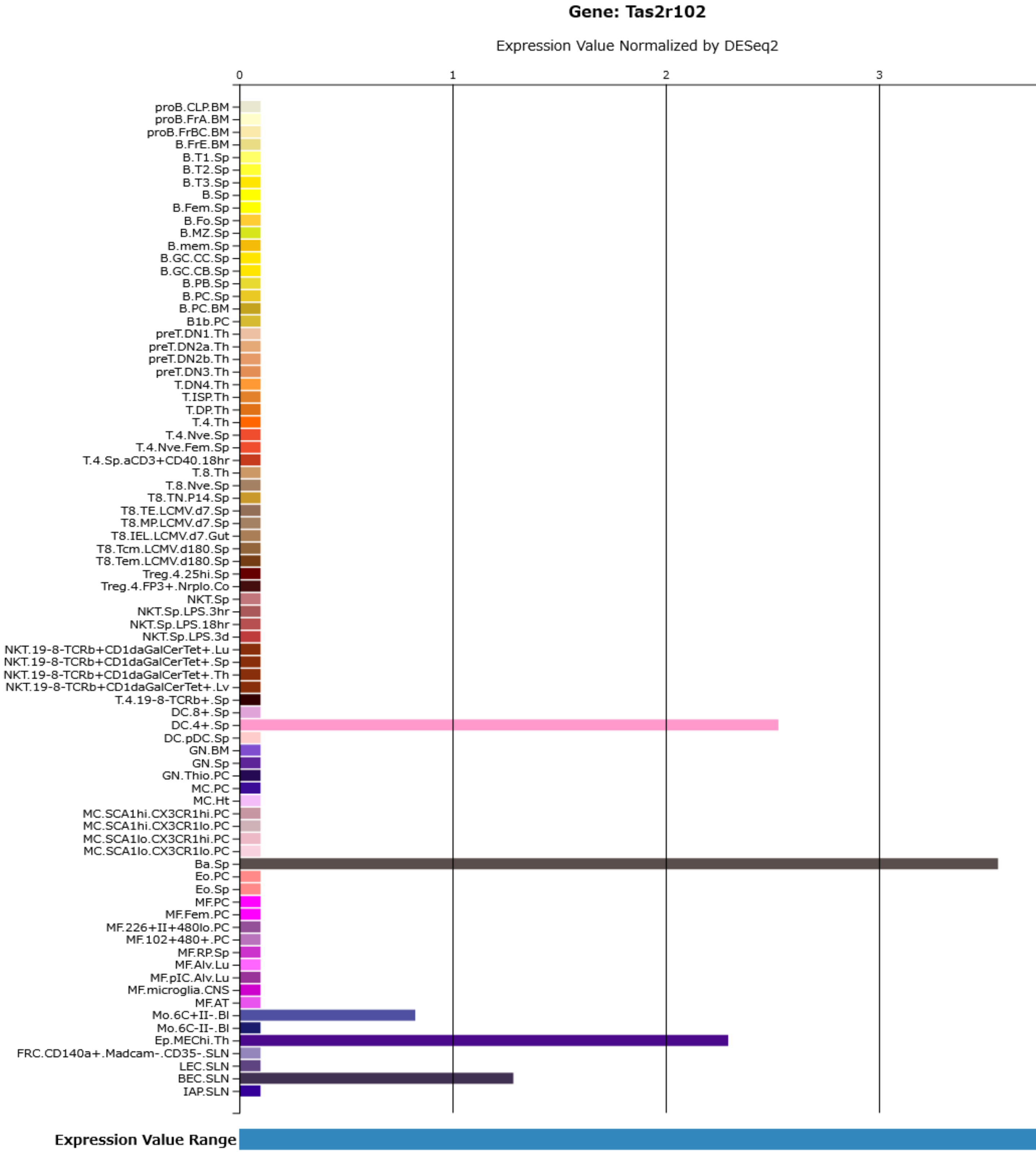

Gene: Tas2r102
Expression Value Normalized by DESeq2
0
1
2
3
proB.CLP.BM
proB.FrA.BM
proB.FrBC.BM
B.FrE.BM
B.T1.Sp
B.T2.Sp
B.T3.Sp
B.Sp
B.Fem.Sp
B.Fo.Sp
B.MZ.Sp
B.mem.Sp
B.GC.CC.Sp
B.GC.CB.Sp
B.PB.Sp
B.PC.Sp
B.PC.BM
B1b.PC
preT.DN1.Th
preT.DN2a.Th
preT.DN2b.Th
preT.DN3.Th
T.DN4.Th
T.ISP.Th
T.DP.Th
T.4.Th
T.4.Nve.Sp
T.4.Nve.Fem.Sp
T.4.Sp.aCD3+CD40.18hr
T.8.Th
T.8.Nve.Sp
T8.TN.P14.Sp
T8.TE.LCMV.d7.Sp
T8.MP.LCMV.d7.Sp
T8.IEL.LCMV.d7.Gut
T8.Tcm.LCMV.d180.Sp
T8.Tem.LCMV.d180.Sp
Treg.4.25hi.Sp
Treg.4.FP3+.Nrplo.Co
NKT.Sp
NKT.Sp.LPS.3hr
NKT.Sp.LPS.18hr
NKT.Sp.LPS.3d
NKT.19-8-TCRb+CD1daGalCerTet+.Lu
NKT.19-8-TCRb+CD1daGalCerTet+.Sp
NKT.19-8-TCRb+CD1daGalCerTet+.Th
NKT.19-8-TCRb+CD1daGalCerTet+.Lv
T.4.19-8-TCRb+.Sp
DC.8+.Sp
DC.4+.Sp
DC.pDC.Sp
GN.BM
GN.Sp
GN.Thio.PC
MC.PC
MC.Ht
MC.SCA1hi.CX3CR1hi.PC
MC.SCA1hi.CX3CR1lo.PC
MC.SCA1lo.CX3CR1hi.PC
MC.SCA1lo.CX3CR1lo.PC
Ba.Sp
Eo.PC
Eo.Sp
MF.PC
MF.Fem.PC
MF.226+II+480lo.PC
MF.102+480+.PC
MF.RP.Sp
MF.Alv.Lu
MF.pIC.Alv.Lu
MF.microglia.CNS
MF.AT
Mo.6C+II-.Bl
Mo.6C-II-.Bl
Ep.MEChi.Th
FRC.CD140a+.Madcam-.CD35-.SLN
LEC.SLN
BEC.SLN
IAP.SLN
Expression Value Range

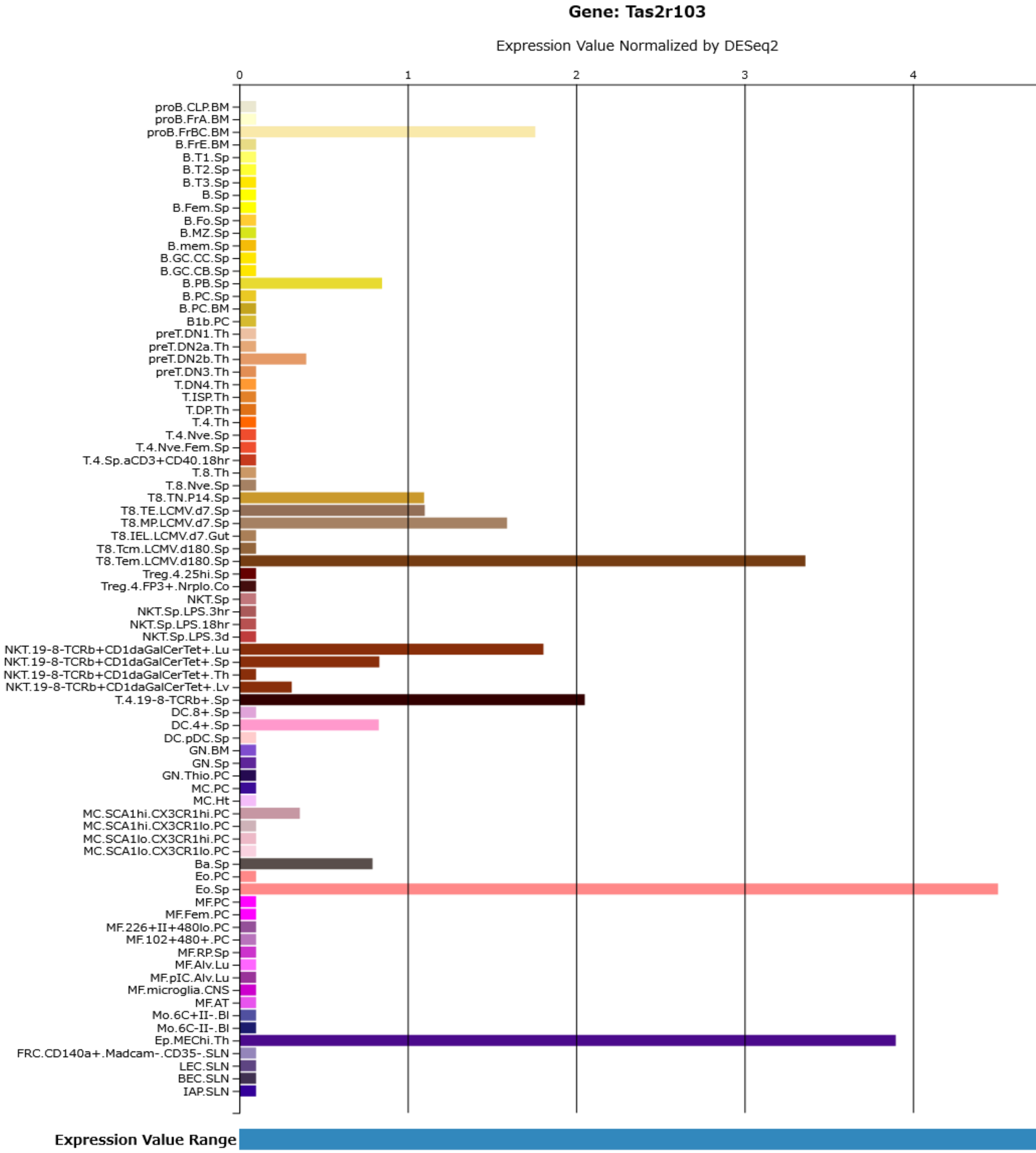
Gene: Tas2r103
Expression Value Normalized by DESeq2
0
1
2
3
4
proB.CLP.BM
proB.FrA.BM
proB.FrBC.BM
B.FrE.BM
B.T1.Sp
B.T2.Sp
B.T3.Sp
B.Sp
B.Fem.Sp
B.Fo.Sp
B.MZ.Sp
B.mem.Sp
B.GC.CC.Sp
B.GC.CB.Sp
B.PB.Sp
B.PC.Sp
B.PC.BM
B1b.PC
preT.DN1.Th
preT.DN2a.Th
preT.DN2b.Th
preT.DN3.Th
T.DN4.Th
T.ISP.Th
T.DP.Th
T.4.Th
T.4.Nve.Sp
T.4.Nve.Fem.Sp
T.4.Sp.aCD3+CD40.18hr
T.8.Th
T.8.Nve.Sp
T8.TN.P14.Sp
T8.TE.LCMV.d7.Sp
T8.MP.LCMV.d7.Sp
T8.IEL.LCMV.d7.Gut
T8.Tcm.LCMV.d180.Sp
T8.Tem.LCMV.d180.Sp
Treg.4.25hi.Sp
Treg.4.FP3+.Nrplo.Co
NKT.Sp
NKT.Sp.LPS.3hr
NKT.Sp.LPS.18hr
NKT.Sp.LPS.3d
NKT.19-8-TCRb+CD1daGalCerTet+.Lu
NKT.19-8-TCRb+CD1daGalCerTet+.Sp
NKT.19-8-TCRb+CD1daGalCerTet+.Th
NKT.19-8-TCRb+CD1daGalCerTet+.Lv
T.4.19-8-TCRb+.Sp
DC.8+.Sp
DC.4+.Sp
DC.pDC.Sp
GN.BM
GN.Sp
GN.Thio.PC
MC.PC
MC.Ht
MC.SCA1hi.CX3CR1hi.PC
MC.SCA1hi.CX3CR1lo.PC
MC.SCA1lo.CX3CR1hi.PC
MC.SCA1lo.CX3CR1lo.PC
Ba.Sp
Eo.PC
Eo.Sp
MF.PC
MF.Fem.PC
MF.226+II+480lo.PC
MF.102+480+.PC
MF.RP.Sp
MF.Alv.Lu
MF.pIC.Alv.Lu
MF.microglia.CNS
MF.AT
Mo.6C+II-.Bl
Mo.6C-II-.Bl
Ep.MEChi.Th
FRC.CD140a+.Madcam-.CD35-.SLN
LEC.SLN
BEC.SLN
IAP.SLN
Expression Value Range

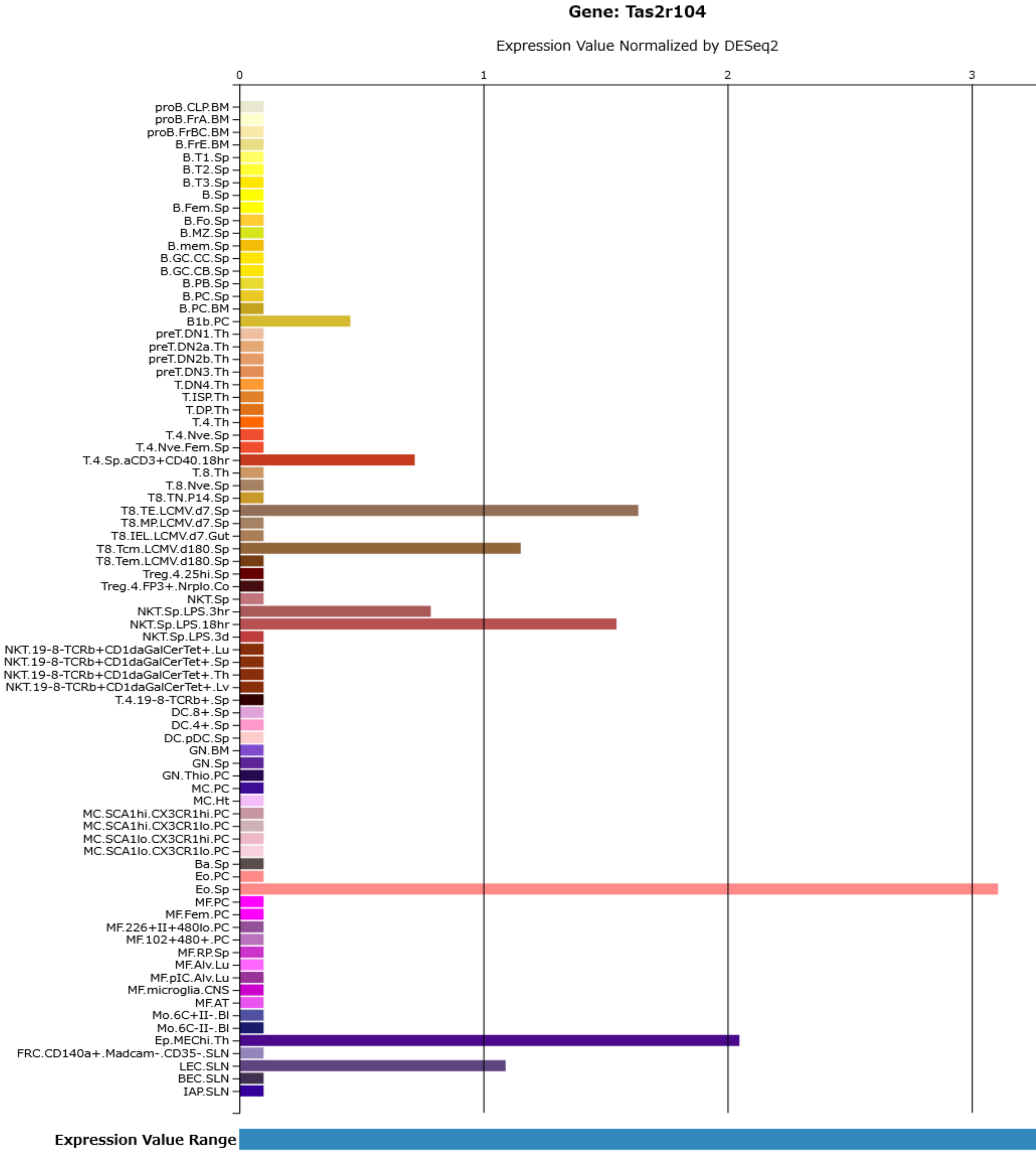
Gene: Tas2r104
Expression Value Normalized by DESeq2
0
1
2
3
proB.CLP.BM
proB.FrA.BM
proB.FrBC.BM
B.FrE.BM
B.T1.Sp
B.T2.Sp
B.T3.Sp
B.Sp
B.Fem.Sp
B.Fo.Sp
B.MZ.Sp
B.mem.Sp
B.GC.CC.Sp
B.GC.CB.Sp
B.PB.Sp
B.PC.Sp
B.PC.BM
B1b.PC
preT.DN1.Th
preT.DN2a.Th
preT.DN2b.Th
preT.DN3.Th
T.DN4.Th
T.ISP.Th
T.DP.Th
T.4.Th
T.4.Nve.Sp
T.4.Nve.Fem.Sp
T.4.Sp.aCD3+CD40.18hr
T.8.Th
T.8.Nve.Sp
T8.TN.P14.Sp
T8.TE.LCMV.d7.Sp
T8.MP.LCMV.d7.Sp
T8.IEL.LCMV.d7.Gut
T8.Tcm.LCMV.d180.Sp
T8.Tem.LCMV.d180.Sp
Treg.4.25hi.Sp
Treg.4.FP3+.Nrplo.Co
NKT.Sp
NKT.Sp.LPS.3hr
NKT.Sp.LPS.18hr
NKT.Sp.LPS.3d
NKT.19-8-TCRb+CD1daGalCerTet+.Lu
NKT.19-8-TCRb+CD1daGalCerTet+.Sp
NKT.19-8-TCRb+CD1daGalCerTet+.Th
NKT.19-8-TCRb+CD1daGalCerTet+.Lv
T.4.19-8-TCRb+.Sp
DC.8+.Sp
DC.4+.Sp
DC.pDC.Sp
GN.BM
GN.Sp
GN.Thio.PC
MC.PC
MC.Ht
MC.SCA1hi.CX3CR1hi.PC
MC.SCA1hi.CX3CR1lo.PC
MC.SCA1lo.CX3CR1hi.PC
MC.SCA1lo.CX3CR1lo.PC
Ba.Sp
Eo.PC
Eo.Sp
MF.PC
MF.Fem.PC
MF.226+II+480lo.PC
MF.102+480+.PC
MF.RP.Sp
MF.Alv.Lu
MF.pIC.Alv.Lu
MF.microglia.CNS
MF.AT
Mo.6C+II-.Bl
Mo.6C-II-.Bl
Ep.MEChi.Th
FRC.CD140a+.Madcam-.CD35-.SLN
LEC.SLN
BEC.SLN
IAP.SLN
Expression Value Range

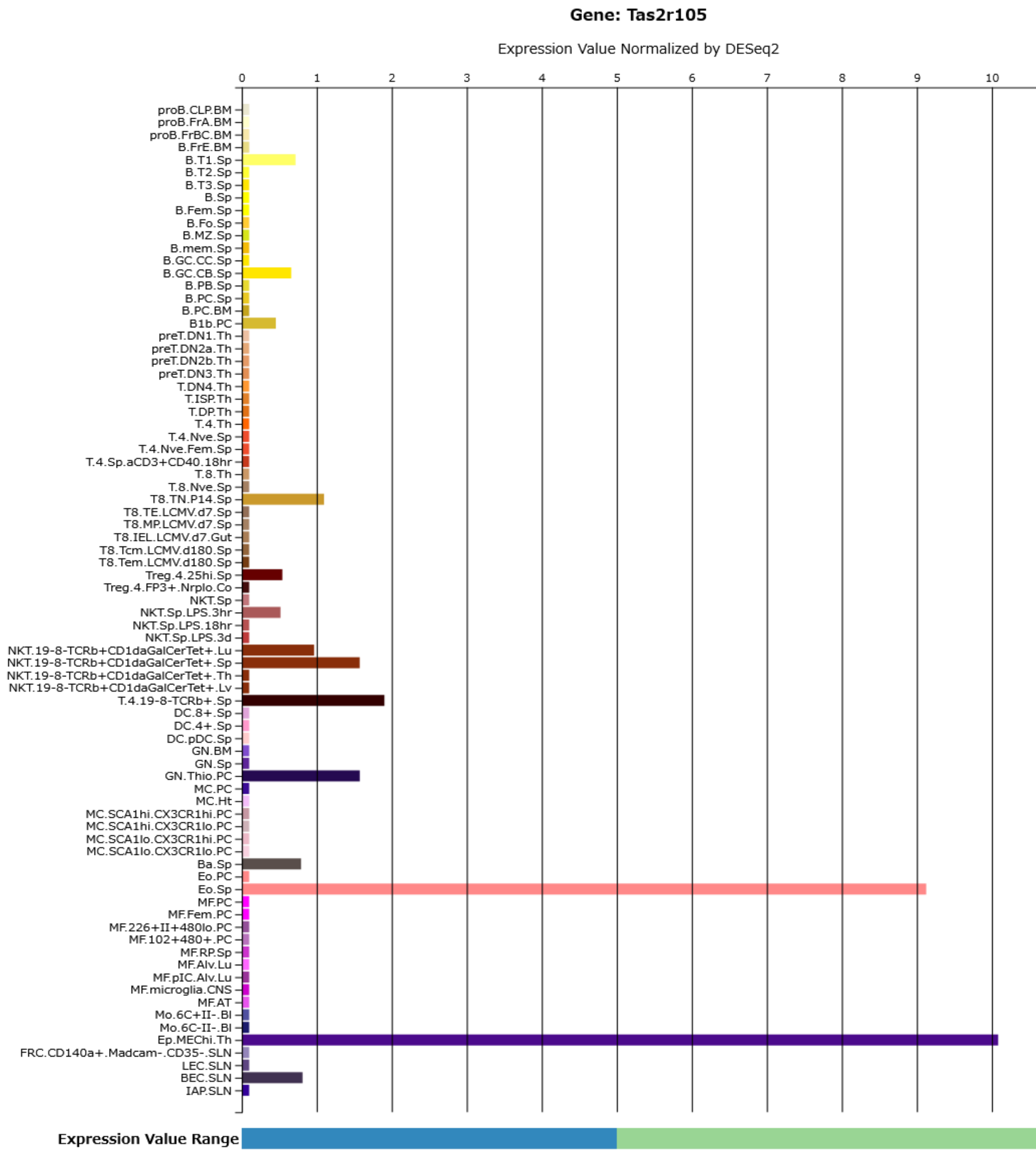

Gene: Tas2r105
Expression Value Normalized by DESeq2
0
1
2
3
4
5
6
7
8
9
10
proB.CLP.BM
proB.FrA.BM
proB.FrBC.BM
B.FrE.BM
B.T1.Sp
B.T2.Sp
B.T3.Sp
B.Sp
B.Fem.Sp
B.Fo.Sp
B.MZ.Sp
B.mem.Sp
B.GC.CC.Sp
B.GC.CB.Sp
B.PB.Sp
B.PC.Sp
B.PC.BM
B1b.PC
preT.DN1.Th
preT.DN2a.Th
preT.DN2b.Th
preT.DN3.Th
T.DN4.Th
T.ISP.Th
T.DP.Th
T.4.Th
T.4.Nve.Sp
T.4.Nve.Fem.Sp
T.4.Sp.aCD3+CD40.18hr
T.8.Th
T.8.Nve.Sp
T8.TN.P14.Sp
T8.TE.LCMV.d7.Sp
T8.MP.LCMV.d7.Sp
T8.IEL.LCMV.d7.Gut
T8.Tcm.LCMV.d180.Sp
T8.Tem.LCMV.d180.Sp
Treg.4.25hi.Sp
Treg.4.FP3+.Nrplo.Co
NKT.Sp
NKT.Sp.LPS.3hr
NKT.Sp.LPS.18hr
NKT.Sp.LPS.3d
NKT.19-8-TCRb+CD1daGalCerTet+.Lu
NKT.19-8-TCRb+CD1daGalCerTet+.Sp
NKT.19-8-TCRb+CD1daGalCerTet+.Th
NKT.19-8-TCRb+CD1daGalCerTet+.Lv
T.4.19-8-TCRb+.Sp
DC.8+.Sp
DC.4+.Sp
DC.pDC.Sp
GN.BM
GN.Sp
GN.Thio.PC
MC.PC
MC.Ht
MC.SCA1hi.CX3CR1hi.PC
MC.SCA1hi.CX3CR1lo.PC
MC.SCA1lo.CX3CR1hi.PC
MC.SCA1lo.CX3CR1lo.PC
Ba.Sp
Eo.PC
Eo.Sp
MF.PC
MF.Fem.PC
MF.226+II+480lo.PC
MF.102+480+.PC
MF.RP.Sp
MF.Alv.Lu
MF.pIC.Alv.Lu
MF.microglia.CNS
MF.AT
Mo.6C+II-.Bl
Mo.6C-II-.Bl
Ep.MEChi.Th
FRC.CD140a+.Madcam-.CD35-.SLN
LEC.SLN
BEC.SLN
IAP.SLN
Expression Value Range

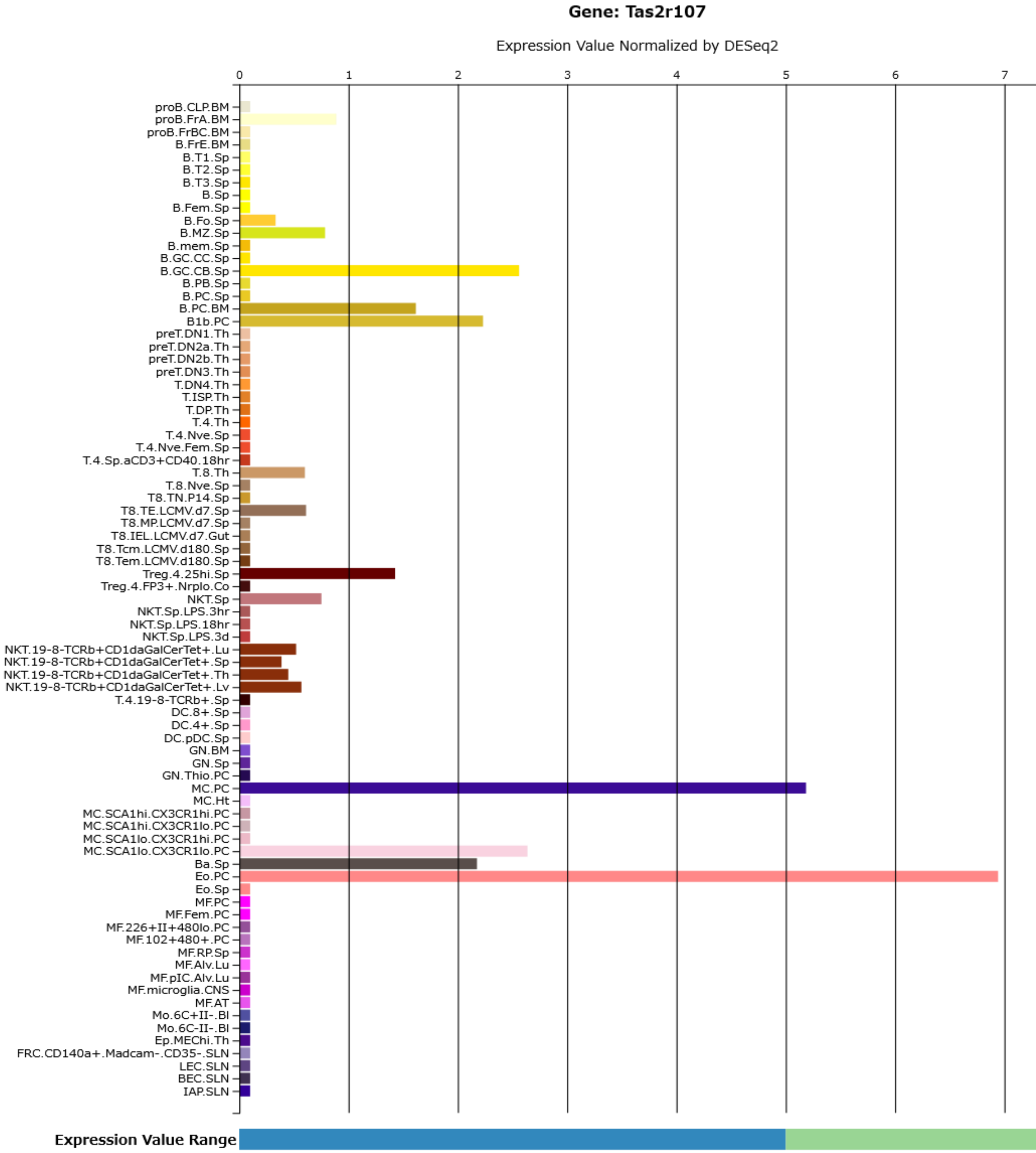
Gene: Tas2r107
Expression Value Normalized by DESeq2
0
1
2
3
4
5
6
7
proB.CLP.BM
proB.FrA.BM
proB.FrBC.BM
B.FrE.BM
B.T1.Sp
B.T2.Sp
B.T3.Sp
B.Sp
B.Fem.Sp
B.Fo.Sp
B.MZ.Sp
B.mem.Sp
B.GC.CC.Sp
B.GC.CB.Sp
B.PB.Sp
B.PC.Sp
B.PC.BM
B1b.PC
preT.DN1.Th
preT.DN2a.Th
preT.DN2b.Th
preT.DN3.Th
T.DN4.Th
T.ISP.Th
T.DP.Th
T.4.Th
T.4.Nve.Sp
T.4.Nve.Fem.Sp
T.4.Sp.aCD3+CD40.18hr
T.8.Th
T.8.Nve.Sp
T8.TN.P14.Sp
T8.TE.LCMV.d7.Sp
T8.MP.LCMV.d7.Sp
T8.IEL.LCMV.d7.Gut
T8.Tcm.LCMV.d180.Sp
T8.Tem.LCMV.d180.Sp
Treg.4.25hi.Sp
Treg.4.FP3+.Nrplo.Co
NKT.Sp
NKT.Sp.LPS.3hr
NKT.Sp.LPS.18hr
NKT.Sp.LPS.3d
NKT.19-8-TCRb+CD1daGalCerTet+.Lu
NKT.19-8-TCRb+CD1daGalCerTet+.Sp
NKT.19-8-TCRb+CD1daGalCerTet+.Th
NKT.19-8-TCRb+CD1daGalCerTet+.Lv
T.4.19-8-TCRb+.Sp
DC.8+.Sp
DC.4+.Sp
DC.pDC.Sp
GN.BM
GN.Sp
GN.Thio.PC
MC.PC
MC.Ht
MC.SCA1hi.CX3CR1hi.PC
MC.SCA1hi.CX3CR1lo.PC
MC.SCA1lo.CX3CR1hi.PC
MC.SCA1lo.CX3CR1lo.PC
Ba.Sp
Eo.PC
Eo.Sp
MF.PC
MF.Fem.PC
MF.226+II+480lo.PC
MF.102+480+.PC
MF.RP.Sp
MF.Alv.Lu
MF.pIC.Alv.Lu
MF.microglia.CNS
MF.AT
Mo.6C+II-.Bl
Mo.6C-II-.Bl
Ep.MEChi.Th
FRC.CD140a+.Madcam-.CD35-.SLN
LEC.SLN
BEC.SLN
IAP.SLN
Expression Value Range

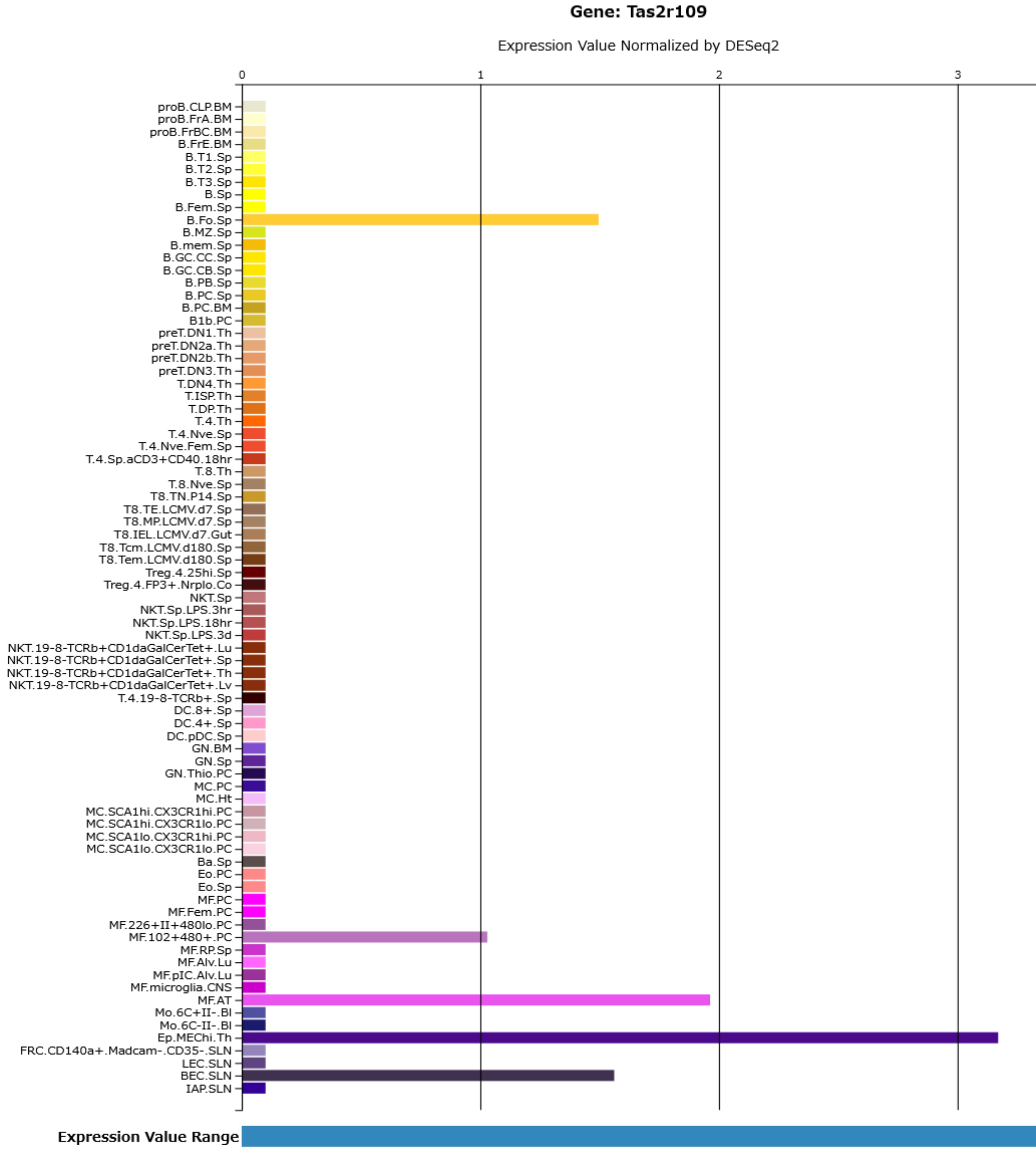

Gene: Tas2r109
Expression Value Normalized by DESeq2
0
1
2
3
proB.CLP.BM
proB.FrA.BM
proB.FrBC.BM
B.FrE.BM
B.T1.Sp
B.T2.Sp
B.T3.Sp
B.Sp
B.Fem.Sp
B.Fo.Sp
B.MZ.Sp
B.mem.Sp
B.GC.CC.Sp
B.GC.CB.Sp
B.PB.Sp
B.PC.Sp
B.PC.BM
B1b.PC
preT.DN1.Th
preT.DN2a.Th
preT.DN2b.Th
preT.DN3.Th
T.DN4.Th
T.ISP.Th
T.DP.Th
T.4.Th
T.4.Nve.Sp
T.4.Nve.Fem.Sp
T.4.Sp.aCD3+CD40.18hr
T.8.Th
T.8.Nve.Sp
T8.TN.P14.Sp
T8.TE.LCMV.d7.Sp
T8.MP.LCMV.d7.Sp
T8.IEL.LCMV.d7.Gut
T8.Tcm.LCMV.d180.Sp
T8.Tem.LCMV.d180.Sp
Treg.4.25hi.Sp
Treg.4.FP3+.Nrplo.Co
NKT.Sp
NKT.Sp.LPS.3hr
NKT.Sp.LPS.18hr
NKT.Sp.LPS.3d
NKT.19-8-TCRb+CD1daGalCerTet+.Lu
NKT.19-8-TCRb+CD1daGalCerTet+.Sp
NKT.19-8-TCRb+CD1daGalCerTet+.Th
NKT.19-8-TCRb+CD1daGalCerTet+.Lv
T.4.19-8-TCRb+.Sp
DC.8+.Sp
DC.4+.Sp
DC.pDC.Sp
GN.BM
GN.Sp
GN.Thio.PC
MC.PC
MC.Ht
MC.SCA1hi.CX3CR1hi.PC
MC.SCA1hi.CX3CR1lo.PC
MC.SCA1lo.CX3CR1hi.PC
MC.SCA1lo.CX3CR1lo.PC
Ba.Sp
Eo.PC
Eo.Sp
MF.PC
MF.Fem.PC
MF.226+II+480lo.PC
MF.102+480+.PC
MF.RP.Sp
MF.Alv.Lu
MF.pIC.Alv.Lu
MF.microglia.CNS
MF.AT
Mo.6C+II-.Bl
Mo.6C-II-.Bl
Ep.MEChi.Th
FRC.CD140a+.Madcam-.CD35-.SLN
LEC.SLN
BEC.SLN
IAP.SLN
Expression Value Range

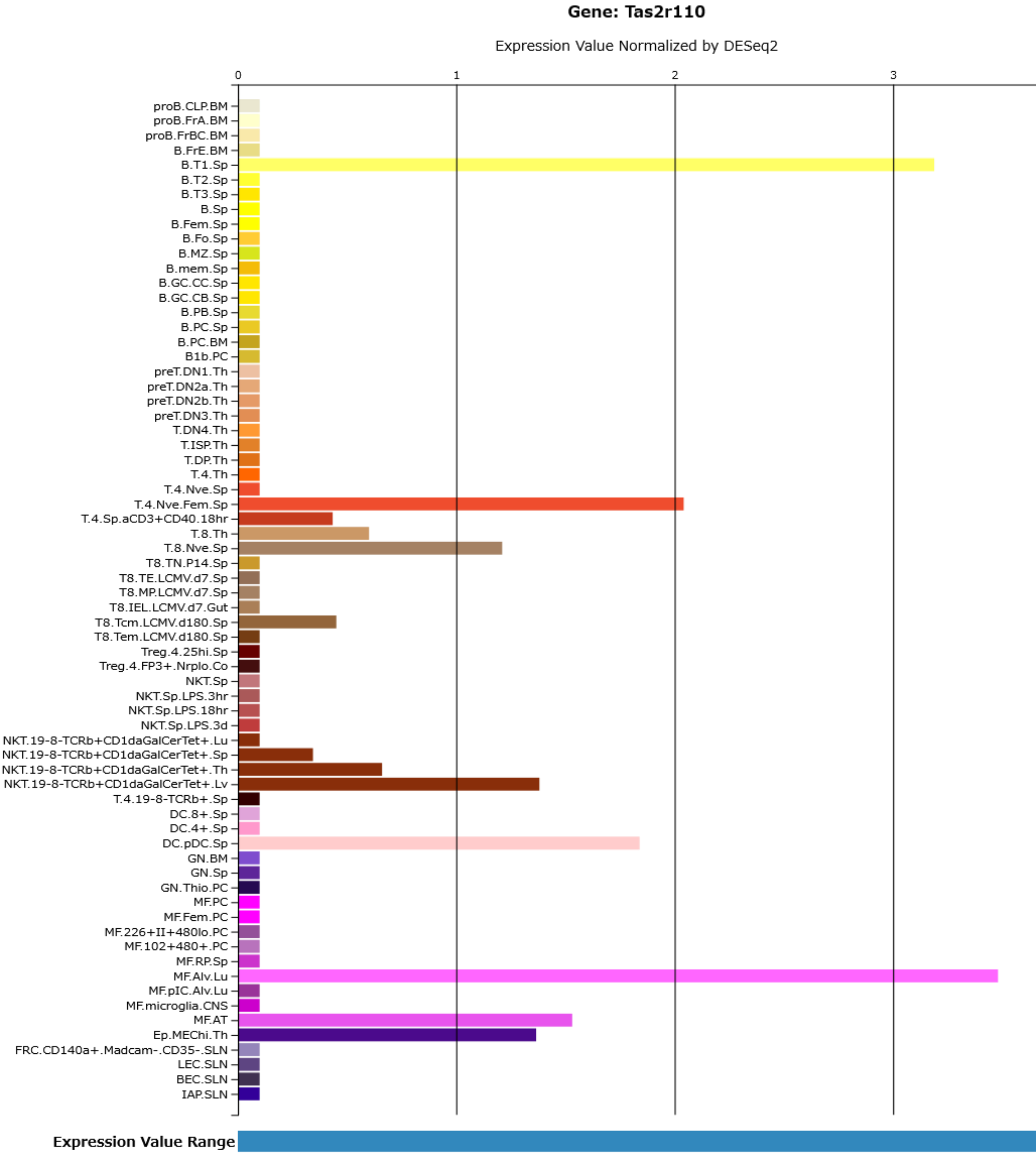
Gene: Tas2r110
Expression Value Normalized by DESeq2
0
1
2
3
proB.CLP.BM
proB.FrA.BM
proB.FrBC.BM
B.FrE.BM
B.T1.Sp
B.T2.Sp
B.T3.Sp
B.Sp
B.Fem.Sp
B.Fo.Sp
B.MZ.Sp
B.mem.Sp
B.GC.CC.Sp
B.GC.CB.Sp
B.PB.Sp
B.PC.Sp
B.PC.BM
B1b.PC
preT.DN1.Th
preT.DN2a.Th
preT.DN2b.Th
preT.DN3.Th
T.DN4.Th
T.ISP.Th
T.DP.Th
T.4.Th
T.4.Nve.Sp
T.4.Nve.Fem.Sp
T.4.Sp.aCD3+CD40.18hr
T.8.Th
T.8.Nve.Sp
T8.TN.P14.Sp
T8.TE.LCMV.d7.Sp
T8.MP.LCMV.d7.Sp
T8.IEL.LCMV.d7.Gut
T8.Tcm.LCMV.d180.Sp
T8.Tem.LCMV.d180.Sp
Treg.4.25hi.Sp
Treg.4.FP3+.Nrplo.Co
NKT.Sp
NKT.Sp.LPS.3hr
NKT.Sp.LPS.18hr
NKT.Sp.LPS.3d
NKT.19-8-TCRb+CD1daGalCerTet+.Lu
NKT.19-8-TCRb+CD1daGalCerTet+.Sp
NKT.19-8-TCRb+CD1daGalCerTet+.Th
NKT.19-8-TCRb+CD1daGalCerTet+.Lv
T.4.19-8-TCRb+.Sp
DC.8+.Sp
DC.4+.Sp
DC.pDC.Sp
GN.BM
GN.Sp
GN.Thio.PC
MF.PC
MF.Fem.PC
MF.226+II+480lo.PC
MF.102+480+.PC
MF.RP.Sp
MF.Alv.Lu
MF.pIC.Alv.Lu
MF.microglia.CNS
MF.AT
Ep.MEChi.Th
FRC.CD140a+.Madcam-.CD35-.SLN
LEC.SLN
BEC.SLN
IAP.SLN
Expression Value Range

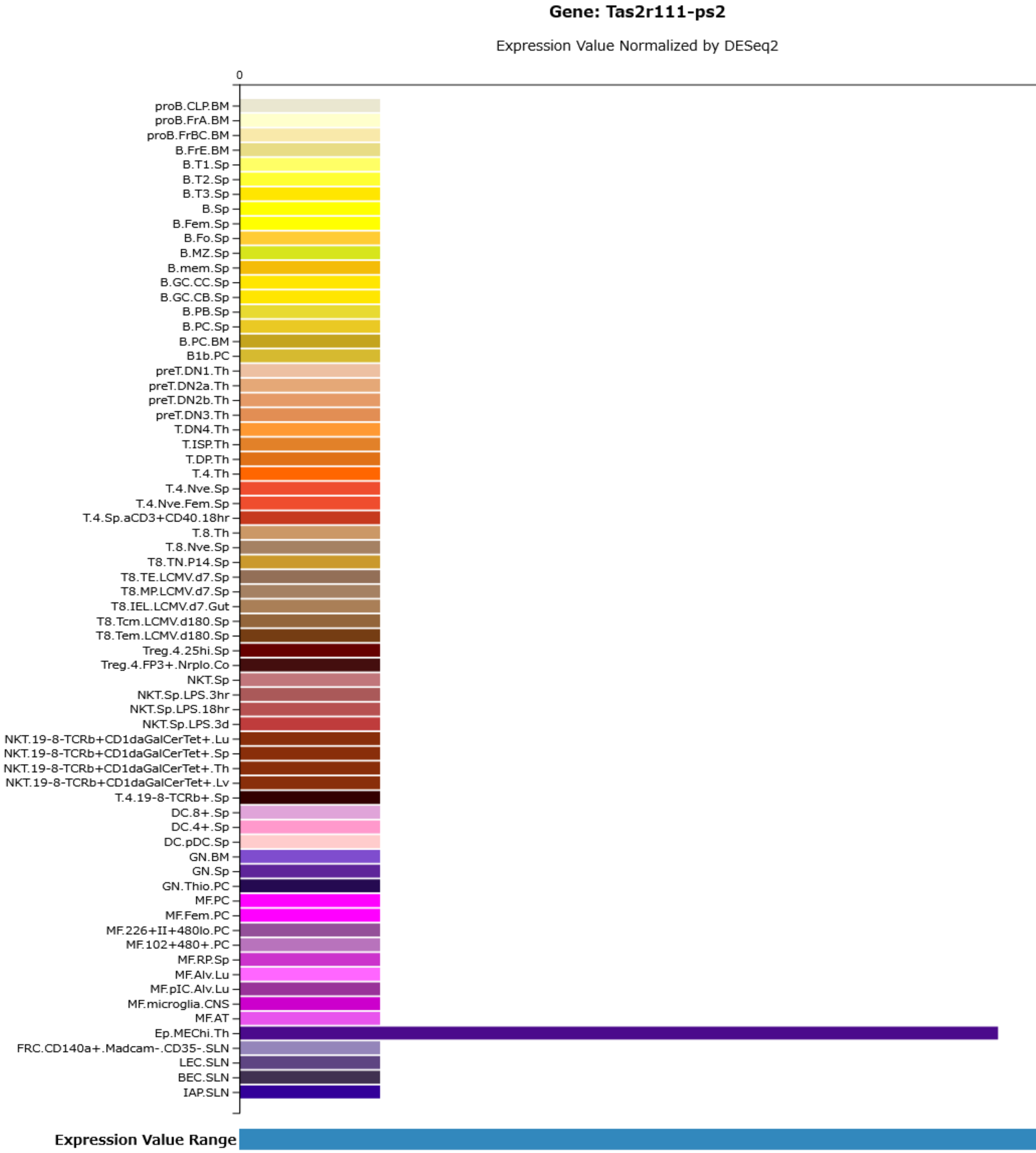
Gene: Tas2r111-ps2
Expression Value Normalized by DESeq2
0
proB.CLP.BM
proB.FrA.BM
proB.FrBC.BM
B.FrE.BM
B.T1.Sp
B.T2.Sp
B.T3.Sp
B.Sp
B.Fem.Sp
B.Fo.Sp
B.MZ.Sp
B.mem.Sp
B.GC.CC.Sp
B.GC.CB.Sp
B.PB.Sp
B.PC.Sp
B.PC.BM
B1b.PC
preT.DN1.Th
preT.DN2a.Th
preT.DN2b.Th
preT.DN3.Th
T.DN4.Th
T.ISP.Th
T.DP.Th
T.4.Th
T.4.Nve.Sp
T.4.Nve.Fem.Sp
T.4.Sp.aCD3+CD40.18hr
T.8.Th
T.8.Nve.Sp
T8.TN.P14.Sp
T8.TE.LCMV.d7.Sp
T8.MP.LCMV.d7.Sp
T8.IEL.LCMV.d7.Gut
T8.Tcm.LCMV.d180.Sp
T8.Tem.LCMV.d180.Sp
Treg.4.25hi.Sp
Treg.4.FP3+.Nrplo.Co
NKT.Sp
NKT.Sp.LPS.3hr
NKT.Sp.LPS.18hr
NKT.Sp.LPS.3d
NKT.19-8-TCRb+CD1daGalCerTet+.Lu
NKT.19-8-TCRb+CD1daGalCerTet+.Sp
NKT.19-8-TCRb+CD1daGalCerTet+.Th
NKT.19-8-TCRb+CD1daGalCerTet+.Lv
T.4.19-8-TCRb+.Sp
DC.8+.Sp
DC.4+.Sp
DC.pDC.Sp
GN.BM
GN.Sp
GN.Thio.PC
MF.PC
MF.Fem.PC
MF.226+II+480lo.PC
MF.102+480+.PC
MF.RP.Sp
MF.Alv.Lu
MF.pIC.Alv.Lu
MF.microglia.CNS
MF.AT
Ep.MEChi.Th
FRC.CD140a+.Madcam-.CD35-.SLN
LEC.SLN
BEC.SLN
IAP.SLN
Expression Value Range

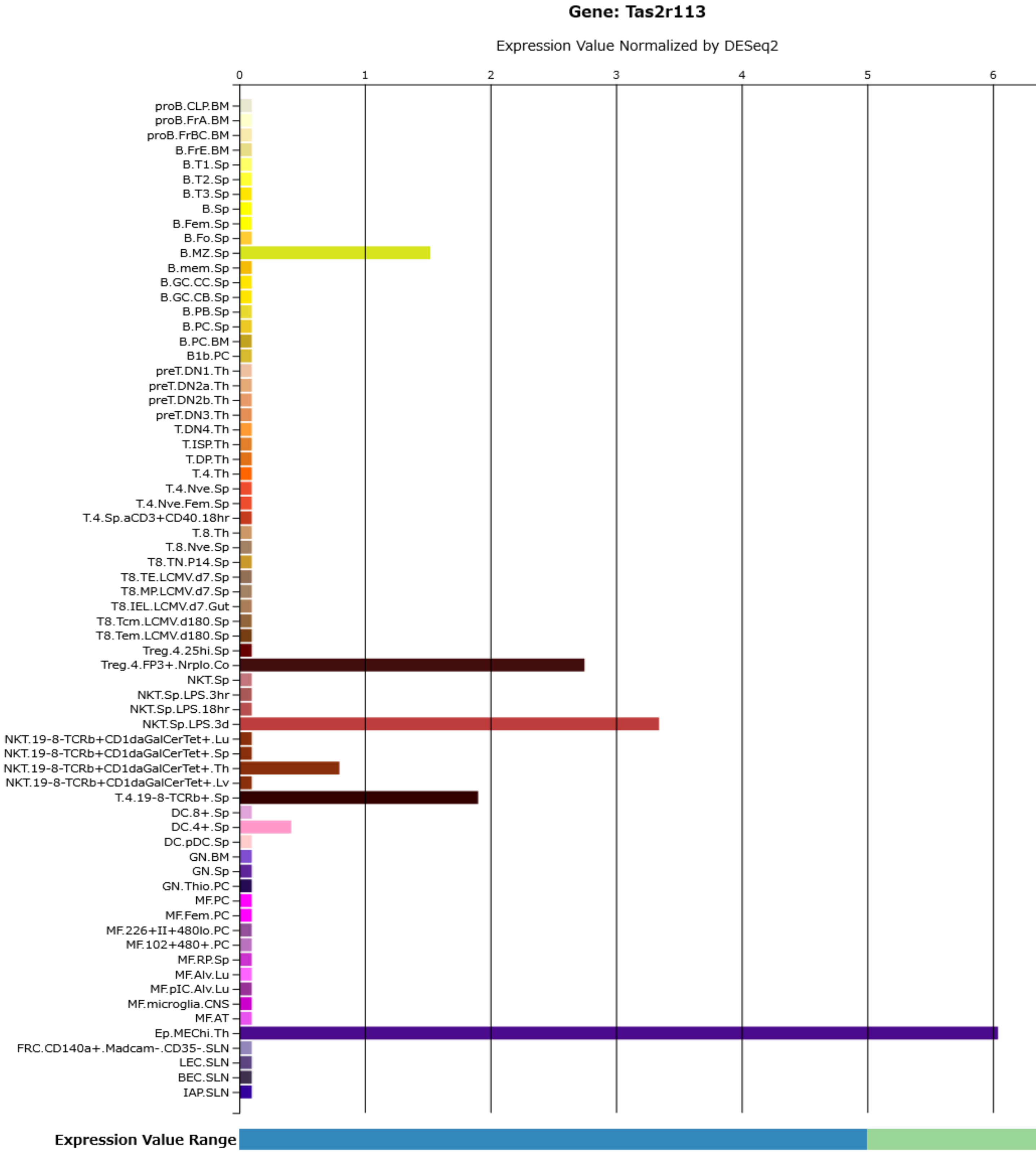
Gene: Tas2r113
Expression Value Normalized by DESeq2
0
1
2
3
4
5
6
proB.CLP.BM
proB.FrA.BM
proB.FrBC.BM
B.FrE.BM
B.T1.Sp
B.T2.Sp
B.T3.Sp
B.Sp
B.Fem.Sp
B.Fo.Sp
B.MZ.Sp
B.mem.Sp
B.GC.CC.Sp
B.GC.CB.Sp
B.PB.Sp
B.PC.Sp
B.PC.BM
B1b.PC
preT.DN1.Th
preT.DN2a.Th
preT.DN2b.Th
preT.DN3.Th
T.DN4.Th
T.ISP.Th
T.DP.Th
T.4.Th
T.4.Nve.Sp
T.4.Nve.Fem.Sp
T.4.Sp.aCD3+CD40.18hr
T.8.Th
T.8.Nve.Sp
T8.TN.P14.Sp
T8.TE.LCMV.d7.Sp
T8.MP.LCMV.d7.Sp
T8.IEL.LCMV.d7.Gut
T8.Tcm.LCMV.d180.Sp
T8.Tem.LCMV.d180.Sp
Treg.4.25hi.Sp
Treg.4.FP3+.Nrplo.Co
NKT.Sp
NKT.Sp.LPS.3hr
NKT.Sp.LPS.18hr
NKT.Sp.LPS.3d
NKT.19-8-TCRb+CD1daGalCerTet+.Lu
NKT.19-8-TCRb+CD1daGalCerTet+.Sp
NKT.19-8-TCRb+CD1daGalCerTet+.Th
NKT.19-8-TCRb+CD1daGalCerTet+.Lv
T.4.19-8-TCRb+.Sp
DC.8+.Sp
DC.4+.Sp
DC.pDC.Sp
GN.BM
GN.Sp
GN.Thio.PC
MF.PC
MF.Fem.PC
MF.226+II+480lo.PC
MF.102+480+.PC
MF.RP.Sp
MF.Alv.Lu
MF.pIC.Alv.Lu
MF.microglia.CNS
MF.AT
Ep.MEChi.Th
FRC.CD140a+.Madcam-.CD35-.SLN
LEC.SLN
BEC.SLN
IAP.SLN
Expression Value Range

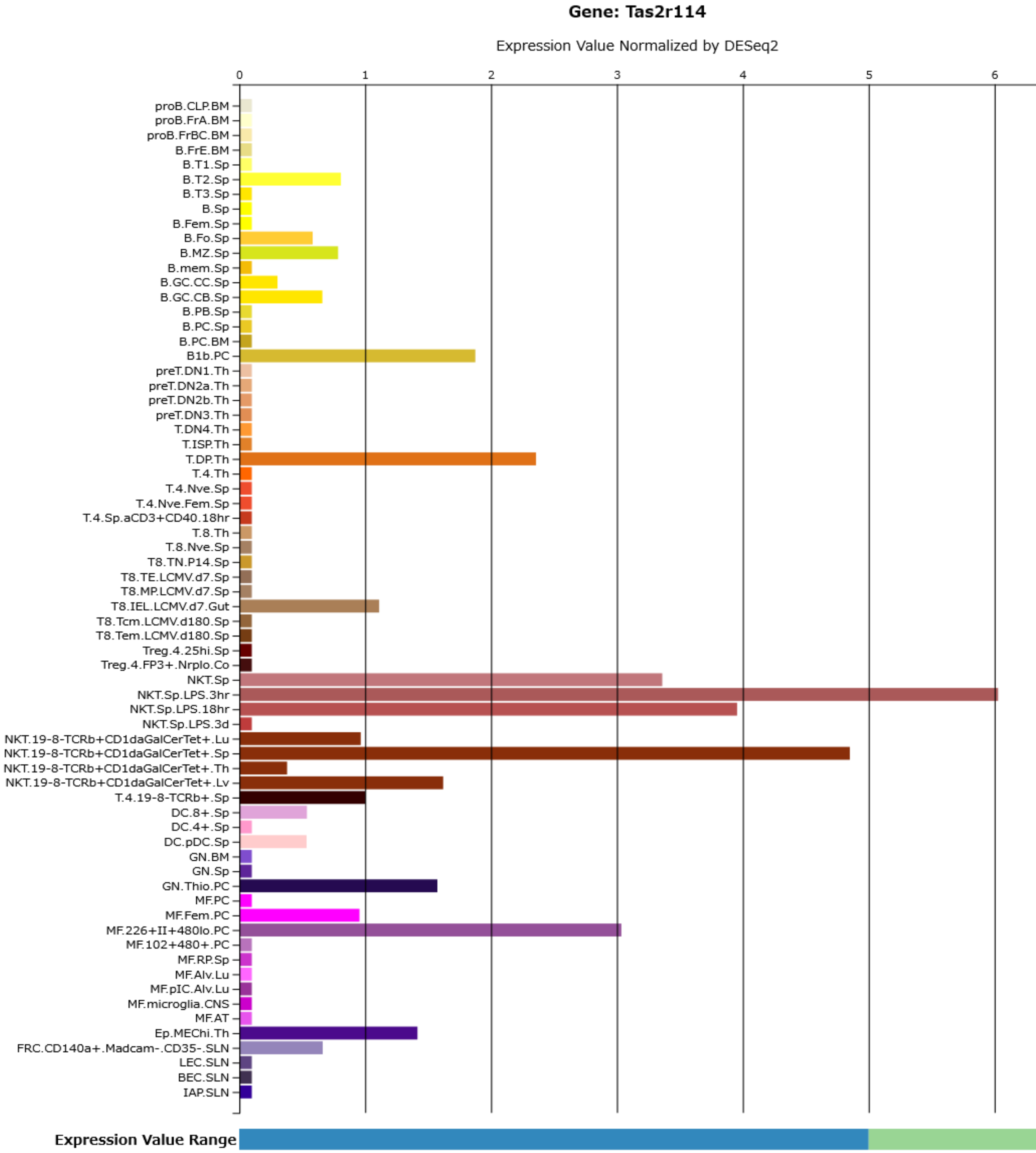

Gene: Tas2r114
Expression Value Normalized by DESeq2
0
1
2
3
4
5
6
proB.CLP.BM
proB.FrA.BM
proB.FrBC.BM
B.FrE.BM
B.T1.Sp
B.T2.Sp
B.T3.Sp
B.Sp
B.Fem.Sp
B.Fo.Sp
B.MZ.Sp
B.mem.Sp
B.GC.CC.Sp
B.GC.CB.Sp
B.PB.Sp
B.PC.Sp
B.PC.BM
B1b.PC
preT.DN1.Th
preT.DN2a.Th
preT.DN2b.Th
preT.DN3.Th
T.DN4.Th
T.ISP.Th
T.DP.Th
T.4.Th
T.4.Nve.Sp
T.4.Nve.Fem.Sp
T.4.Sp.aCD3+CD40.18hr
T.8.Th
T.8.Nve.Sp
T8.TN.P14.Sp
T8.TE.LCMV.d7.Sp
T8.MP.LCMV.d7.Sp
T8.IEL.LCMV.d7.Gut
T8.Tcm.LCMV.d180.Sp
T8.Tem.LCMV.d180.Sp
Treg.4.25hi.Sp
Treg.4.FP3+.Nrplo.Co
NKT.Sp
NKT.Sp.LPS.3hr
NKT.Sp.LPS.18hr
NKT.Sp.LPS.3d
NKT.19-8-TCRb+CD1daGalCerTet+.Lu
NKT.19-8-TCRb+CD1daGalCerTet+.Sp
NKT.19-8-TCRb+CD1daGalCerTet+.Th
NKT.19-8-TCRb+CD1daGalCerTet+.Lv
T.4.19-8-TCRb+.Sp
DC.8+.Sp
DC.4+.Sp
DC.pDC.Sp
GN.BM
GN.Sp
GN.Thio.PC
MF.PC
MF.Fem.PC
MF.226+II+480lo.PC
MF.102+480+.PC
MF.RP.Sp
MF.Alv.Lu
MF.pIC.Alv.Lu
MF.microglia.CNS
MF.AT
Ep.MEChi.Th
FRC.CD140a+.Madcam-.CD35-.SLN
LEC.SLN
BEC.SLN
IAP.SLN
Expression Value Range

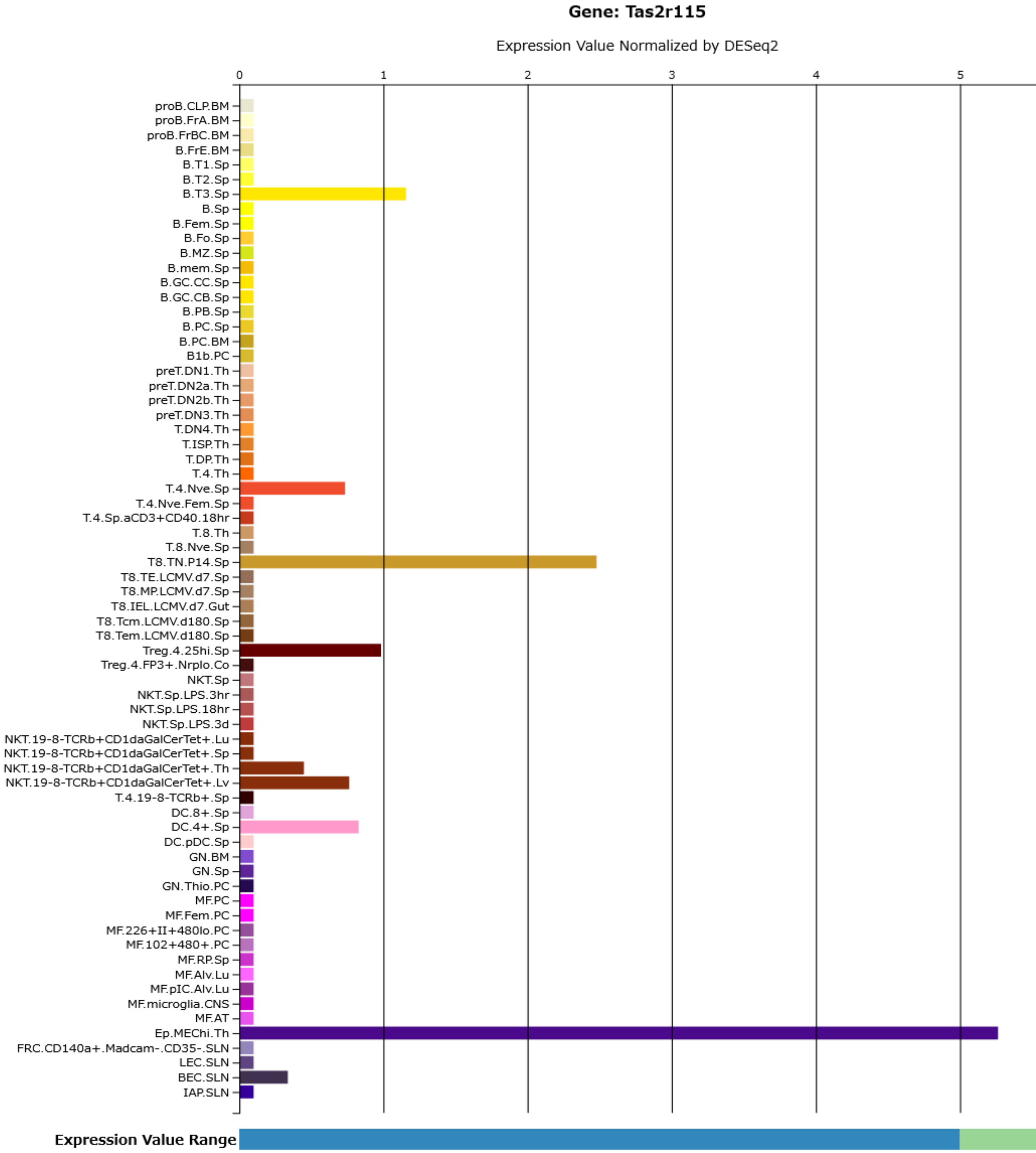

Gene: Tas2r115
Expression Value Normalized by DESeq2
0
1
2
3
4
5
proB.CLP.BM
proB.FrA.BM
proB.FrBC.BM
B.FrE.BM
B.T1.Sp
B.T2.Sp
B.T3.Sp
B.Sp
B.Fem.Sp
B.Fo.Sp
B.MZ.Sp
B.mem.Sp
B.GC.CC.Sp
B.GC.CB.Sp
B.PB.Sp
B.PC.Sp
B.PC.BM
B1b.PC
preT.DN1.Th
preT.DN2a.Th
preT.DN2b.Th
preT.DN3.Th
T.DN4.Th
T.ISP.Th
T.DP.Th
T.4.Th
T.4.Nve.Sp
T.4.Nve.Fem.Sp
T.4.Sp.aCD3+CD40.18hr
T.8.Th
T.8.Nve.Sp
T8.TN.P14.Sp
T8.TE.LCMV.d7.Sp
T8.MP.LCMV.d7.Sp
T8.IEL.LCMV.d7.Gut
T8.Tcm.LCMV.d180.Sp
T8.Tem.LCMV.d180.Sp
Treg.4.25hi.Sp
Treg.4.FP3+.Nrplo.Co
NKT.Sp
NKT.Sp.LPS.3hr
NKT.Sp.LPS.18hr
NKT.Sp.LPS.3d
NKT.19-8-TCRb+CD1daGalCerTet+.Lu
NKT.19-8-TCRb+CD1daGalCerTet+.Sp
NKT.19-8-TCRb+CD1daGalCerTet+.Th
NKT.19-8-TCRb+CD1daGalCerTet+.Lv
T.4.19-8-TCRb+.Sp
DC.8+.Sp
DC.4+.Sp
DC.pDC.Sp
GN.BM
GN.Sp
GN.Thio.PC
MF.PC
MF.Fem.PC
MF.226+II+480lo.PC
MF.102+480+.PC
MF.RP.Sp
MF.Alv.Lu
MF.pIC.Alv.Lu
MF.microglia.CNS
MF.AT
Ep.MEChi.Th
FRC.CD140a+.Madcam-.CD35-.SLN
LEC.SLN
BEC.SLN
IAP.SLN
Expression Value Range

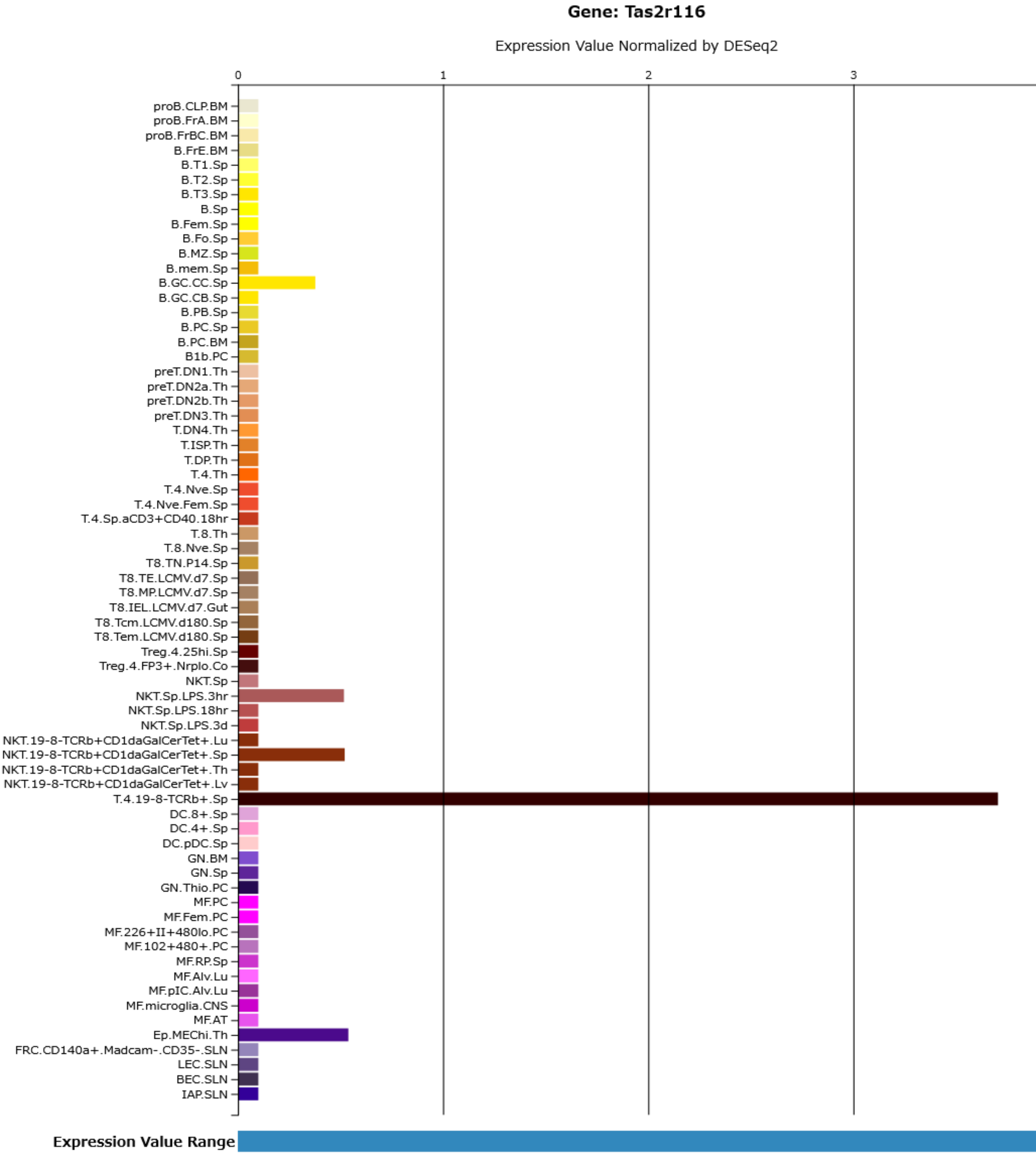
Gene: Tas2r116
Expression Value Normalized by DESeq2
0
1
2
3
proB.CLP.BM
proB.FrA.BM
proB.FrBC.BM
B.FrE.BM
B.T1.Sp
B.T2.Sp
B.T3.Sp
B.Sp
B.Fem.Sp
B.Fo.Sp
B.MZ.Sp
B.mem.Sp
B.GC.CC.Sp
B.GC.CB.Sp
B.PB.Sp
B.PC.Sp
B.PC.BM
B1b.PC
preT.DN1.Th
preT.DN2a.Th
preT.DN2b.Th
preT.DN3.Th
T.DN4.Th
T.ISP.Th
T.DP.Th
T.4.Th
T.4.Nve.Sp
T.4.Nve.Fem.Sp
T.4.Sp.aCD3+CD40.18hr
T.8.Th
T.8.Nve.Sp
T8.TN.P14.Sp
T8.TE.LCMV.d7.Sp
T8.MP.LCMV.d7.Sp
T8.IEL.LCMV.d7.Gut
T8.Tcm.LCMV.d180.Sp
T8.Tem.LCMV.d180.Sp
Treg.4.25hi.Sp
Treg.4.FP3+.Nrplo.Co
NKT.Sp
NKT.Sp.LPS.3hr
NKT.Sp.LPS.18hr
NKT.Sp.LPS.3d
NKT.19-8-TCRb+CD1daGalCerTet+.Lu
NKT.19-8-TCRb+CD1daGalCerTet+.Sp
NKT.19-8-TCRb+CD1daGalCerTet+.Th
NKT.19-8-TCRb+CD1daGalCerTet+.Lv
T.4.19-8-TCRb+.Sp
DC.8+.Sp
DC.4+.Sp
DC.pDC.Sp
GN.BM
GN.Sp
GN.Thio.PC
MF.PC
MF.Fem.PC
MF.226+II+480lo.PC
MF.102+480+.PC
MF.RP.Sp
MF.Alv.Lu
MF.pIC.Alv.Lu
MF.microglia.CNS
MF.AT
Ep.MEChi.Th
FRC.CD140a+.Madcam-.CD35-.SLN
LEC.SLN
BEC.SLN
IAP.SLN
Expression Value Range

Gene: Tas2r117

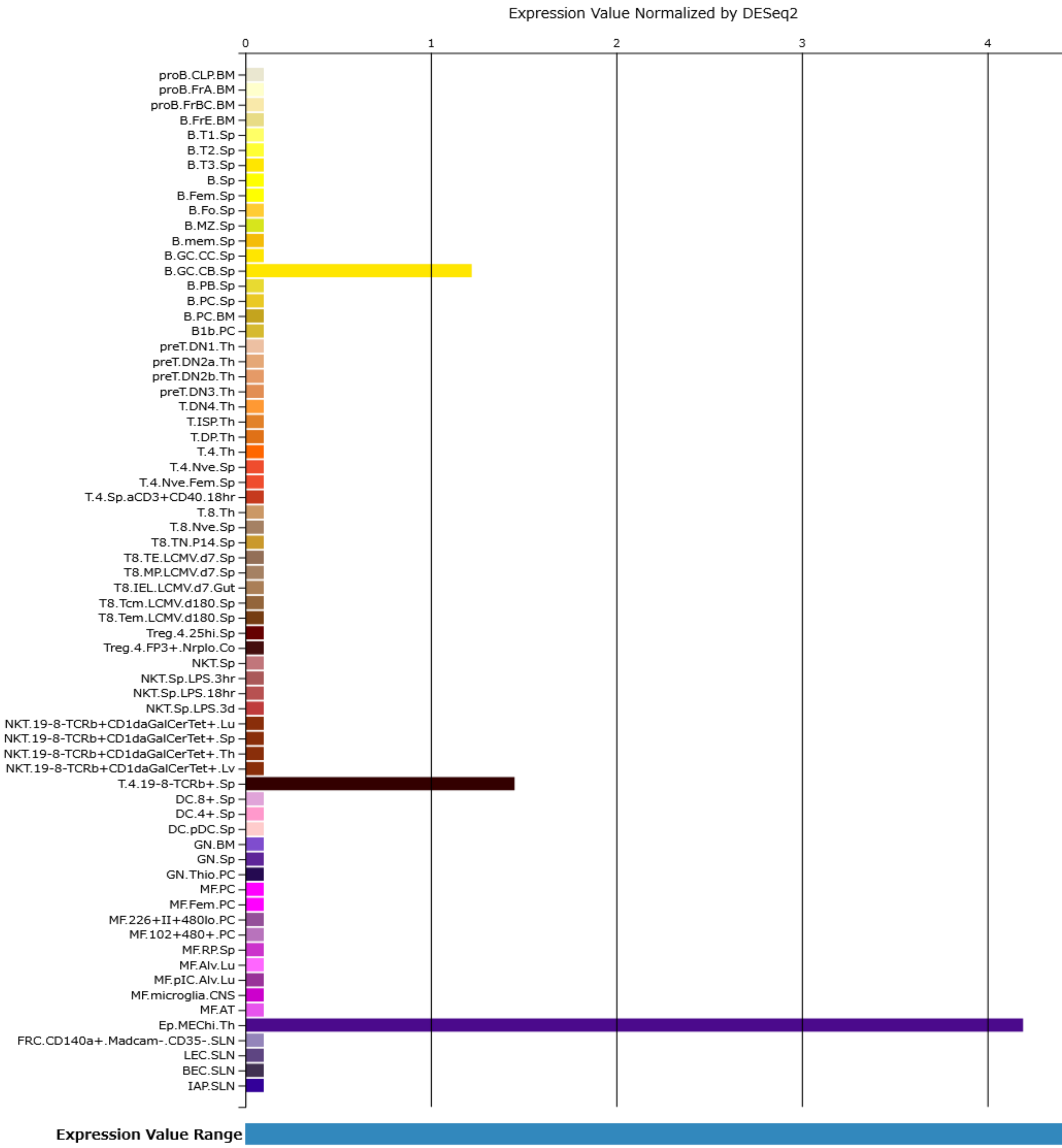

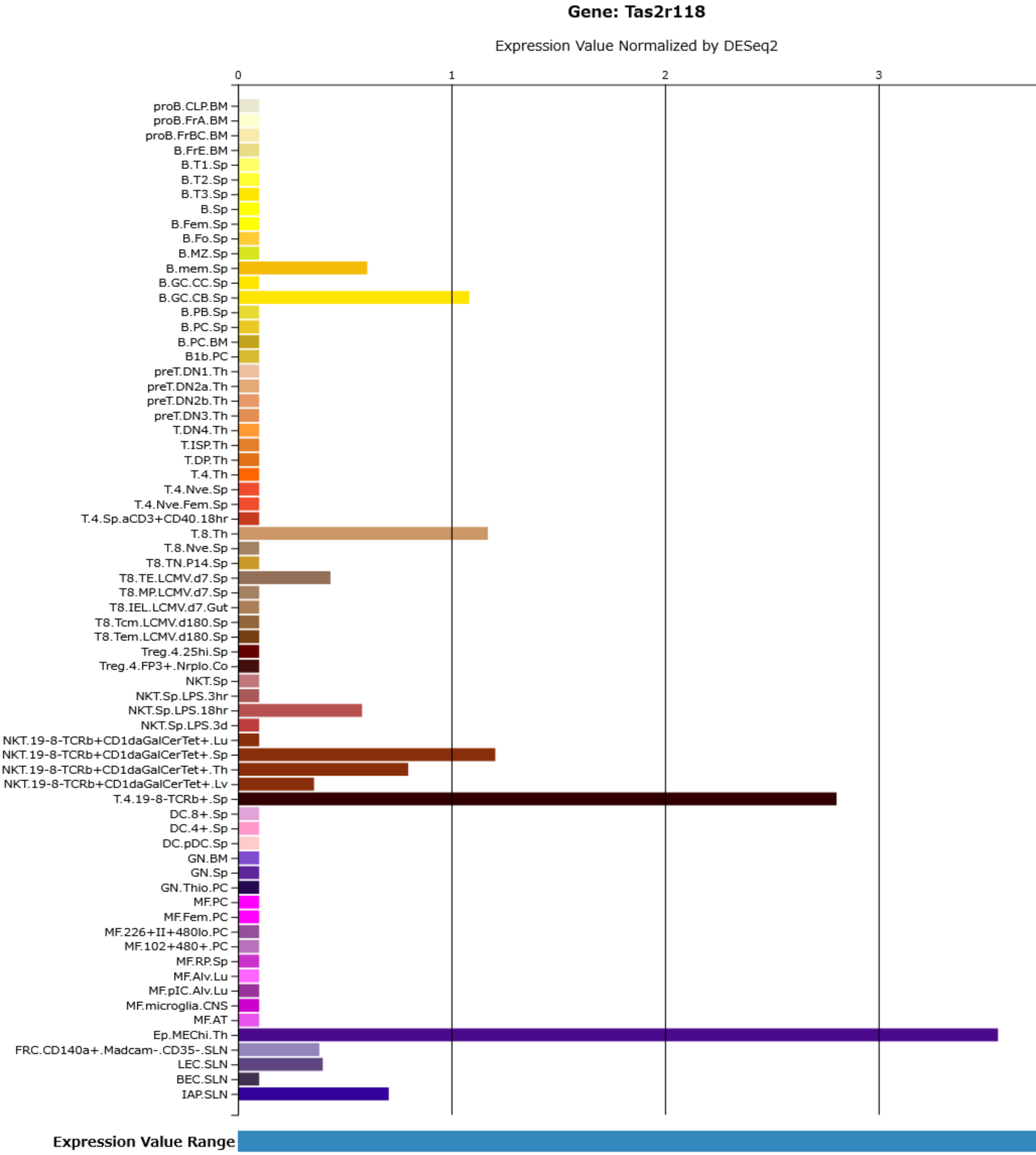

Gene: Tas2r118
Expression Value Normalized by DESeq2
0
1
2
3
proB.CLP.BM
proB.FrA.BM
proB.FrBC.BM
B.FrE.BM
B.T1.Sp
B.T2.Sp
B.T3.Sp
B.Sp
B.Fem.Sp
B.Fo.Sp
B.MZ.Sp
B.mem.Sp
B.GC.CC.Sp
B.GC.CB.Sp
B.PB.Sp
B.PC.Sp
B.PC.BM
B1b.PC
preT.DN1.Th
preT.DN2a.Th
preT.DN2b.Th
preT.DN3.Th
T.DN4.Th
T.ISP.Th
T.DP.Th
T.4.Th
T.4.Nve.Sp
T.4.Nve.Fem.Sp
T.4.Sp.aCD3+CD40.18hr
T.8.Th
T.8.Nve.Sp
T8.TN.P14.Sp
T8.TE.LCMV.d7.Sp
T8.MP.LCMV.d7.Sp
T8.IEL.LCMV.d7.Gut
T8.Tcm.LCMV.d180.Sp
T8.Tem.LCMV.d180.Sp
Treg.4.25hi.Sp
Treg.4.FP3+.Nrplo.Co
NKT.Sp
NKT.Sp.LPS.3hr
NKT.Sp.LPS.18hr
NKT.Sp.LPS.3d
NKT.19-8-TCRb+CD1daGalCerTet+.Lu
NKT.19-8-TCRb+CD1daGalCerTet+.Sp
NKT.19-8-TCRb+CD1daGalCerTet+.Th
NKT.19-8-TCRb+CD1daGalCerTet+.Lv
T.4.19-8-TCRb+.Sp
DC.8+.Sp
DC.4+.Sp
DC.pDC.Sp
GN.BM
GN.Sp
GN.Thio.PC
MF.PC
MF.Fem.PC
MF.226+II+480lo.PC
MF.102+480+.PC
MF.RP.Sp
MF.Alv.Lu
MF.pIC.Alv.Lu
MF.microglia.CNS
MF.AT
Ep.MEChi.Th
FRC.CD140a+.Madcam-.CD35-.SLN
LEC.SLN
BEC.SLN
IAP.SLN
Expression Value Range

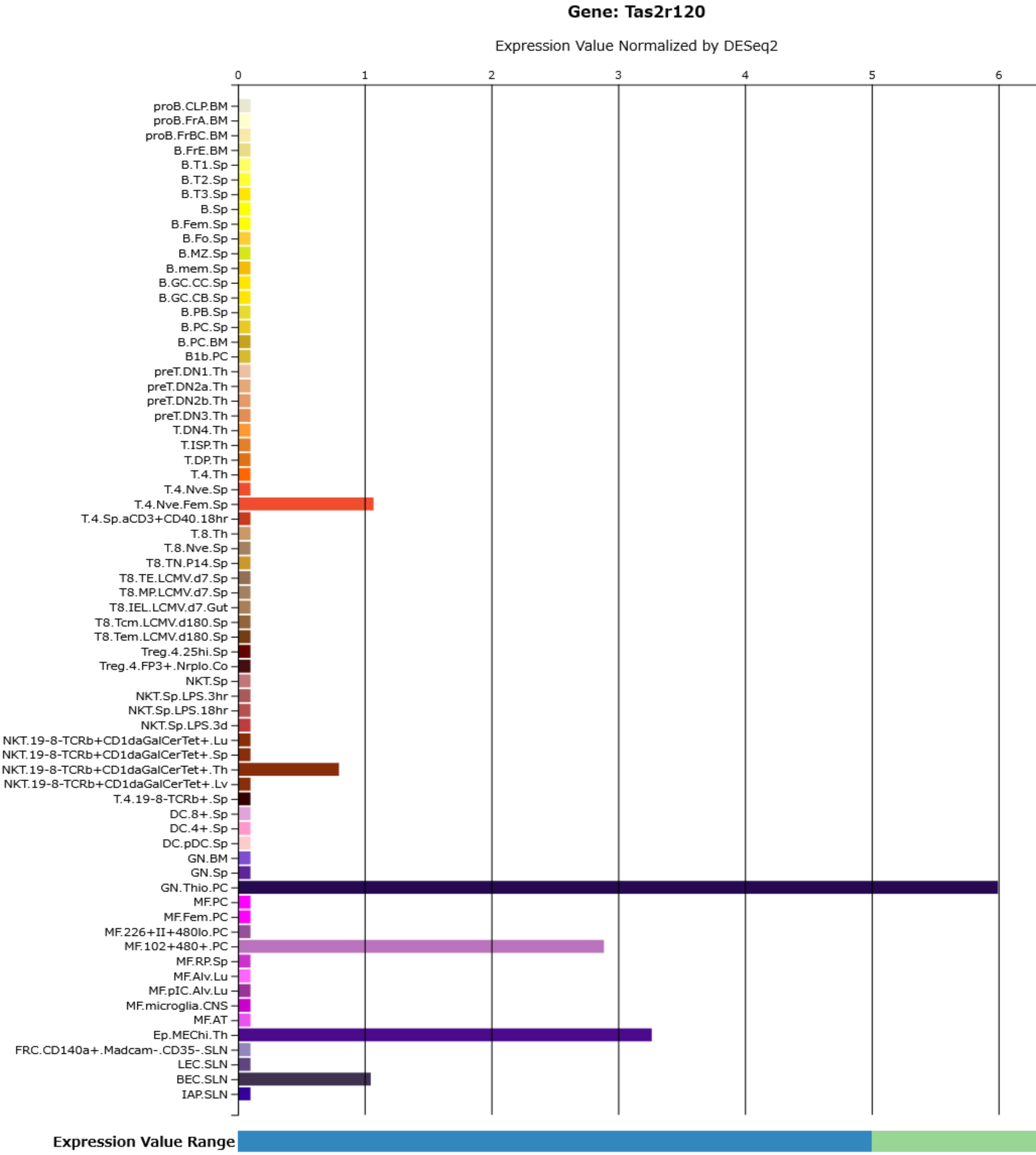

Gene: Tas2r120
Expression Value Normalized by DESeq2
0
1
2
3
4
5
6
proB.CLP.BM
proB.FrA.BM
proB.FrBC.BM
B.FrE.BM
B.T1.Sp
B.T2.Sp
B.T3.Sp
B.Sp
B.Fem.Sp
B.Fo.Sp
B.MZ.Sp
B.mem.Sp
B.GC.CC.Sp
B.GC.CB.Sp
B.PB.Sp
B.PC.Sp
B.PC.BM
B1b.PC
preT.DN1.Th
preT.DN2a.Th
preT.DN2b.Th
preT.DN3.Th
T.DN4.Th
T.ISP.Th
T.DP.Th
T.4.Th
T.4.Nve.Sp
T.4.Nve.Fem.Sp
T.4.Sp.aCD3+CD40.18hr
T.8.Th
T.8.Nve.Sp
T8.TN.P14.Sp
T8.TE.LCMV.d7.Sp
T8.MP.LCMV.d7.Sp
T8.IEL.LCMV.d7.Gut
T8.Tcm.LCMV.d180.Sp
T8.Tem.LCMV.d180.Sp
Treg.4.25hi.Sp
Treg.4.FP3+.Nrplo.Co
NKT.Sp
NKT.Sp.LPS.3hr
NKT.Sp.LPS.18hr
NKT.Sp.LPS.3d
NKT.19-8-TCRb+CD1daGalCerTet+.Lu
NKT.19-8-TCRb+CD1daGalCerTet+.Sp
NKT.19-8-TCRb+CD1daGalCerTet+.Th
NKT.19-8-TCRb+CD1daGalCerTet+.Lv
T.4.19-8-TCRb+.Sp
DC.8+.Sp
DC.4+.Sp
DC.pDC.Sp
GN.BM
GN.Sp
GN.Thio.PC
MF.PC
MF.Fem.PC
MF.226+II+480lo.PC
MF.102+480+.PC
MF.RP.Sp
MF.Alv.Lu
MF.pIC.Alv.Lu
MF.microglia.CNS
MF.AT
Ep.MEChi.Th
FRC.CD140a+.Madcam-.CD35-.SLN
LEC.SLN
BEC.SLN
IAP.SLN
Expression Value Range

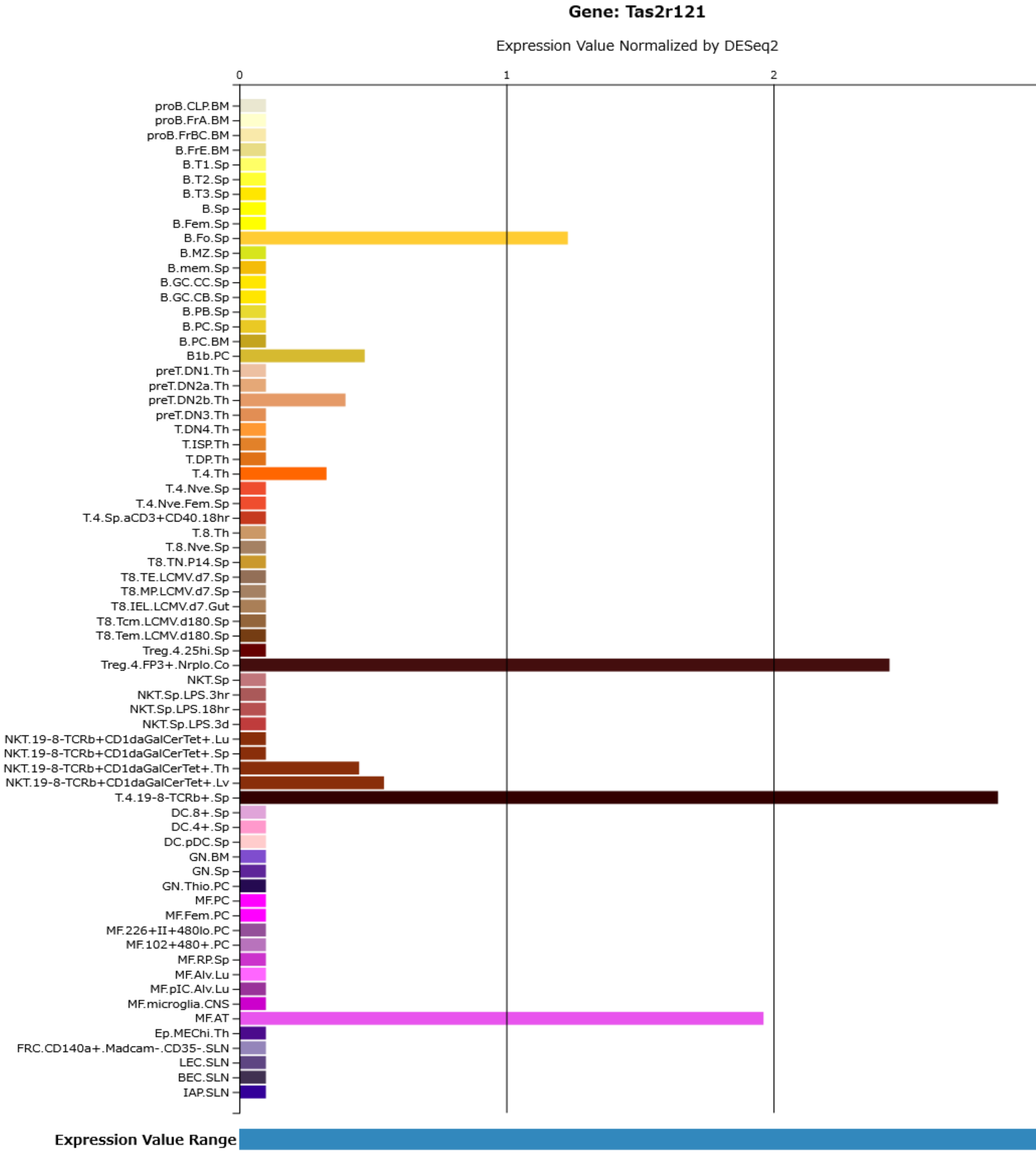
Gene: Tas2r121
Expression Value Normalized by DESeq2
0
1
2
proB.CLP.BM
proB.FrA.BM
proB.FrBC.BM
B.FrE.BM
B.T1.Sp
B.T2.Sp
B.T3.Sp
B.Sp
B.Fem.Sp
B.Fo.Sp
B.MZ.Sp
B.mem.Sp
B.GC.CC.Sp
B.GC.CB.Sp
B.PB.Sp
B.PC.Sp
B.PC.BM
B1b.PC
preT.DN1.Th
preT.DN2a.Th
preT.DN2b.Th
preT.DN3.Th
T.DN4.Th
T.ISP.Th
T.DP.Th
T.4.Th
T.4.Nve.Sp
T.4.Nve.Fem.Sp
T.4.Sp.aCD3+CD40.18hr
T.8.Th
T.8.Nve.Sp
T8.TN.P14.Sp
T8.TE.LCMV.d7.Sp
T8.MP.LCMV.d7.Sp
T8.IEL.LCMV.d7.Gut
T8.Tcm.LCMV.d180.Sp
T8.Tem.LCMV.d180.Sp
Treg.4.25hi.Sp
Treg.4.FP3+.Nrplo.Co
NKT.Sp
NKT.Sp.LPS.3hr
NKT.Sp.LPS.18hr
NKT.Sp.LPS.3d
NKT.19-8-TCRb+CD1daGalCerTet+.Lu
NKT.19-8-TCRb+CD1daGalCerTet+.Sp
NKT.19-8-TCRb+CD1daGalCerTet+.Th
NKT.19-8-TCRb+CD1daGalCerTet+.Lv
T.4.19-8-TCRb+.Sp
DC.8+.Sp
DC.4+.Sp
DC.pDC.Sp
GN.BM
GN.Sp
GN.Thio.PC
MF.PC
MF.Fem.PC
MF.226+II+480lo.PC
MF.102+480+.PC
MF.RP.Sp
MF.Alv.Lu
MF.pIC.Alv.Lu
MF.microglia.CNS
MF.AT
Ep.MEChi.Th
FRC.CD140a+.Madcam-.CD35-.SLN
LEC.SLN
BEC.SLN
IAP.SLN
Expression Value Range

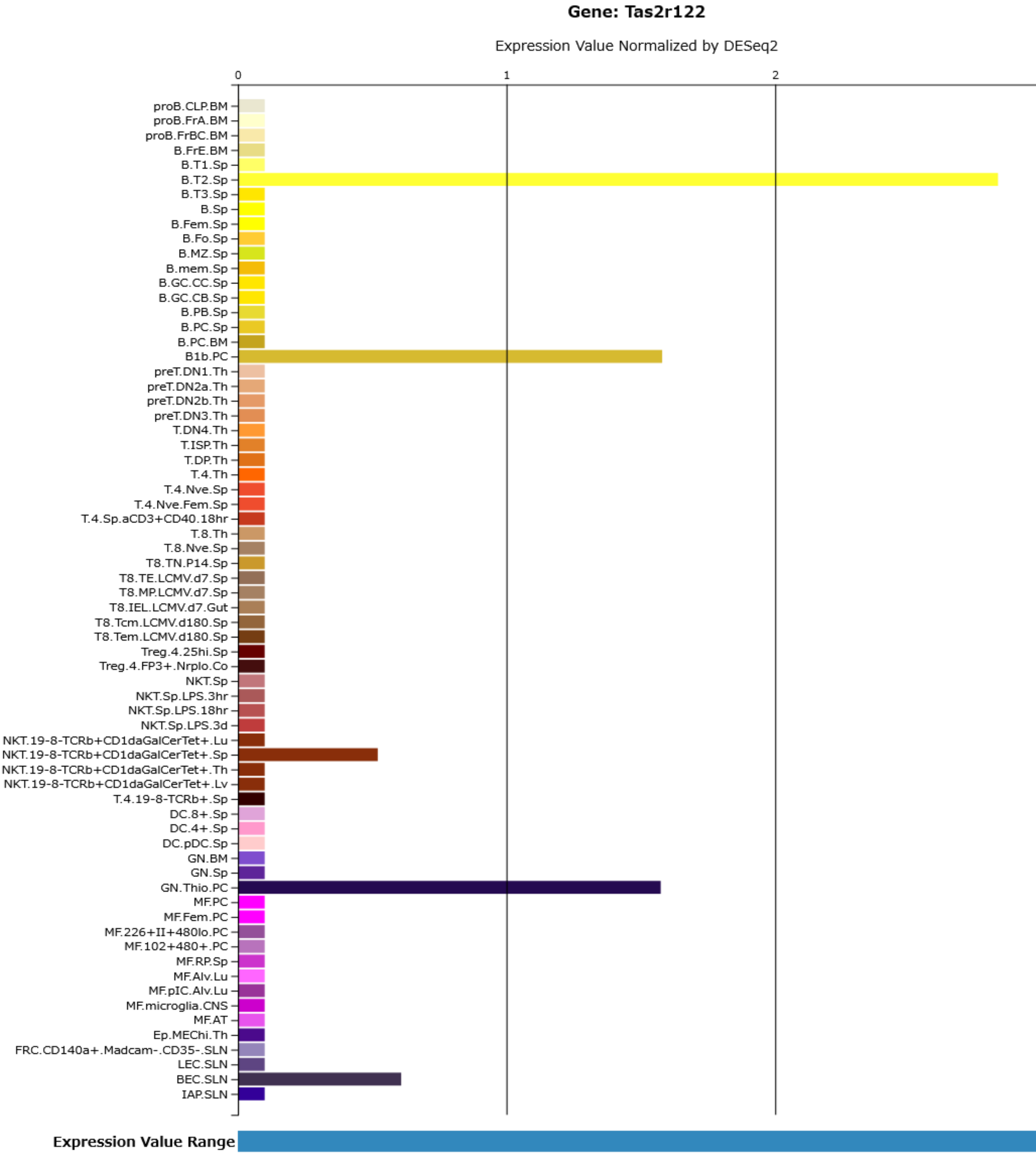

Gene: Tas2r122
Expression Value Normalized by DESeq2
0
1
2
proB.CLP.BM
proB.FrA.BM
proB.FrBC.BM
B.FrE.BM
B.T1.Sp
B.T2.Sp
B.T3.Sp
B.Sp
B.Fem.Sp
B.Fo.Sp
B.MZ.Sp
B.mem.Sp
B.GC.CC.Sp
B.GC.CB.Sp
B.PB.Sp
B.PC.Sp
B.PC.BM
B1b.PC
preT.DN1.Th
preT.DN2a.Th
preT.DN2b.Th
preT.DN3.Th
T.DN4.Th
T.ISP.Th
T.DP.Th
T.4.Th
T.4.Nve.Sp
T.4.Nve.Fem.Sp
T.4.Sp.aCD3+CD40.18hr
T.8.Th
T.8.Nve.Sp
T8.TN.P14.Sp
T8.TE.LCMV.d7.Sp
T8.MP.LCMV.d7.Sp
T8.IEL.LCMV.d7.Gut
T8.Tcm.LCMV.d180.Sp
T8.Tem.LCMV.d180.Sp
Treg.4.25hi.Sp
Treg.4.FP3+.Nrplo.Co
NKT.Sp
NKT.Sp.LPS.3hr
NKT.Sp.LPS.18hr
NKT.Sp.LPS.3d
NKT.19-8-TCRb+CD1daGalCerTet+.Lu
NKT.19-8-TCRb+CD1daGalCerTet+.Sp
NKT.19-8-TCRb+CD1daGalCerTet+.Th
NKT.19-8-TCRb+CD1daGalCerTet+.Lv
T.4.19-8-TCRb+.Sp
DC.8+.Sp
DC.4+.Sp
DC.pDC.Sp
GN.BM
GN.Sp
GN.Thio.PC
MF.PC
MF.Fem.PC
MF.226+II+480lo.PC
MF.102+480+.PC
MF.RP.Sp
MF.Alv.Lu
MF.pIC.Alv.Lu
MF.microglia.CNS
MF.AT
Ep.MEChi.Th
FRC.CD140a+.Madcam-.CD35-.SLN
LEC.SLN
BEC.SLN
IAP.SLN
Expression Value Range

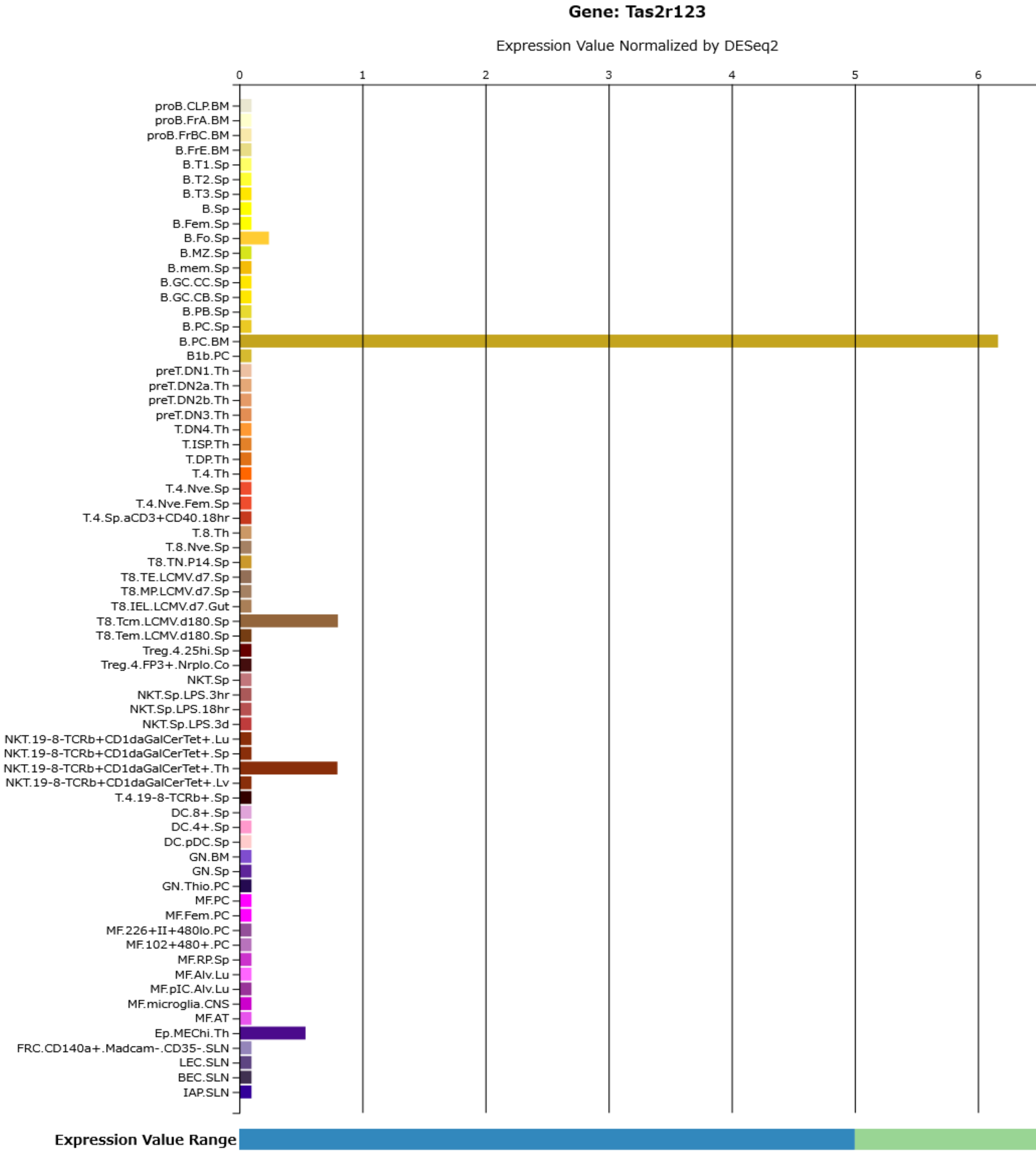
Gene: Tas2r123
Expression Value Normalized by DESeq2
0
1
2
3
4
5
6
proB.CLP.BM
proB.FrA.BM
proB.FrBC.BM
B.FrE.BM
B.T1.Sp
B.T2.Sp
B.T3.Sp
B.Sp
B.Fem.Sp
B.Fo.Sp
B.MZ.Sp
B.mem.Sp
B.GC.CC.Sp
B.GC.CB.Sp
B.PB.Sp
B.PC.Sp
B.PC.BM
B1b.PC
preT.DN1.Th
preT.DN2a.Th
preT.DN2b.Th
preT.DN3.Th
T.DN4.Th
T.ISP.Th
T.DP.Th
T.4.Th
T.4.Nve.Sp
T.4.Nve.Fem.Sp
T.4.Sp.aCD3+CD40.18hr
T.8.Th
T.8.Nve.Sp
T8.TN.P14.Sp
T8.TE.LCMV.d7.Sp
T8.MP.LCMV.d7.Sp
T8.IEL.LCMV.d7.Gut
T8.Tcm.LCMV.d180.Sp
T8.Tem.LCMV.d180.Sp
Treg.4.25hi.Sp
Treg.4.FP3+.Nrplo.Co
NKT.Sp
NKT.Sp.LPS.3hr
NKT.Sp.LPS.18hr
NKT.Sp.LPS.3d
NKT.19-8-TCRb+CD1daGalCerTet+.Lu
NKT.19-8-TCRb+CD1daGalCerTet+.Sp
NKT.19-8-TCRb+CD1daGalCerTet+.Th
NKT.19-8-TCRb+CD1daGalCerTet+.Lv
T.4.19-8-TCRb+.Sp
DC.8+.Sp
DC.4+.Sp
DC.pDC.Sp
GN.BM
GN.Sp
GN.Thio.PC
MF.PC
MF.Fem.PC
MF.226+II+480lo.PC
MF.102+480+.PC
MF.RP.Sp
MF.Alv.Lu
MF.pIC.Alv.Lu
MF.microglia.CNS
MF.AT
Ep.MEChi.Th
FRC.CD140a+.Madcam-.CD35-.SLN
LEC.SLN
BEC.SLN
IAP.SLN
Expression Value Range

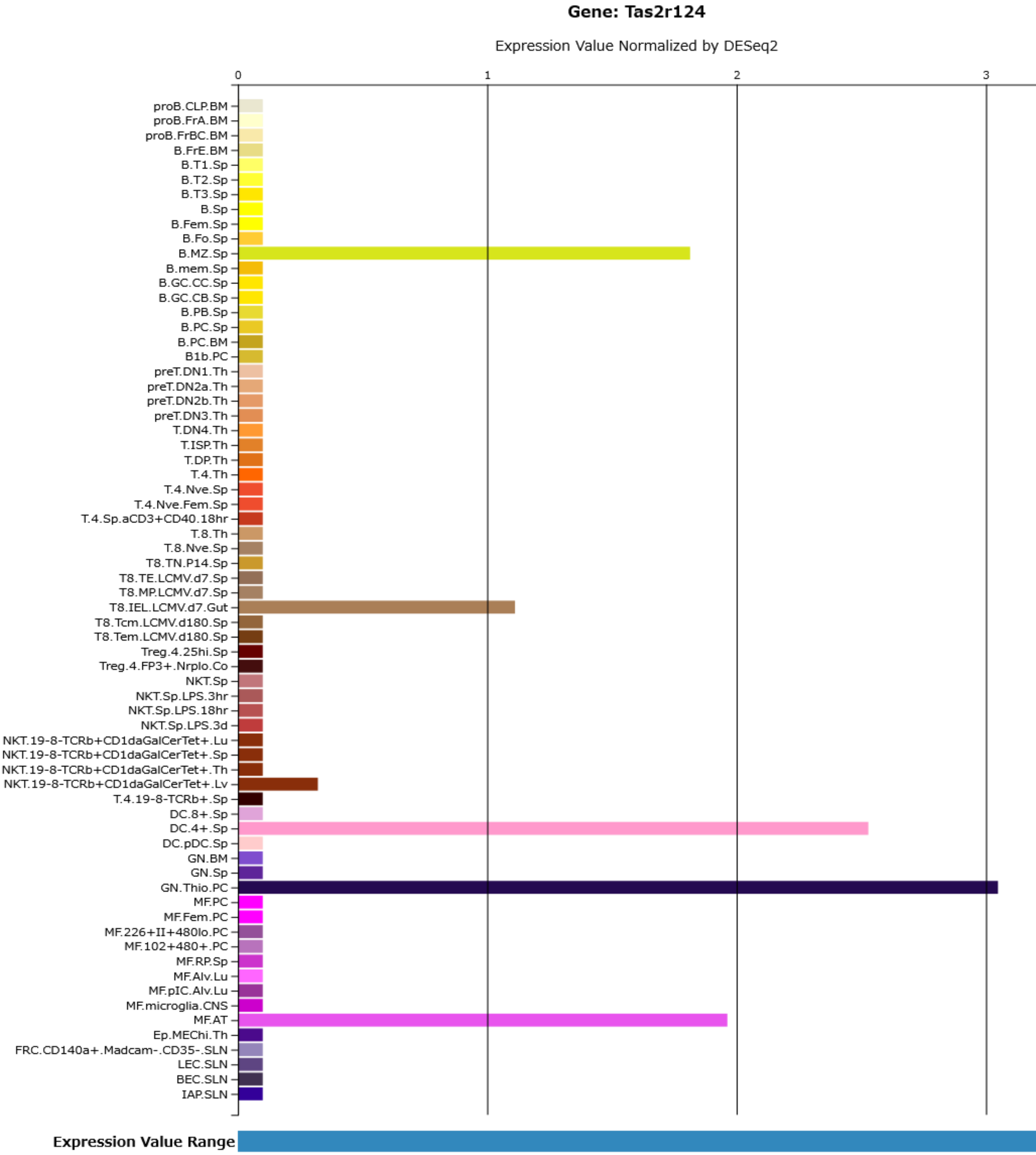
Gene: Tas2r124
Expression Value Normalized by DESeq2
0
1
2
3
proB.CLP.BM
proB.FrA.BM
proB.FrBC.BM
B.FrE.BM
B.T1.Sp
B.T2.Sp
B.T3.Sp
B.Sp
B.Fem.Sp
B.Fo.Sp
B.MZ.Sp
B.mem.Sp
B.GC.CC.Sp
B.GC.CB.Sp
B.PB.Sp
B.PC.Sp
B.PC.BM
B1b.PC
preT.DN1.Th
preT.DN2a.Th
preT.DN2b.Th
preT.DN3.Th
T.DN4.Th
T.ISP.Th
T.DP.Th
T.4.Th
T.4.Nve.Sp
T.4.Nve.Fem.Sp
T.4.Sp.aCD3+CD40.18hr
T.8.Th
T.8.Nve.Sp
T8.TN.P14.Sp
T8.TE.LCMV.d7.Sp
T8.MP.LCMV.d7.Sp
T8.IEL.LCMV.d7.Gut
T8.Tcm.LCMV.d180.Sp
T8.Tem.LCMV.d180.Sp
Treg.4.25hi.Sp
Treg.4.FP3+.Nrplo.Co
NKT.Sp
NKT.Sp.LPS.3hr
NKT.Sp.LPS.18hr
NKT.Sp.LPS.3d
NKT.19-8-TCRb+CD1daGalCerTet+.Lu
NKT.19-8-TCRb+CD1daGalCerTet+.Sp
NKT.19-8-TCRb+CD1daGalCerTet+.Th
NKT.19-8-TCRb+CD1daGalCerTet+.Lv
T.4.19-8-TCRb+.Sp
DC.8+.Sp
DC.4+.Sp
DC.pDC.Sp
GN.BM
GN.Sp
GN.Thio.PC
MF.PC
MF.Fem.PC
MF.226+II+480lo.PC
MF.102+480+.PC
MF.RP.Sp
MF.Alv.Lu
MF.pIC.Alv.Lu
MF.microglia.CNS
MF.AT
Ep.MEChi.Th
FRC.CD140a+.Madcam-.CD35-.SLN
LEC.SLN
BEC.SLN
IAP.SLN
Expression Value Range

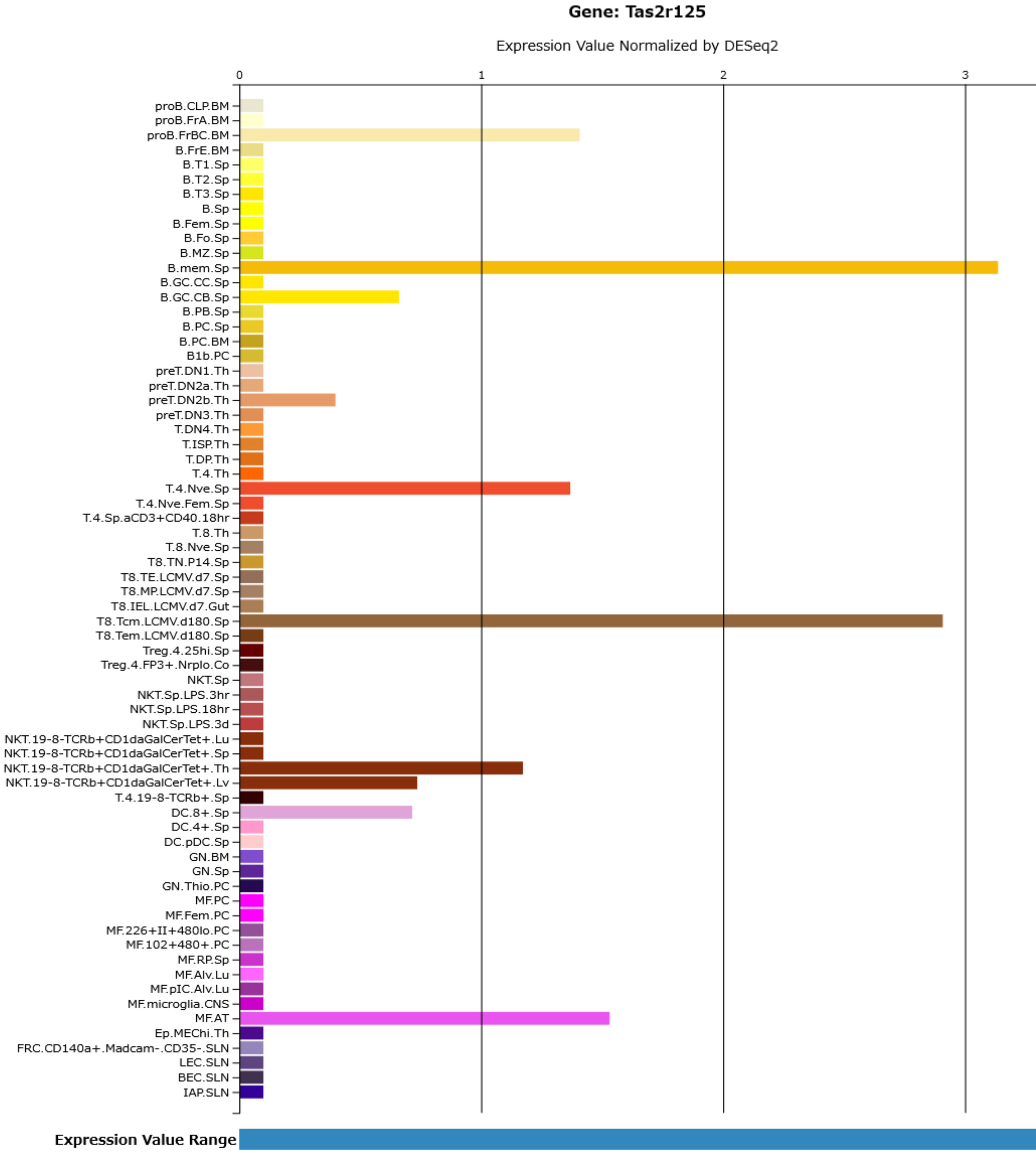

Gene: Tas2r125
Expression Value Normalized by DESeq2
0
1
2
3
proB.CLP.BM
proB.FrA.BM
proB.FrBC.BM
B.FrE.BM
B.T1.Sp
B.T2.Sp
B.T3.Sp
B.Sp
B.Fem.Sp
B.Fo.Sp
B.MZ.Sp
B.mem.Sp
B.GC.CC.Sp
B.GC.CB.Sp
B.PB.Sp
B.PC.Sp
B.PC.BM
B1b.PC
preT.DN1.Th
preT.DN2a.Th
preT.DN2b.Th
preT.DN3.Th
T.DN4.Th
T.ISP.Th
T.DP.Th
T.4.Th
T.4.Nve.Sp
T.4.Nve.Fem.Sp
T.4.Sp.aCD3+CD40.18hr
T.8.Th
T.8.Nve.Sp
T8.TN.P14.Sp
T8.TE.LCMV.d7.Sp
T8.MP.LCMV.d7.Sp
T8.IEL.LCMV.d7.Gut
T8.Tcm.LCMV.d180.Sp
T8.Tem.LCMV.d180.Sp
Treg.4.25hi.Sp
Treg.4.FP3+.Nrplo.Co
NKT.Sp
NKT.Sp.LPS.3hr
NKT.Sp.LPS.18hr
NKT.Sp.LPS.3d
NKT.19-8-TCRb+CD1daGalCerTet+.Lu
NKT.19-8-TCRb+CD1daGalCerTet+.Sp
NKT.19-8-TCRb+CD1daGalCerTet+.Th
NKT.19-8-TCRb+CD1daGalCerTet+.Lv
T.4.19-8-TCRb+.Sp
DC.8+.Sp
DC.4+.Sp
DC.pDC.Sp
GN.BM
GN.Sp
GN.Thio.PC
MF.PC
MF.Fem.PC
MF.226+II+480lo.PC
MF.102+480+.PC
MF.RP.Sp
MF.Alv.Lu
MF.pIC.Alv.Lu
MF.microglia.CNS
MF.AT
Ep.MEChi.Th
FRC.CD140a+.Madcam-.CD35-.SLN
LEC.SLN
BEC.SLN
IAP.SLN
Expression Value Range

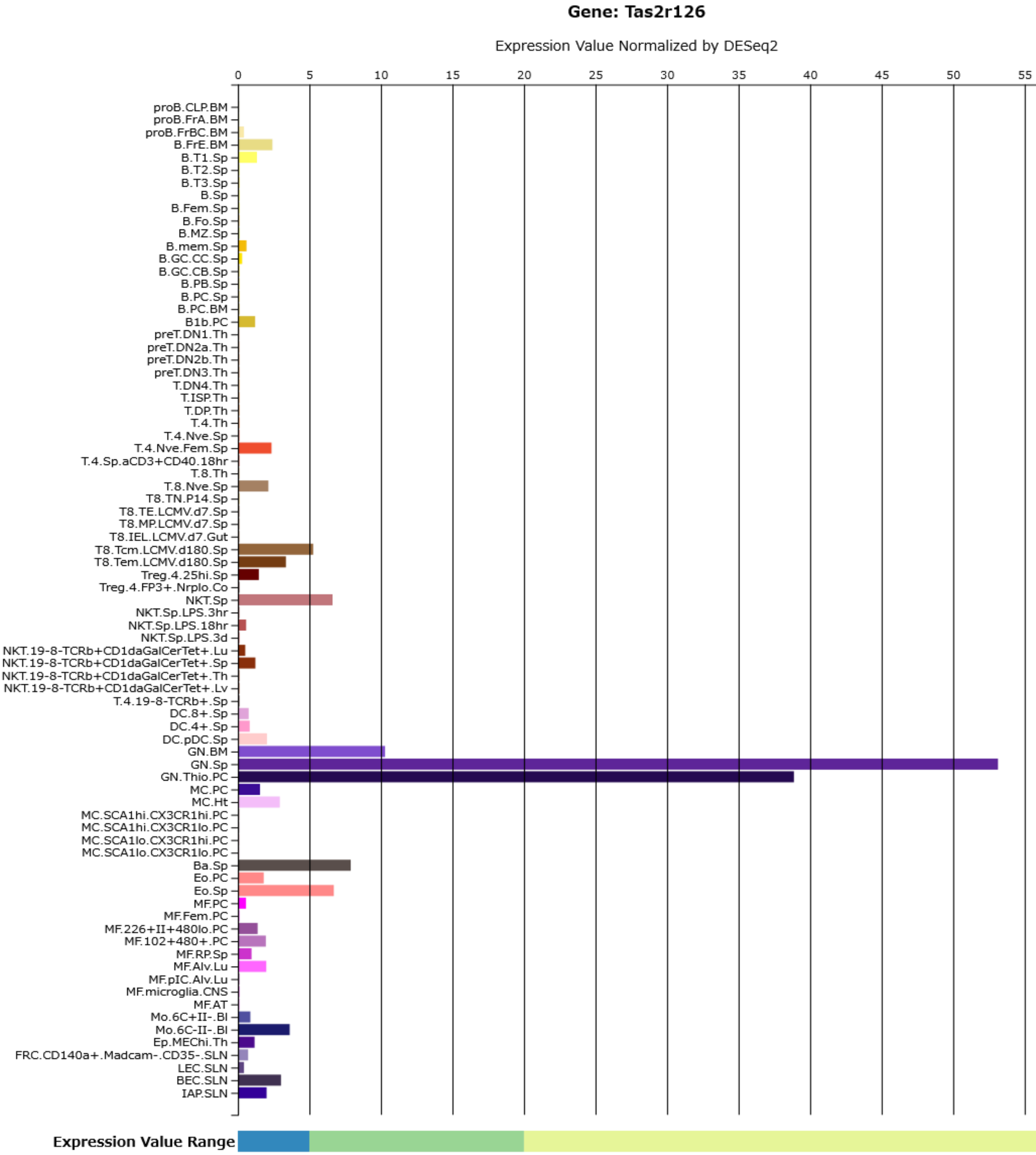

Gene: Tas2r126
Expression Value Normalized by DESeq2
0
5
10
15
20
25
30
35
40
45
50
55
proB.CLP.BM
proB.FrA.BM
proB.FrBC.BM
B.FrE.BM
B.T1.Sp
B.T2.Sp
B.T3.Sp
B.Sp
B.Fem.Sp
B.Fo.Sp
B.MZ.Sp
B.mem.Sp
B.GC.CC.Sp
B.GC.CB.Sp
B.PB.Sp
B.PC.Sp
B.PC.BM
B1b.PC
preT.DN1.Th
preT.DN2a.Th
preT.DN2b.Th
preT.DN3.Th
T.DN4.Th
T.ISP.Th
T.DP.Th
T.4.Th
T.4.Nve.Sp
T.4.Nve.Fem.Sp
T.4.Sp.aCD3+CD40.18hr
T.8.Th
T.8.Nve.Sp
T8.TN.P14.Sp
T8.TE.LCMV.d7.Sp
T8.MP.LCMV.d7.Sp
T8.IEL.LCMV.d7.Gut
T8.Tcm.LCMV.d180.Sp
T8.Tem.LCMV.d180.Sp
Treg.4.25hi.Sp
Treg.4.FP3+.Nrplo.Co
NKT.Sp
NKT.Sp.LPS.3hr
NKT.Sp.LPS.18hr
NKT.Sp.LPS.3d
NKT.19-8-TCRb+CD1daGalCerTet+.Lu
NKT.19-8-TCRb+CD1daGalCerTet+.Sp
NKT.19-8-TCRb+CD1daGalCerTet+.Th
NKT.19-8-TCRb+CD1daGalCerTet+.Lv
T.4.19-8-TCRb+.Sp
DC.8+.Sp
DC.4+.Sp
DC.pDC.Sp
GN.BM
GN.Sp
GN.Thio.PC
MC.PC
MC.Ht
MC.SCA1hi.CX3CR1hi.PC
MC.SCA1hi.CX3CR1lo.PC
MC.SCA1lo.CX3CR1hi.PC
MC.SCA1lo.CX3CR1lo.PC
Ba.Sp
Eo.PC
Eo.Sp
MF.PC
MF.Fem.PC
MF.226+II+480lo.PC
MF.102+480+.PC
MF.RP.Sp
MF.Alv.Lu
MF.pIC.Alv.Lu
MF.microglia.CNS
MF.AT
Mo.6C+II-.Bl
Mo.6C-II-.Bl
Ep.MEChi.Th
FRC.CD140a+.Madcam-.CD35-.SLN
LEC.SLN
BEC.SLN
IAP.SLN
Expression Value Range

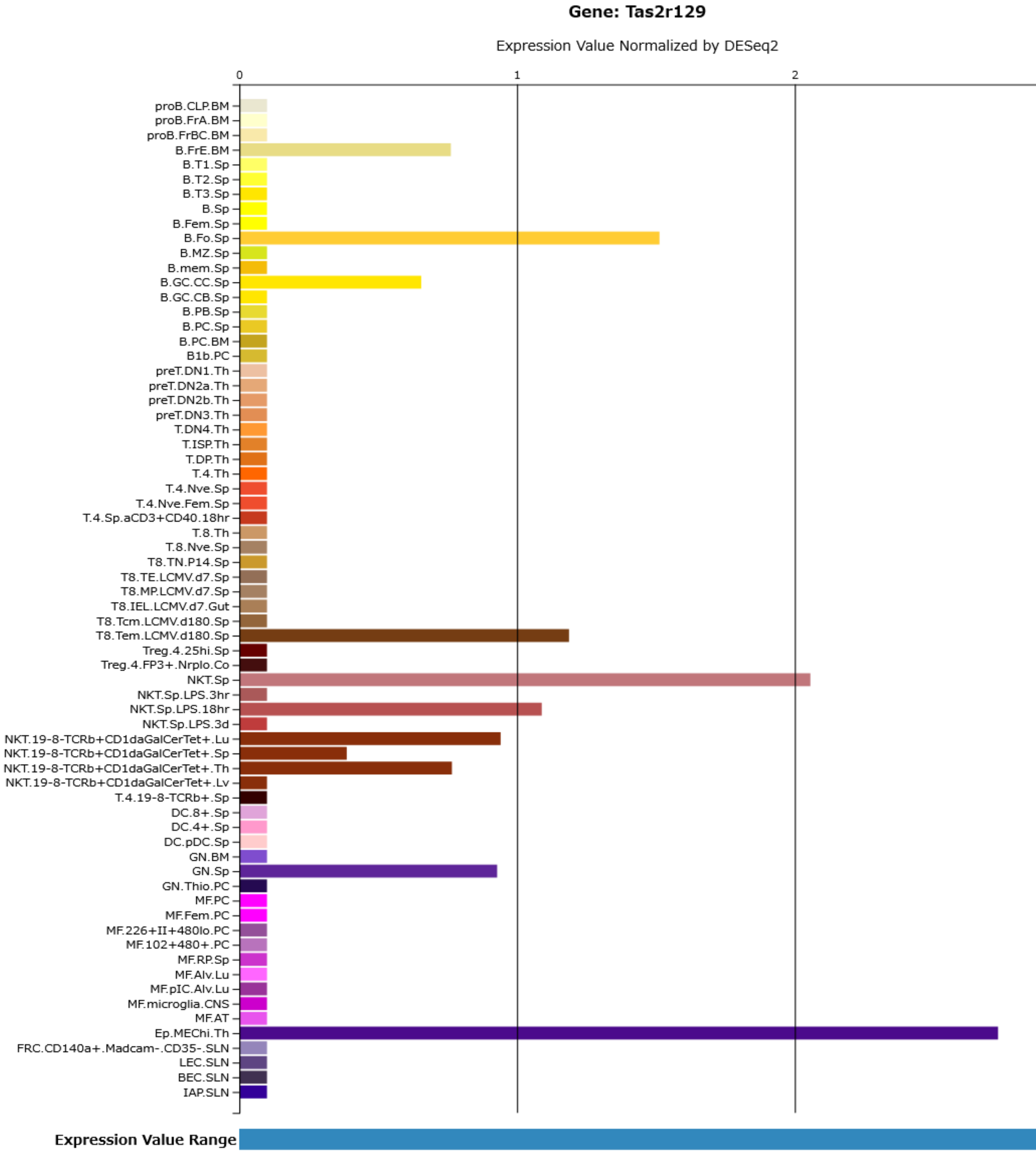

Gene: Tas2r129
Expression Value Normalized by DESeq2
0
1
2
proB.CLP.BM
proB.FrA.BM
proB.FrBC.BM
B.FrE.BM
B.T1.Sp
B.T2.Sp
B.T3.Sp
B.Sp
B.Fem.Sp
B.Fo.Sp
B.MZ.Sp
B.mem.Sp
B.GC.CC.Sp
B.GC.CB.Sp
B.PB.Sp
B.PC.Sp
B.PC.BM
B1b.PC
preT.DN1.Th
preT.DN2a.Th
preT.DN2b.Th
preT.DN3.Th
T.DN4.Th
T.ISP.Th
T.DP.Th
T.4.Th
T.4.Nve.Sp
T.4.Nve.Fem.Sp
T.4.Sp.aCD3+CD40.18hr
T.8.Th
T.8.Nve.Sp
T8.TN.P14.Sp
T8.TE.LCMV.d7.Sp
T8.MP.LCMV.d7.Sp
T8.IEL.LCMV.d7.Gut
T8.Tcm.LCMV.d180.Sp
T8.Tem.LCMV.d180.Sp
Treg.4.25hi.Sp
Treg.4.FP3+.Nrplo.Co
NKT.Sp
NKT.Sp.LPS.3hr
NKT.Sp.LPS.18hr
NKT.Sp.LPS.3d
NKT.19-8-TCRb+CD1daGalCerTet+.Lu
NKT.19-8-TCRb+CD1daGalCerTet+.Sp
NKT.19-8-TCRb+CD1daGalCerTet+.Th
NKT.19-8-TCRb+CD1daGalCerTet+.Lv
T.4.19-8-TCRb+.Sp
DC.8+.Sp
DC.4+.Sp
DC.pDC.Sp
GN.BM
GN.Sp
GN.Thio.PC
MF.PC
MF.Fem.PC
MF.226+II+480lo.PC
MF.102+480+.PC
MF.RP.Sp
MF.Alv.Lu
MF.pIC.Alv.Lu
MF.microglia.CNS
MF.AT
Ep.MEChi.Th
FRC.CD140a+.Madcam-.CD35-.SLN
LEC.SLN
BEC.SLN
IAP.SLN
Expression Value Range

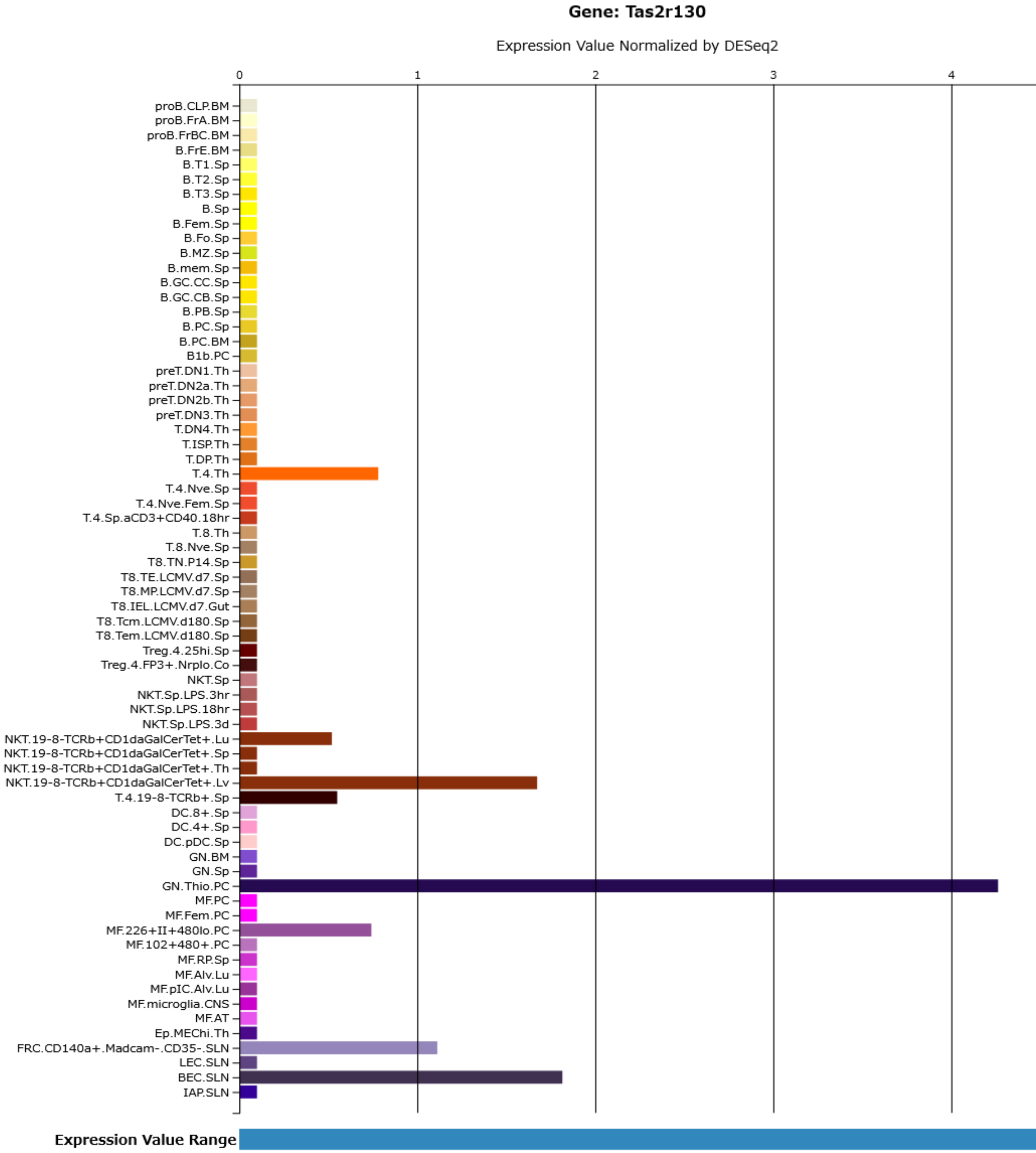

Gene: Tas2r130
Expression Value Normalized by DESeq2
0
1
2
3
4
proB.CLP.BM
proB.FrA.BM
proB.FrBC.BM
B.FrE.BM
B.T1.Sp
B.T2.Sp
B.T3.Sp
B.Sp
B.Fem.Sp
B.Fo.Sp
B.MZ.Sp
B.mem.Sp
B.GC.CC.Sp
B.GC.CB.Sp
B.PB.Sp
B.PC.Sp
B.PC.BM
B1b.PC
preT.DN1.Th
preT.DN2a.Th
preT.DN2b.Th
preT.DN3.Th
T.DN4.Th
T.ISP.Th
T.DP.Th
T.4.Th
T.4.Nve.Sp
T.4.Nve.Fem.Sp
T.4.Sp.aCD3+CD40.18hr
T.8.Th
T.8.Nve.Sp
T8.TN.P14.Sp
T8.TE.LCMV.d7.Sp
T8.MP.LCMV.d7.Sp
T8.IEL.LCMV.d7.Gut
T8.Tcm.LCMV.d180.Sp
T8.Tem.LCMV.d180.Sp
Treg.4.25hi.Sp
Treg.4.FP3+.Nrplo.Co
NKT.Sp
NKT.Sp.LPS.3hr
NKT.Sp.LPS.18hr
NKT.Sp.LPS.3d
NKT.19-8-TCRb+CD1daGalCerTet+.Lu
NKT.19-8-TCRb+CD1daGalCerTet+.Sp
NKT.19-8-TCRb+CD1daGalCerTet+.Th
NKT.19-8-TCRb+CD1daGalCerTet+.Lv
T.4.19-8-TCRb+.Sp
DC.8+.Sp
DC.4+.Sp
DC.pDC.Sp
GN.BM
GN.Sp
GN.Thio.PC
MF.PC
MF.Fem.PC
MF.226+II+480lo.PC
MF.102+480+.PC
MF.RP.Sp
MF.Alv.Lu
MF.pIC.Alv.Lu
MF.microglia.CNS
MF.AT
Ep.MEChi.Th
FRC.CD140a+.Madcam-.CD35-.SLN
LEC.SLN
BEC.SLN
IAP.SLN
Expression Value Range

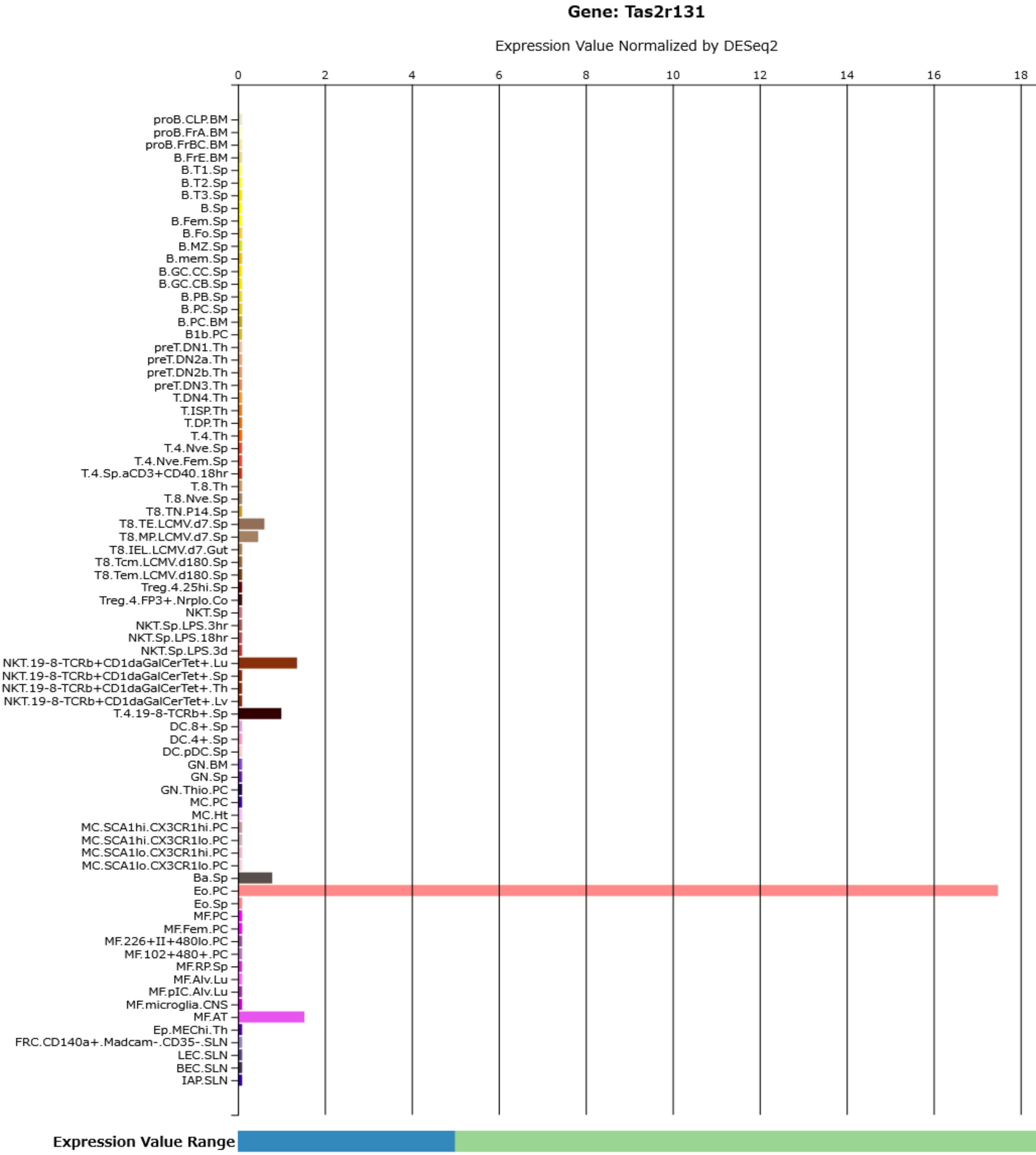

Gene: Tas2r131
Expression Value Normalized by DESeq2
0
2
4
6
8
10
12
14
16
18
proB.CLP.BM
proB.FrA.BM
proB.FrBC.BM
B.FrE.BM
B.T1.Sp
B.T2.Sp
B.T3.Sp
B.Sp
B.Fem.Sp
B.Fo.Sp
B.MZ.Sp
B.mem.Sp
B.GC.CC.Sp
B.GC.CB.Sp
B.PB.Sp
B.PC.Sp
B.PC.BM
B1b.PC
preT.DN1.Th
preT.DN2a.Th
preT.DN2b.Th
preT.DN3.Th
T.DN4.Th
T.ISP.Th
T.DP.Th
T.4.Th
T.4.Nve.Sp
T.4.Nve.Fem.Sp
T.4.Sp.aCD3+CD40.18hr
T.8.Th
T.8.Nve.Sp
T8.TN.P14.Sp
T8.TE.LCMV.d7.Sp
T8.MP.LCMV.d7.Sp
T8.IEL.LCMV.d7.Gut
T8.Tcm.LCMV.d180.Sp
T8.Tem.LCMV.d180.Sp
Treg.4.25hi.Sp
Treg.4.FP3+.Nrplo.Co
NKT.Sp
NKT.Sp.LPS.3hr
NKT.Sp.LPS.18hr
NKT.Sp.LPS.3d
NKT.19-8-TCRb+CD1daGalCerTet+.Lu
NKT.19-8-TCRb+CD1daGalCerTet+.Sp
NKT.19-8-TCRb+CD1daGalCerTet+.Th
NKT.19-8-TCRb+CD1daGalCerTet+.Lv
T.4.19-8-TCRb+.Sp
DC.8+.Sp
DC.4+.Sp
DC.pDC.Sp
GN.BM
GN.Sp
GN.Thio.PC
MC.PC
MC.Ht
MC.SCA1hi.CX3CR1hi.PC
MC.SCA1hi.CX3CR1lo.PC
MC.SCA1lo.CX3CR1hi.PC
MC.SCA1lo.CX3CR1lo.PC
Ba.Sp
Eo.PC
Eo.Sp
MF.PC
MF.Fem.PC
MF.226+II+480lo.PC
MF.102+480+.PC
MF.RP.Sp
MF.Alv.Lu
MF.pIC.Alv.Lu
MF.microglia.CNS
MF.AT
Ep.MEChi.Th
FRC.CD140a+.Madcam-.CD35-.SLN
LEC.SLN
BEC.SLN
IAP.SLN
Expression Value Range

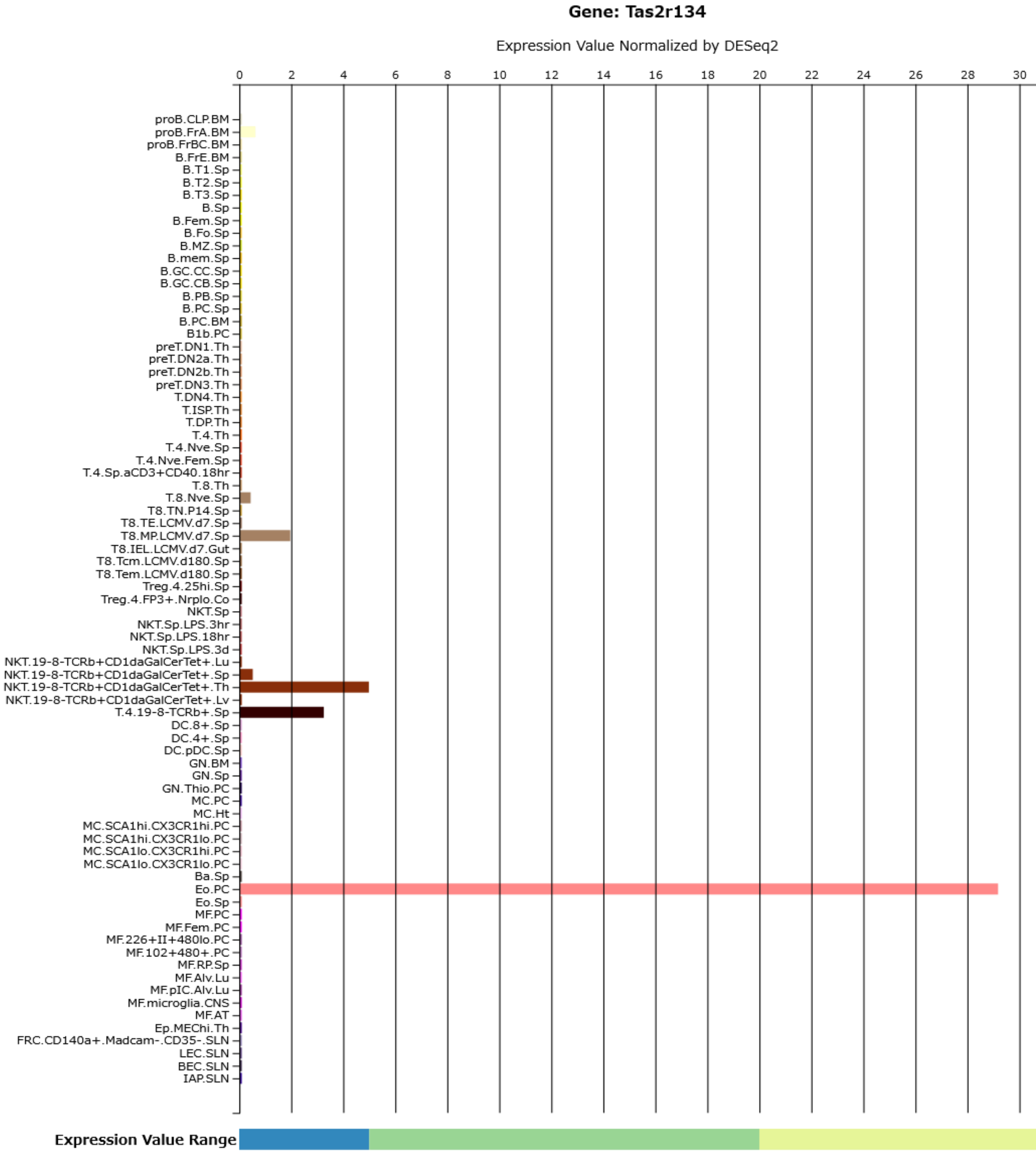

Gene: Tas2r134
Expression Value Normalized by DESeq2
0
2
4
6
8
10
12
14
16
18
20
22
24
26
28
30
proB.CLP.BM
proB.FrA.BM
proB.FrBC.BM
B.FrE.BM
B.T1.Sp
B.T2.Sp
B.T3.Sp
B.Sp
B.Fem.Sp
B.Fo.Sp
B.MZ.Sp
B.mem.Sp
B.GC.CC.Sp
B.GC.CB.Sp
B.PB.Sp
B.PC.Sp
B.PC.BM
B1b.PC
preT.DN1.Th
preT.DN2a.Th
preT.DN2b.Th
preT.DN3.Th
T.DN4.Th
T.ISP.Th
T.DP.Th
T.4.Th
T.4.Nve.Sp
T.4.Nve.Fem.Sp
T.4.Sp.aCD3+CD40.18hr
T.8.Th
T.8.Nve.Sp
T8.TN.P14.Sp
T8.TE.LCMV.d7.Sp
T8.MP.LCMV.d7.Sp
T8.IEL.LCMV.d7.Gut
T8.Tcm.LCMV.d180.Sp
T8.Tem.LCMV.d180.Sp
Treg.4.25hi.Sp
Treg.4.FP3+.Nrplo.Co
NKT.Sp
NKT.Sp.LPS.3hr
NKT.Sp.LPS.18hr
NKT.Sp.LPS.3d
NKT.19-8-TCRb+CD1daGalCerTet+.Lu
NKT.19-8-TCRb+CD1daGalCerTet+.Sp
NKT.19-8-TCRb+CD1daGalCerTet+.Th
NKT.19-8-TCRb+CD1daGalCerTet+.Lv
T.4.19-8-TCRb+.Sp
DC.8+.Sp
DC.4+.Sp
DC.pDC.Sp
GN.BM
GN.Sp
GN.Thio.PC
MC.PC
MC.Ht
MC.SCA1hi.CX3CR1hi.PC
MC.SCA1hi.CX3CR1lo.PC
MC.SCA1lo.CX3CR1hi.PC
MC.SCA1lo.CX3CR1lo.PC
Ba.Sp
Eo.PC
Eo.Sp
MF.PC
MF.Fem.PC
MF.226+II+480lo.PC
MF.102+480+.PC
MF.RP.Sp
MF.Alv.Lu
MF.pIC.Alv.Lu
MF.microglia.CNS
MF.AT
Ep.MEChi.Th
FRC.CD140a+.Madcam-.CD35-.SLN
LEC.SLN
BEC.SLN
IAP.SLN
Expression Value Range

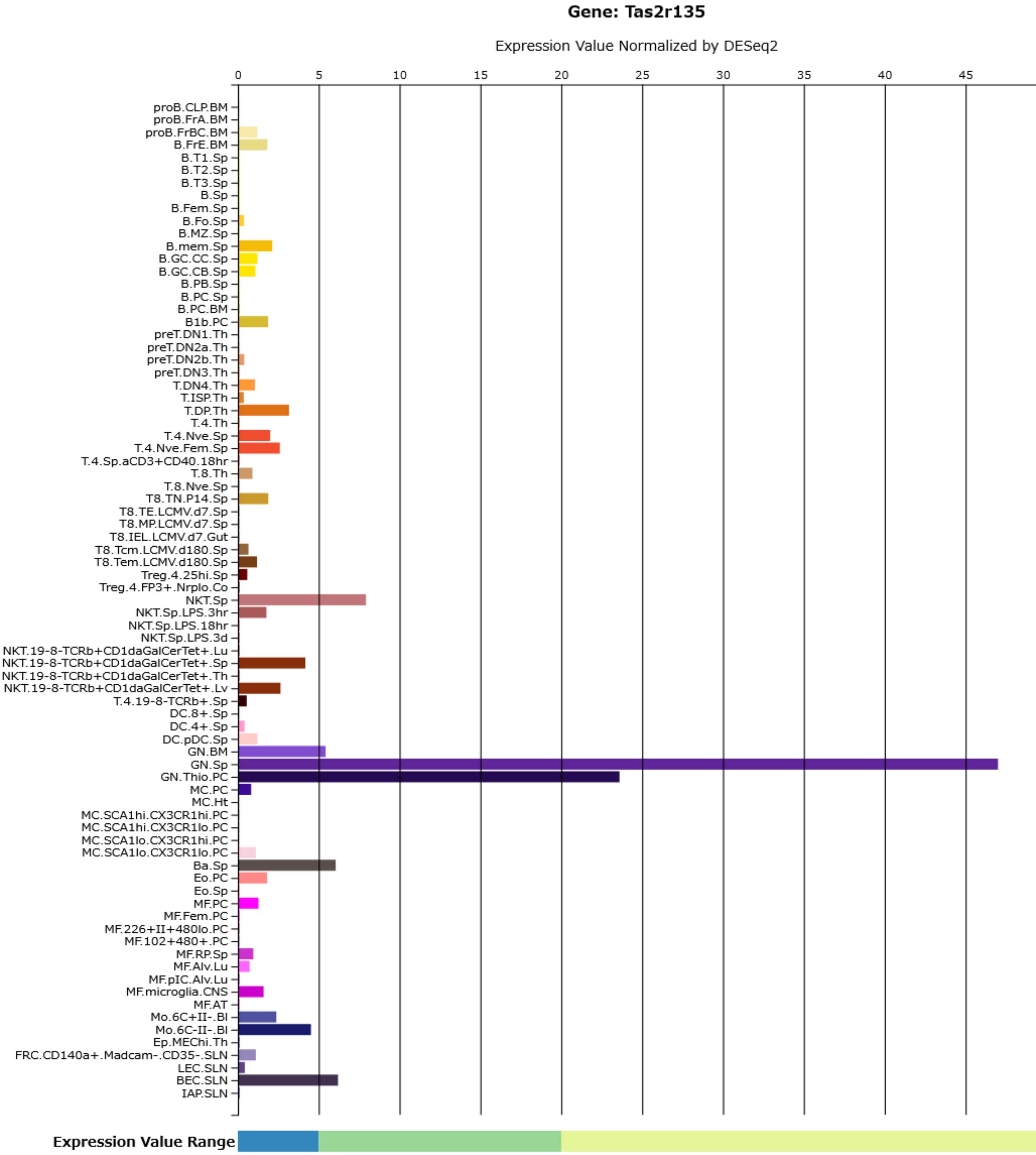
Gene: Tas2r135
Expression Value Normalized by DESeq2
0
5
10
15
20
25
30
35
40
45
proB.CLP.BM
proB.FrA.BM
proB.FrBC.BM
B.FrE.BM
B.T1.Sp
B.T2.Sp
B.T3.Sp
B.Sp
B.Fem.Sp
B.Fo.Sp
B.MZ.Sp
B.mem.Sp
B.GC.CC.Sp
B.GC.CB.Sp
B.PB.Sp
B.PC.Sp
B.PC.BM
B1b.PC
preT.DN1.Th
preT.DN2a.Th
preT.DN2b.Th
preT.DN3.Th
T.DN4.Th
T.ISP.Th
T.DP.Th
T.4.Th
T.4.Nve.Sp
T.4.Nve.Fem.Sp
T.4.Sp.aCD3+CD40.18hr
T.8.Th
T.8.Nve.Sp
T8.TN.P14.Sp
T8.TE.LCMV.d7.Sp
T8.MP.LCMV.d7.Sp
T8.IEL.LCMV.d7.Gut
T8.Tcm.LCMV.d180.Sp
T8.Tem.LCMV.d180.Sp
Treg.4.25hi.Sp
Treg.4.FP3+.Nrplo.Co
NKT.Sp
NKT.Sp.LPS.3hr
NKT.Sp.LPS.18hr
NKT.Sp.LPS.3d
NKT.19-8-TCRb+CD1daGalCerTet+.Lu
NKT.19-8-TCRb+CD1daGalCerTet+.Sp
NKT.19-8-TCRb+CD1daGalCerTet+.Th
NKT.19-8-TCRb+CD1daGalCerTet+.Lv
T.4.19-8-TCRb+.Sp
DC.8+.Sp
DC.4+.Sp
DC.pDC.Sp
GN.BM
GN.Sp
GN.Thio.PC
MC.PC
MC.Ht
MC.SCA1hi.CX3CR1hi.PC
MC.SCA1hi.CX3CR1lo.PC
MC.SCA1lo.CX3CR1hi.PC
MC.SCA1lo.CX3CR1lo.PC
Ba.Sp
Eo.PC
Eo.Sp
MF.PC
MF.Fem.PC
MF.226+II+480lo.PC
MF.102+480+.PC
MF.RP.Sp
MF.Alv.Lu
MF.pIC.Alv.Lu
MF.microglia.CNS
MF.AT
Mo.6C+II-.Bl
Mo.6C-II-.Bl
Ep.MEChi.Th
FRC.CD140a+.Madcam-.CD35-.SLN
LEC.SLN
BEC.SLN
IAP.SLN
Expression Value Range

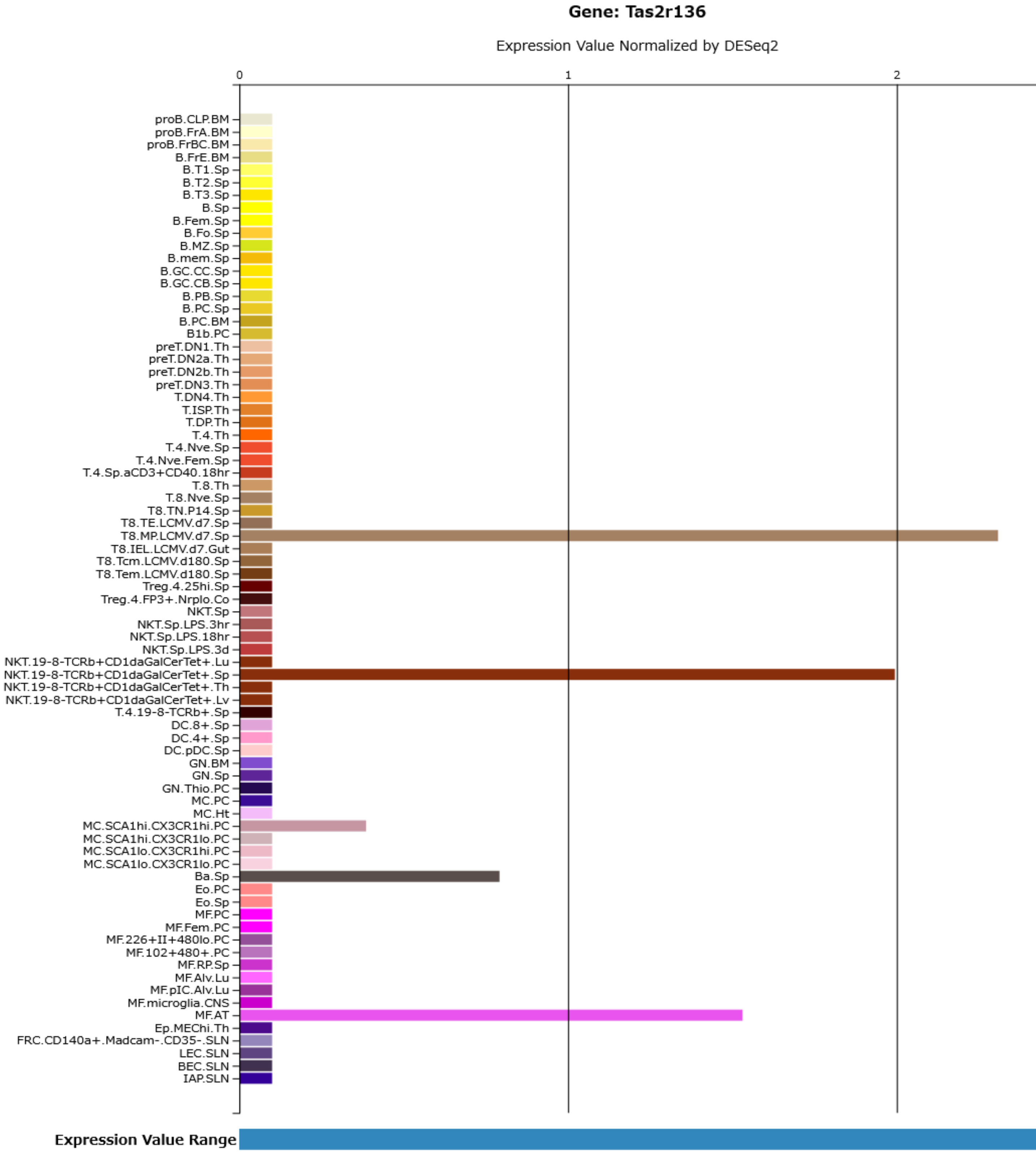
Gene: Tas2r136
Expression Value Normalized by DESeq2
0
1
2
proB.CLP.BM
proB.FrA.BM
proB.FrBC.BM
B.FrE.BM
B.T1.Sp
B.T2.Sp
B.T3.Sp
B.Sp
B.Fem.Sp
B.Fo.Sp
B.MZ.Sp
B.mem.Sp
B.GC.CC.Sp
B.GC.CB.Sp
B.PB.Sp
B.PC.Sp
B.PC.BM
B1b.PC
preT.DN1.Th
preT.DN2a.Th
preT.DN2b.Th
preT.DN3.Th
T.DN4.Th
T.ISP.Th
T.DP.Th
T.4.Th
T.4.Nve.Sp
T.4.Nve.Fem.Sp
T.4.Sp.aCD3+CD40.18hr
T.8.Th
T.8.Nve.Sp
T8.TN.P14.Sp
T8.TE.LCMV.d7.Sp
T8.MP.LCMV.d7.Sp
T8.IEL.LCMV.d7.Gut
T8.Tcm.LCMV.d180.Sp
T8.Tem.LCMV.d180.Sp
Treg.4.25hi.Sp
Treg.4.FP3+.Nrplo.Co
NKT.Sp
NKT.Sp.LPS.3hr
NKT.Sp.LPS.18hr
NKT.Sp.LPS.3d
NKT.19-8-TCRb+CD1daGalCerTet+.Lu
NKT.19-8-TCRb+CD1daGalCerTet+.Sp
NKT.19-8-TCRb+CD1daGalCerTet+.Th
NKT.19-8-TCRb+CD1daGalCerTet+.Lv
T.4.19-8-TCRb+.Sp
DC.8+.Sp
DC.4+.Sp
DC.pDC.Sp
GN.BM
GN.Sp
GN.Thio.PC
MC.PC
MC.Ht
MC.SCA1hi.CX3CR1hi.PC
MC.SCA1hi.CX3CR1lo.PC
MC.SCA1lo.CX3CR1hi.PC
MC.SCA1lo.CX3CR1lo.PC
Ba.Sp
Eo.PC
Eo.Sp
MF.PC
MF.Fem.PC
MF.226+II+480lo.PC
MF.102+480+.PC
MF.RP.Sp
MF.Alv.Lu
MF.pIC.Alv.Lu
MF.microglia.CNS
MF.AT
Ep.MEChi.Th
FRC.CD140a+.Madcam-.CD35-.SLN
LEC.SLN
BEC.SLN
IAP.SLN
Expression Value Range

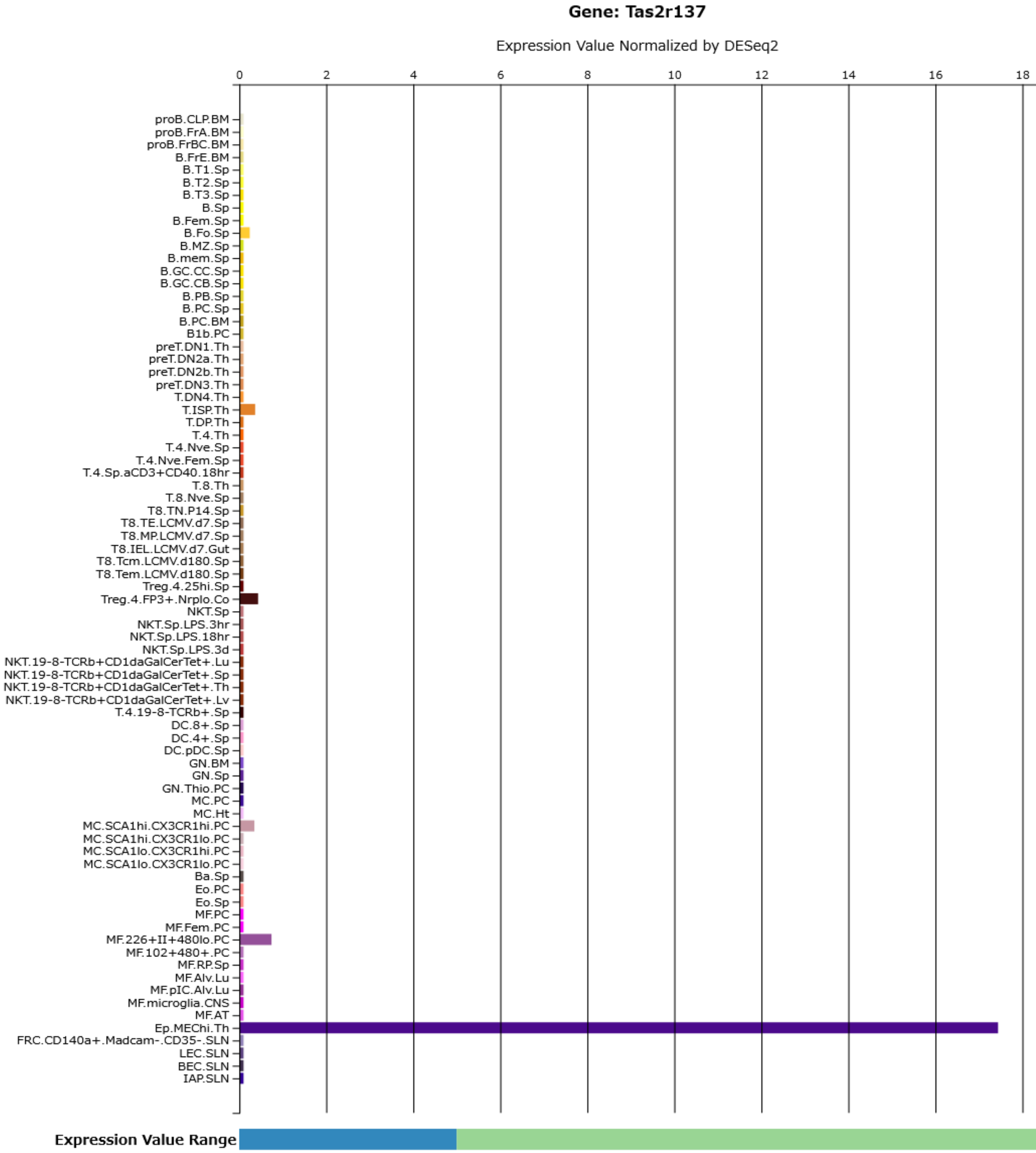
Gene: Tas2r137
Expression Value Normalized by DESeq2
0
2
4
6
8
10
12
14
16
18
proB.CLP.BM
proB.FrA.BM
proB.FrBC.BM
B.FrE.BM
B.T1.Sp
B.T2.Sp
B.T3.Sp
B.Sp
B.Fem.Sp
B.Fo.Sp
B.MZ.Sp
B.mem.Sp
B.GC.CC.Sp
B.GC.CB.Sp
B.PB.Sp
B.PC.Sp
B.PC.BM
B1b.PC
preT.DN1.Th
preT.DN2a.Th
preT.DN2b.Th
preT.DN3.Th
T.DN4.Th
T.ISP.Th
T.DP.Th
T.4.Th
T.4.Nve.Sp
T.4.Nve.Fem.Sp
T.4.Sp.aCD3+CD40.18hr
T.8.Th
T.8.Nve.Sp
T8.TN.P14.Sp
T8.TE.LCMV.d7.Sp
T8.MP.LCMV.d7.Sp
T8.IEL.LCMV.d7.Gut
T8.Tcm.LCMV.d180.Sp
T8.Tem.LCMV.d180.Sp
Treg.4.25hi.Sp
Treg.4.FP3+.Nrplo.Co
NKT.Sp
NKT.Sp.LPS.3hr
NKT.Sp.LPS.18hr
NKT.Sp.LPS.3d
NKT.19-8-TCRb+CD1daGalCerTet+.Lu
NKT.19-8-TCRb+CD1daGalCerTet+.Sp
NKT.19-8-TCRb+CD1daGalCerTet+.Th
NKT.19-8-TCRb+CD1daGalCerTet+.Lv
T.4.19-8-TCRb+.Sp
DC.8+.Sp
DC.4+.Sp
DC.pDC.Sp
GN.BM
GN.Sp
GN.Thio.PC
MC.PC
MC.Ht
MC.SCA1hi.CX3CR1hi.PC
MC.SCA1hi.CX3CR1lo.PC
MC.SCA1lo.CX3CR1hi.PC
MC.SCA1lo.CX3CR1lo.PC
Ba.Sp
Eo.PC
Eo.Sp
MF.PC
MF.Fem.PC
MF.226+II+480lo.PC
MF.102+480+.PC
MF.RP.Sp
MF.Alv.Lu
MF.pIC.Alv.Lu
MF.microglia.CNS
MF.AT
Ep.MEChi.Th
FRC.CD140a+.Madcam-.CD35-.SLN
LEC.SLN
BEC.SLN
IAP.SLN
Expression Value Range

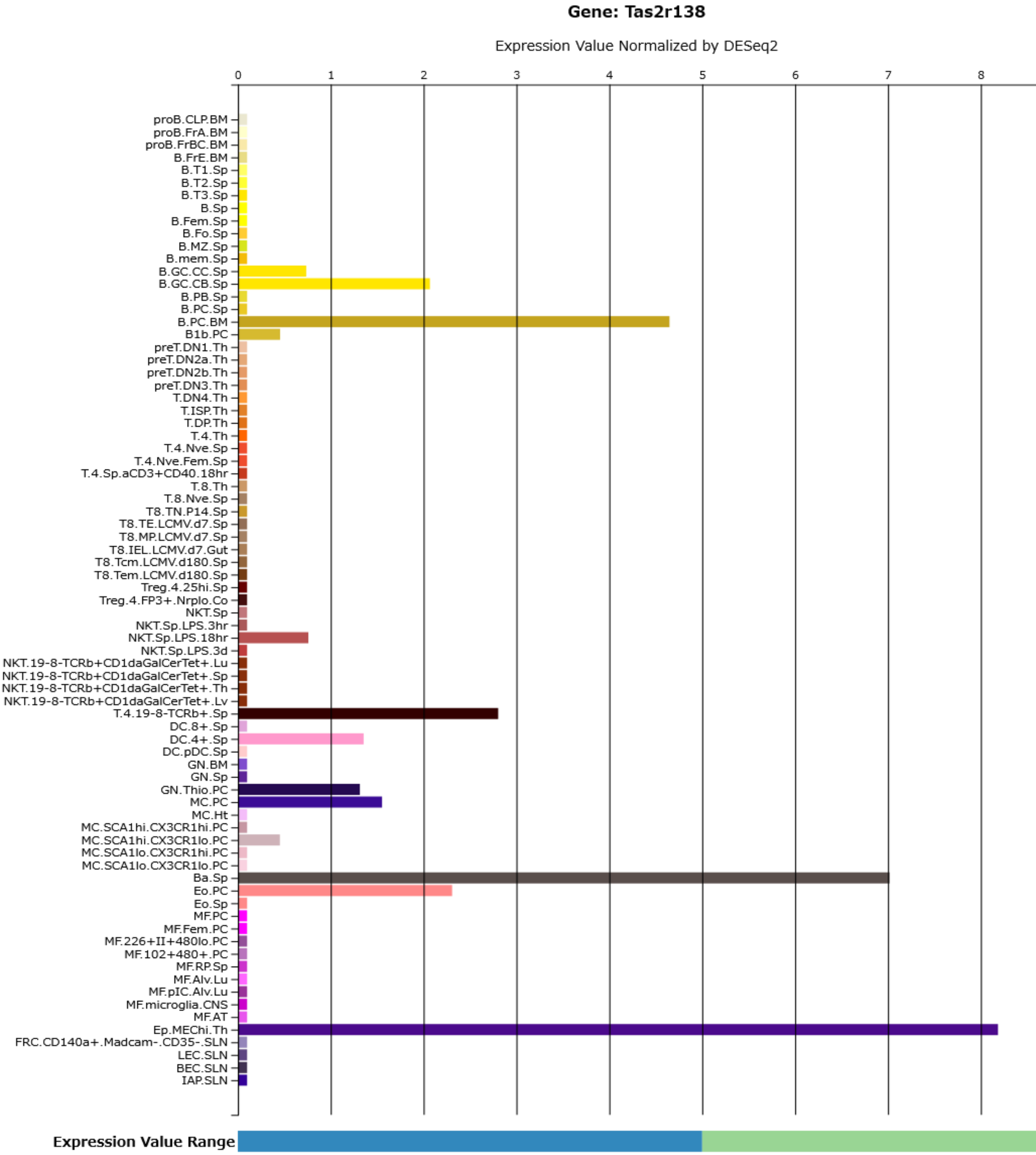

Gene: Tas2r138
Expression Value Normalized by DESeq2
0
1
2
3
4
5
6
7
8
proB.CLP.BM
proB.FrA.BM
proB.FrBC.BM
B.FrE.BM
B.T1.Sp
B.T2.Sp
B.T3.Sp
B.Sp
B.Fem.Sp
B.Fo.Sp
B.MZ.Sp
B.mem.Sp
B.GC.CC.Sp
B.GC.CB.Sp
B.PB.Sp
B.PC.Sp
B.PC.BM
B1b.PC
preT.DN1.Th
preT.DN2a.Th
preT.DN2b.Th
preT.DN3.Th
T.DN4.Th
T.ISP.Th
T.DP.Th
T.4.Th
T.4.Nve.Sp
T.4.Nve.Fem.Sp
T.4.Sp.aCD3+CD40.18hr
T.8.Th
T.8.Nve.Sp
T8.TN.P14.Sp
T8.TE.LCMV.d7.Sp
T8.MP.LCMV.d7.Sp
T8.IEL.LCMV.d7.Gut
T8.Tcm.LCMV.d180.Sp
T8.Tem.LCMV.d180.Sp
Treg.4.25hi.Sp
Treg.4.FP3+.Nrplo.Co
NKT.Sp
NKT.Sp.LPS.3hr
NKT.Sp.LPS.18hr
NKT.Sp.LPS.3d
NKT.19-8-TCRb+CD1daGalCerTet+.Lu
NKT.19-8-TCRb+CD1daGalCerTet+.Sp
NKT.19-8-TCRb+CD1daGalCerTet+.Th
NKT.19-8-TCRb+CD1daGalCerTet+.Lv
T.4.19-8-TCRb+.Sp
DC.8+.Sp
DC.4+.Sp
DC.pDC.Sp
GN.BM
GN.Sp
GN.Thio.PC
MC.PC
MC.Ht
MC.SCA1hi.CX3CR1hi.PC
MC.SCA1hi.CX3CR1lo.PC
MC.SCA1lo.CX3CR1hi.PC
MC.SCA1lo.CX3CR1lo.PC
Ba.Sp
Eo.PC
Eo.Sp
MF.PC
MF.Fem.PC
MF.226+II+480lo.PC
MF.102+480+.PC
MF.RP.Sp
MF.Alv.Lu
MF.pIC.Alv.Lu
MF.microglia.CNS
MF.AT
Ep.MEChi.Th
FRC.CD140a+.Madcam-.CD35-.SLN
LEC.SLN
BEC.SLN
IAP.SLN
Expression Value Range

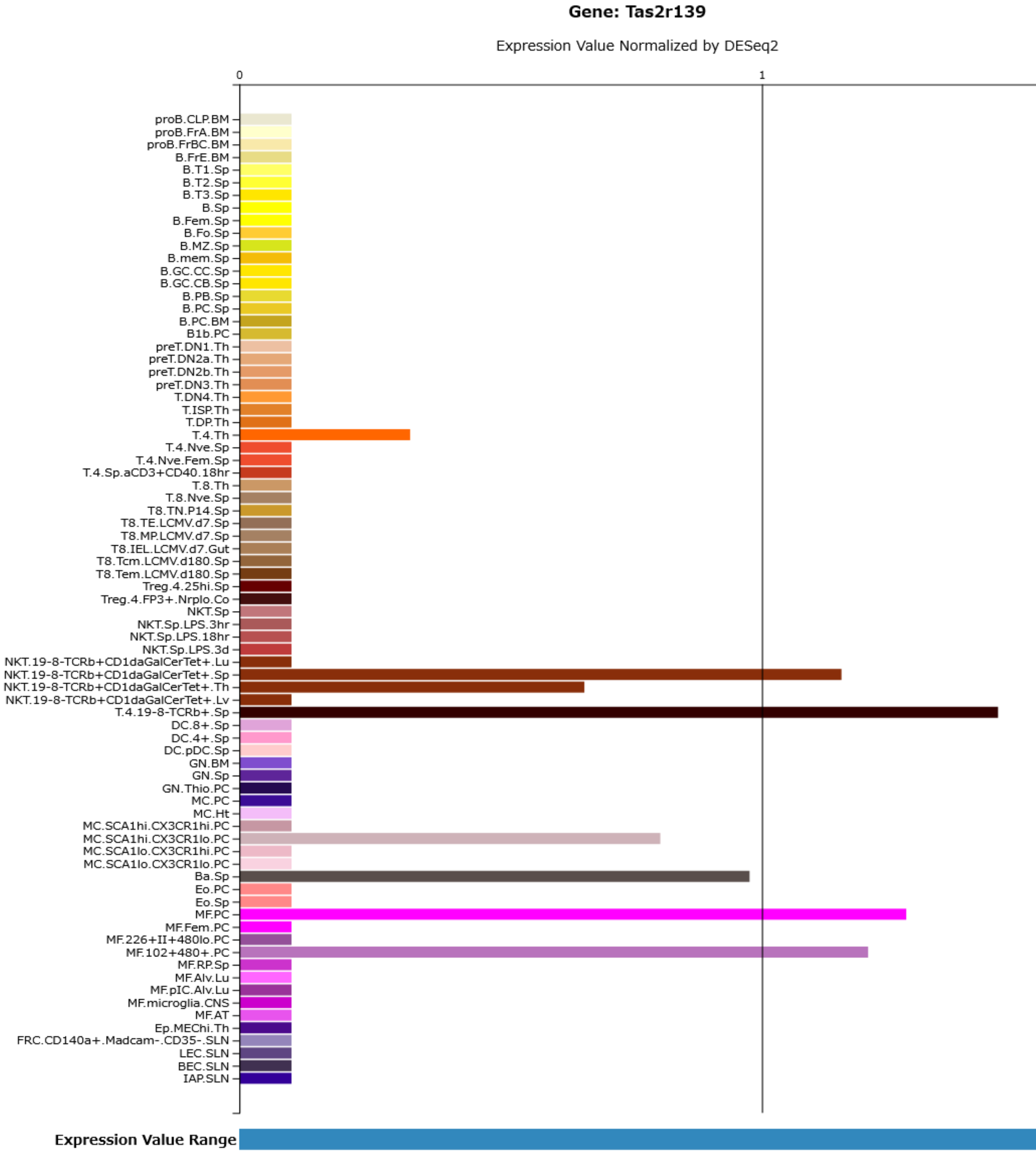
Gene: Tas2r139
Expression Value Normalized by DESeq2
0
1
proB.CLP.BM
proB.FrA.BM
proB.FrBC.BM
B.FrE.BM
B.T1.Sp
B.T2.Sp
B.T3.Sp
B.Sp
B.Fem.Sp
B.Fo.Sp
B.MZ.Sp
B.mem.Sp
B.GC.CC.Sp
B.GC.CB.Sp
B.PB.Sp
B.PC.Sp
B.PC.BM
B1b.PC
preT.DN1.Th
preT.DN2a.Th
preT.DN2b.Th
preT.DN3.Th
T.DN4.Th
T.ISP.Th
T.DP.Th
T.4.Th
T.4.Nve.Sp
T.4.Nve.Fem.Sp
T.4.Sp.aCD3+CD40.18hr
T.8.Th
T.8.Nve.Sp
T8.TN.P14.Sp
T8.TE.LCMV.d7.Sp
T8.MP.LCMV.d7.Sp
T8.IEL.LCMV.d7.Gut
T8.Tcm.LCMV.d180.Sp
T8.Tem.LCMV.d180.Sp
Treg.4.25hi.Sp
Treg.4.FP3+.Nrplo.Co
NKT.Sp
NKT.Sp.LPS.3hr
NKT.Sp.LPS.18hr
NKT.Sp.LPS.3d
NKT.19-8-TCRb+CD1daGalCerTet+.Lu
NKT.19-8-TCRb+CD1daGalCerTet+.Sp
NKT.19-8-TCRb+CD1daGalCerTet+.Th
NKT.19-8-TCRb+CD1daGalCerTet+.Lv
T.4.19-8-TCRb+.Sp
DC.8+.Sp
DC.4+.Sp
DC.pDC.Sp
GN.BM
GN.Sp
GN.Thio.PC
MC.PC
MC.Ht
MC.SCA1hi.CX3CR1hi.PC
MC.SCA1hi.CX3CR1lo.PC
MC.SCA1lo.CX3CR1hi.PC
MC.SCA1lo.CX3CR1lo.PC
Ba.Sp
Eo.PC
Eo.Sp
MF.PC
MF.Fem.PC
MF.226+II+480lo.PC
MF.102+480+.PC
MF.RP.Sp
MF.Alv.Lu
MF.pIC.Alv.Lu
MF.microglia.CNS
MF.AT
Ep.MEChi.Th
FRC.CD140a+.Madcam-.CD35-.SLN
LEC.SLN
BEC.SLN
IAP.SLN
Expression Value Range

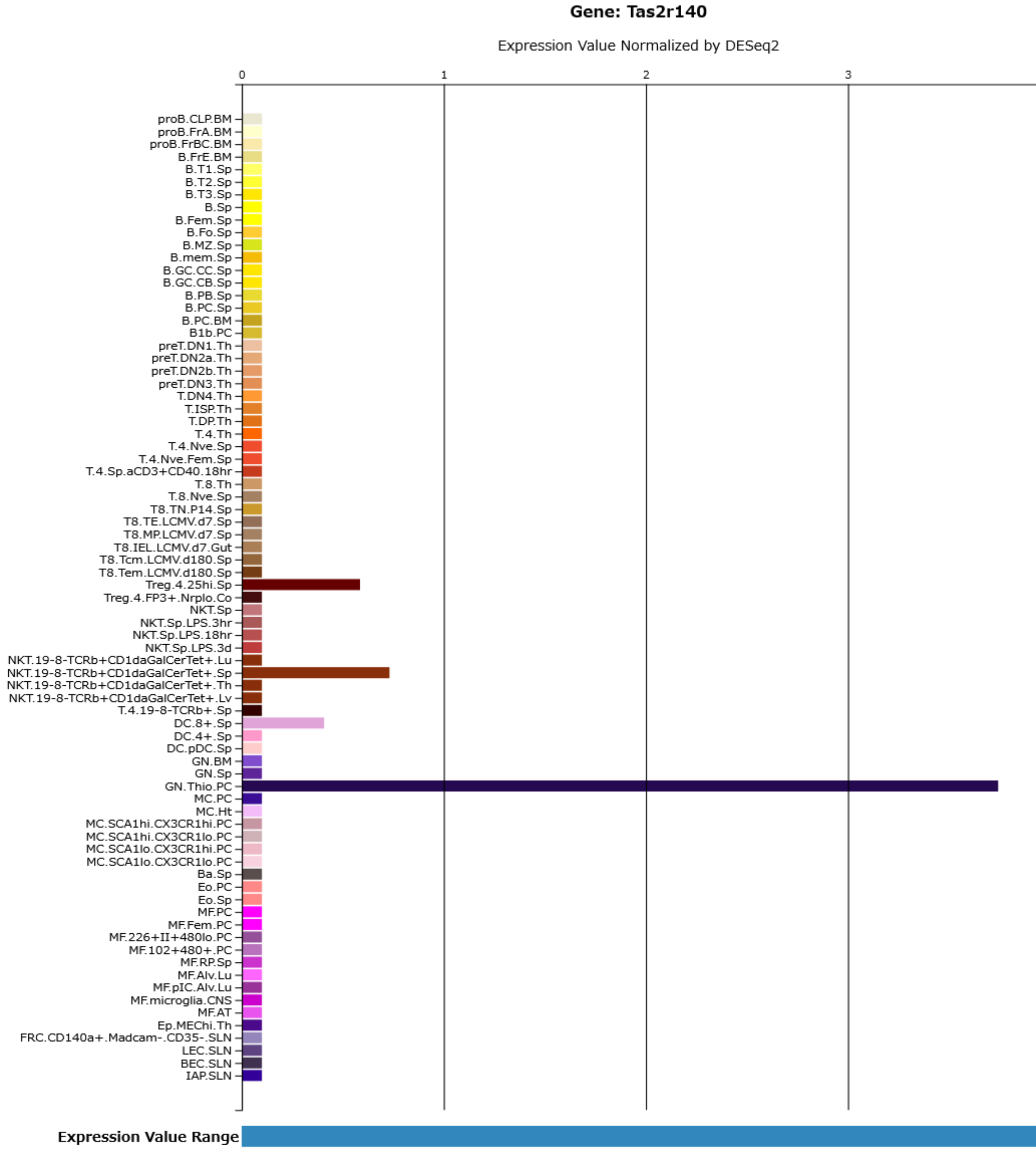
Gene: Tas2r140
Expression Value Normalized by DESeq2
0
1
2
3
proB.CLP.BM
proB.FrA.BM
proB.FrBC.BM
B.FrE.BM
B.T1.Sp
B.T2.Sp
B.T3.Sp
B.Sp
B.Fem.Sp
B.Fo.Sp
B.MZ.Sp
B.mem.Sp
B.GC.CC.Sp
B.GC.CB.Sp
B.PB.Sp
B.PC.Sp
B.PC.BM
B1b.PC
preT.DN1.Th
preT.DN2a.Th
preT.DN2b.Th
preT.DN3.Th
T.DN4.Th
T.ISP.Th
T.DP.Th
T.4.Th
T.4.Nve.Sp
T.4.Nve.Fem.Sp
T.4.Sp.aCD3+CD40.18hr
T.8.Th
T.8.Nve.Sp
T8.TN.P14.Sp
T8.TE.LCMV.d7.Sp
T8.MP.LCMV.d7.Sp
T8.IEL.LCMV.d7.Gut
T8.Tcm.LCMV.d180.Sp
T8.Tem.LCMV.d180.Sp
Treg.4.25hi.Sp
Treg.4.FP3+.Nrplo.Co
NKT.Sp
NKT.Sp.LPS.3hr
NKT.Sp.LPS.18hr
NKT.Sp.LPS.3d
NKT.19-8-TCRb+CD1daGalCerTet+.Lu
NKT.19-8-TCRb+CD1daGalCerTet+.Sp
NKT.19-8-TCRb+CD1daGalCerTet+.Th
NKT.19-8-TCRb+CD1daGalCerTet+.Lv
T.4.19-8-TCRb+.Sp
DC.8+.Sp
DC.4+.Sp
DC.pDC.Sp
GN.BM
GN.Sp
GN.Thio.PC
MC.PC
MC.Ht
MC.SCA1hi.CX3CR1hi.PC
MC.SCA1hi.CX3CR1lo.PC
MC.SCA1lo.CX3CR1hi.PC
MC.SCA1lo.CX3CR1lo.PC
Ba.Sp
Eo.PC
Eo.Sp
MF.PC
MF.Fem.PC
MF.226+II+480lo.PC
MF.102+480+.PC
MF.RP.Sp
MF.Alv.Lu
MF.pIC.Alv.Lu
MF.microglia.CNS
MF.AT
Ep.MEChi.Th
FRC.CD140a+.Madcam-.CD35-.SLN
LEC.SLN
BEC.SLN
IAP.SLN
Expression Value Range

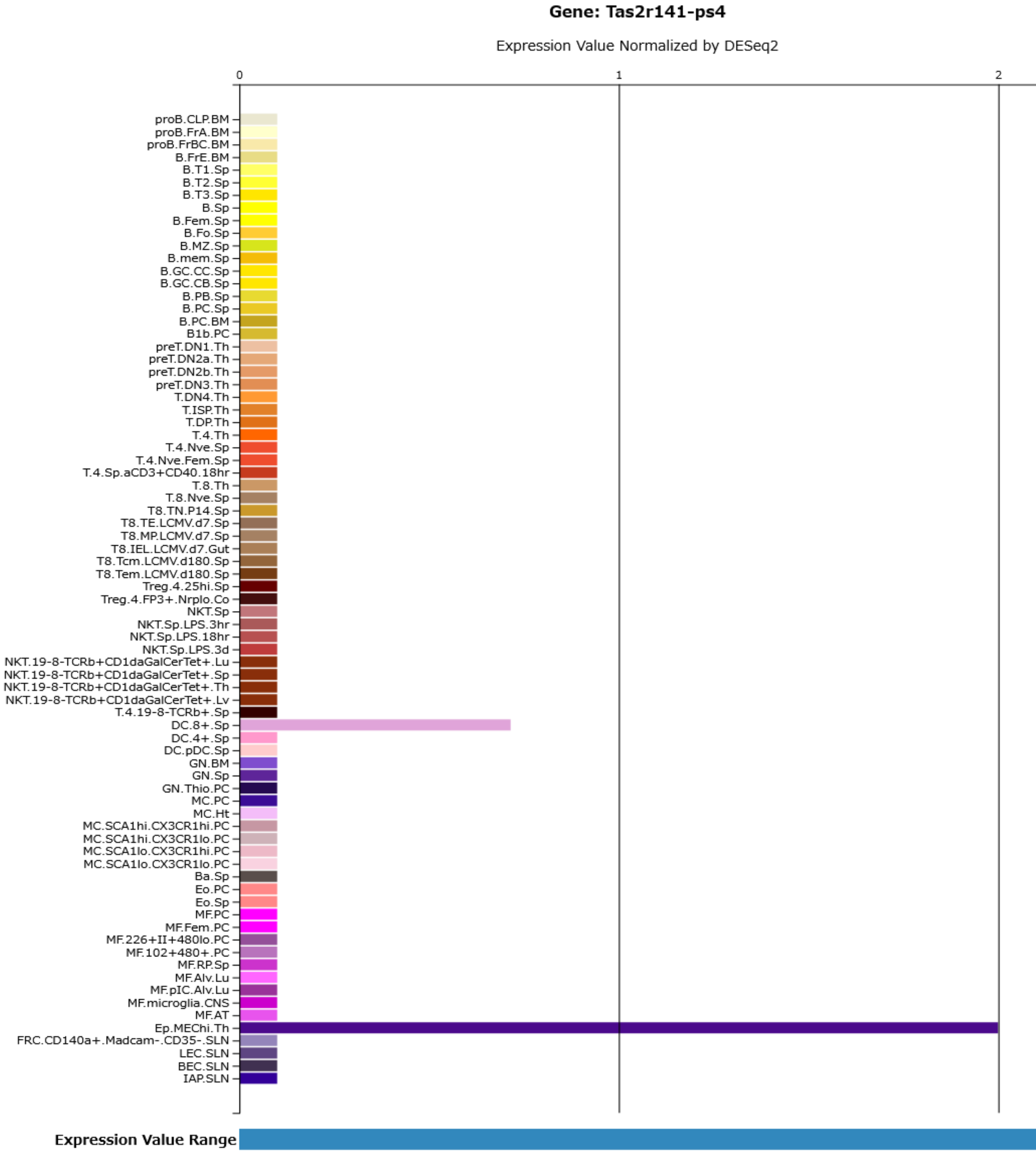

Gene: Tas2r141-ps4
Expression Value Normalized by DESeq2
0
1
2
proB.CLP.BM
proB.FrA.BM
proB.FrBC.BM
B.FrE.BM
B.T1.Sp
B.T2.Sp
B.T3.Sp
B.Sp
B.Fem.Sp
B.Fo.Sp
B.MZ.Sp
B.mem.Sp
B.GC.CC.Sp
B.GC.CB.Sp
B.PB.Sp
B.PC.Sp
B.PC.BM
B1b.PC
preT.DN1.Th
preT.DN2a.Th
preT.DN2b.Th
preT.DN3.Th
T.DN4.Th
T.ISP.Th
T.DP.Th
T.4.Th
T.4.Nve.Sp
T.4.Nve.Fem.Sp
T.4.Sp.aCD3+CD40.18hr
T.8.Th
T.8.Nve.Sp
T8.TN.P14.Sp
T8.TE.LCMV.d7.Sp
T8.MP.LCMV.d7.Sp
T8.IEL.LCMV.d7.Gut
T8.Tcm.LCMV.d180.Sp
T8.Tem.LCMV.d180.Sp
Treg.4.25hi.Sp
Treg.4.FP3+.Nrplo.Co
NKT.Sp
NKT.Sp.LPS.3hr
NKT.Sp.LPS.18hr
NKT.Sp.LPS.3d
NKT.19-8-TCRb+CD1daGalCerTet+.Lu
NKT.19-8-TCRb+CD1daGalCerTet+.Sp
NKT.19-8-TCRb+CD1daGalCerTet+.Th
NKT.19-8-TCRb+CD1daGalCerTet+.Lv
T.4.19-8-TCRb+.Sp
DC.8+.Sp
DC.4+.Sp
DC.pDC.Sp
GN.BM
GN.Sp
GN.Thio.PC
MC.PC
MC.Ht
MC.SCA1hi.CX3CR1hi.PC
MC.SCA1hi.CX3CR1lo.PC
MC.SCA1lo.CX3CR1hi.PC
MC.SCA1lo.CX3CR1lo.PC
Ba.Sp
Eo.PC
Eo.Sp
MF.PC
MF.Fem.PC
MF.226+II+480lo.PC
MF.102+480+.PC
MF.RP.Sp
MF.Alv.Lu
MF.pIC.Alv.Lu
MF.microglia.CNS
MF.AT
Ep.MEChi.Th
FRC.CD140a+.Madcam-.CD35-.SLN
LEC.SLN
BEC.SLN
IAP.SLN
Expression Value Range

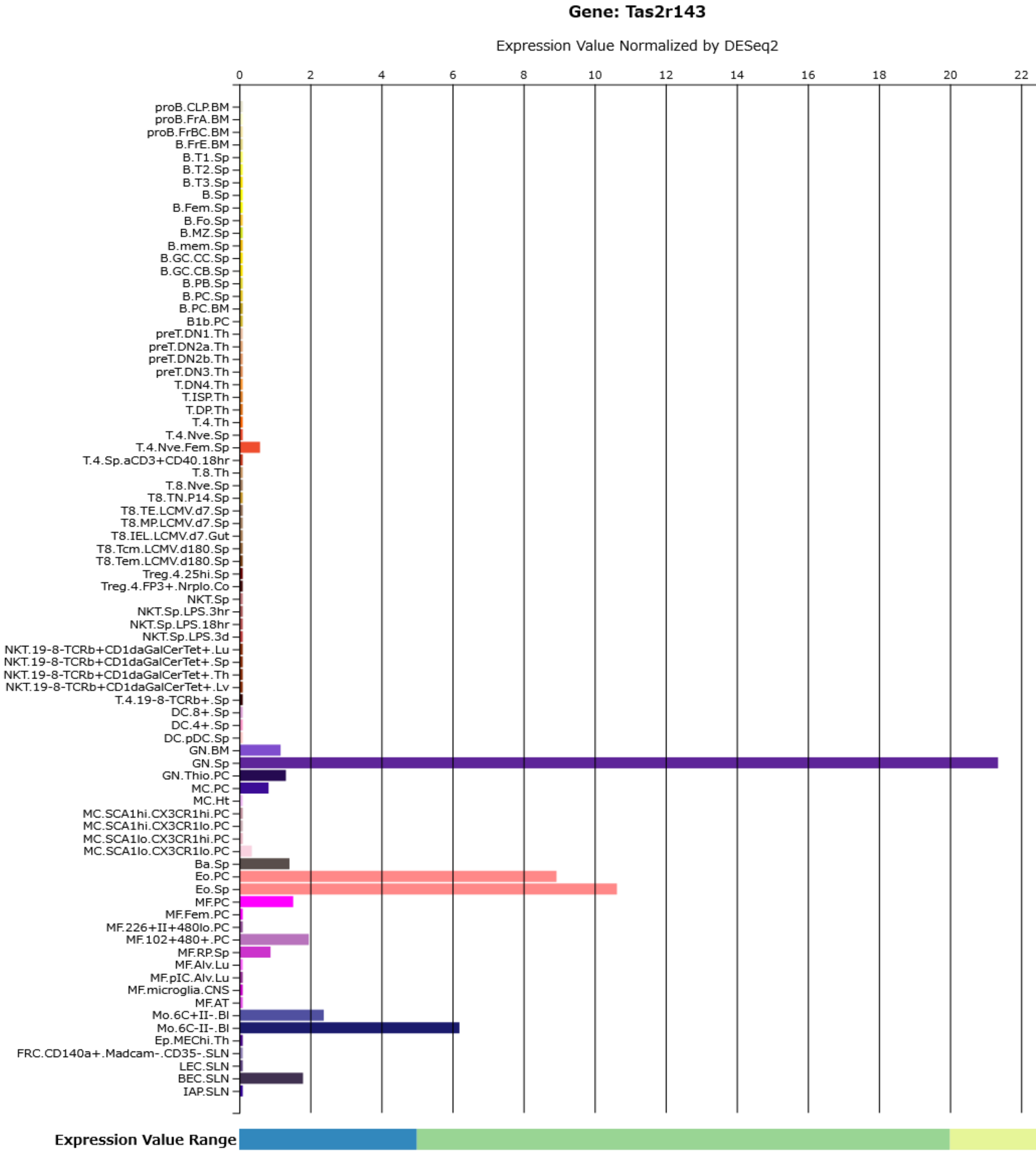
Gene: Tas2r143
Expression Value Normalized by DESeq2
0
2
4
6
8
10
12
14
16
18
20
22
proB.CLP.BM
proB.FrA.BM
proB.FrBC.BM
B.FrE.BM
B.T1.Sp
B.T2.Sp
B.T3.Sp
B.Sp
B.Fem.Sp
B.Fo.Sp
B.MZ.Sp
B.mem.Sp
B.GC.CC.Sp
B.GC.CB.Sp
B.PB.Sp
B.PC.Sp
B.PC.BM
B1b.PC
preT.DN1.Th
preT.DN2a.Th
preT.DN2b.Th
preT.DN3.Th
T.DN4.Th
T.ISP.Th
T.DP.Th
T.4.Th
T.4.Nve.Sp
T.4.Nve.Fem.Sp
T.4.Sp.aCD3+CD40.18hr
T.8.Th
T.8.Nve.Sp
T8.TN.P14.Sp
T8.TE.LCMV.d7.Sp
T8.MP.LCMV.d7.Sp
T8.IEL.LCMV.d7.Gut
T8.Tcm.LCMV.d180.Sp
T8.Tem.LCMV.d180.Sp
Treg.4.25hi.Sp
Treg.4.FP3+.Nrplo.Co
NKT.Sp
NKT.Sp.LPS.3hr
NKT.Sp.LPS.18hr
NKT.Sp.LPS.3d
NKT.19-8-TCRb+CD1daGalCerTet+.Lu
NKT.19-8-TCRb+CD1daGalCerTet+.Sp
NKT.19-8-TCRb+CD1daGalCerTet+.Th
NKT.19-8-TCRb+CD1daGalCerTet+.Lv
T.4.19-8-TCRb+.Sp
DC.8+.Sp
DC.4+.Sp
DC.pDC.Sp
GN.BM
GN.Sp
GN.Thio.PC
MC.PC
MC.Ht
MC.SCA1hi.CX3CR1hi.PC
MC.SCA1hi.CX3CR1lo.PC
MC.SCA1lo.CX3CR1hi.PC
MC.SCA1lo.CX3CR1lo.PC
Ba.Sp
Eo.PC
Eo.Sp
MF.PC
MF.Fem.PC
MF.226+II+480lo.PC
MF.102+480+.PC
MF.RP.Sp
MF.Alv.Lu
MF.pIC.Alv.Lu
MF.microglia.CNS
MF.AT
Mo.6C+II-.Bl
Mo.6C-II-.Bl
Ep.MEChi.Th
FRC.CD140a+.Madcam-.CD35-.SLN
LEC.SLN
BEC.SLN
IAP.SLN
Expression Value Range

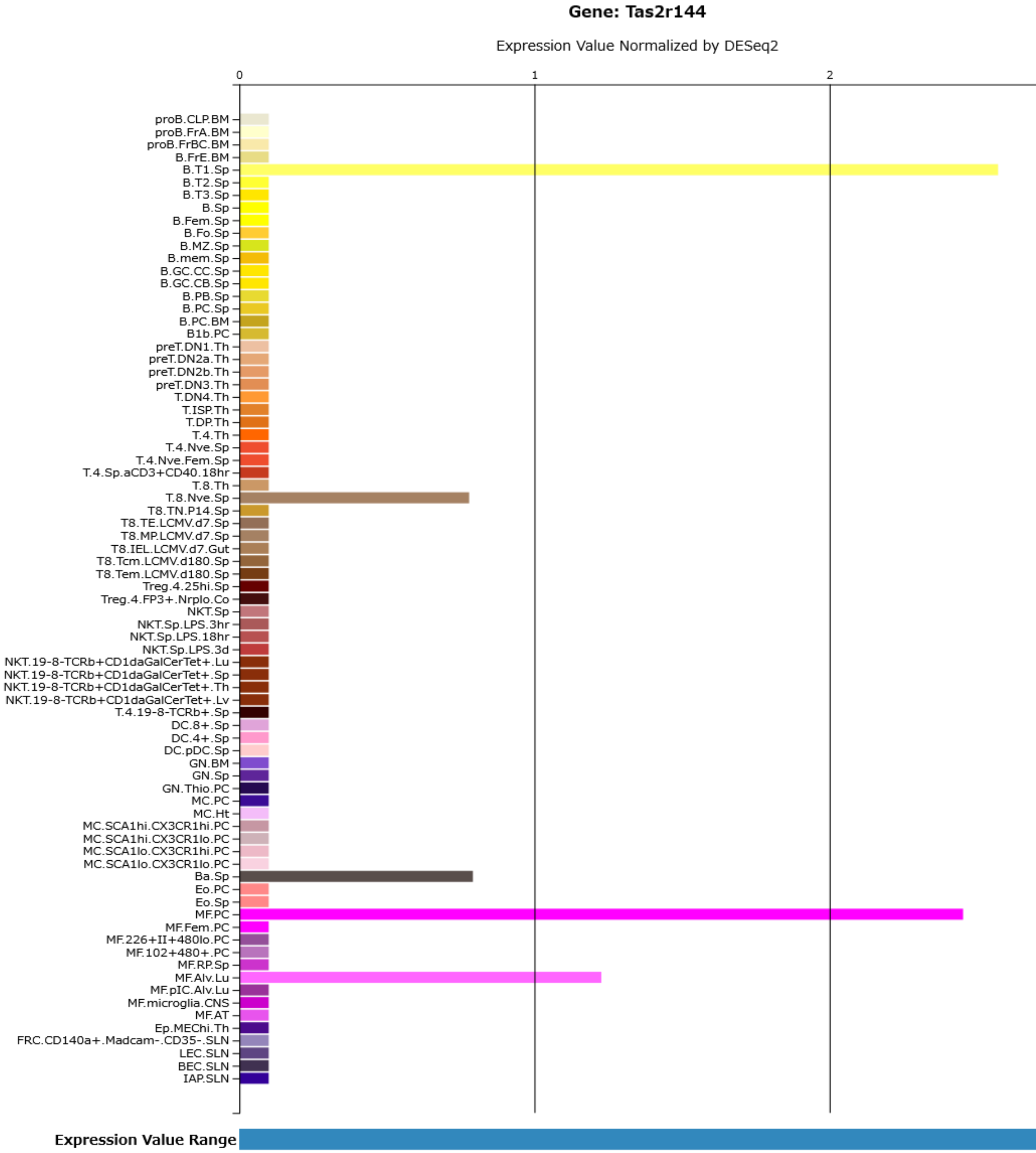
Gene: Tas2r144
Expression Value Normalized by DESeq2
0
1
2
proB.CLP.BM
proB.FrA.BM
proB.FrBC.BM
B.FrE.BM
B.T1.Sp
B.T2.Sp
B.T3.Sp
B.Sp
B.Fem.Sp
B.Fo.Sp
B.MZ.Sp
B.mem.Sp
B.GC.CC.Sp
B.GC.CB.Sp
B.PB.Sp
B.PC.Sp
B.PC.BM
B1b.PC
preT.DN1.Th
preT.DN2a.Th
preT.DN2b.Th
preT.DN3.Th
T.DN4.Th
T.ISP.Th
T.DP.Th
T.4.Th
T.4.Nve.Sp
T.4.Nve.Fem.Sp
T.4.Sp.aCD3+CD40.18hr
T.8.Th
T.8.Nve.Sp
T8.TN.P14.Sp
T8.TE.LCMV.d7.Sp
T8.MP.LCMV.d7.Sp
T8.IEL.LCMV.d7.Gut
T8.Tcm.LCMV.d180.Sp
T8.Tem.LCMV.d180.Sp
Treg.4.25hi.Sp
Treg.4.FP3+.Nrplo.Co
NKT.Sp
NKT.Sp.LPS.3hr
NKT.Sp.LPS.18hr
NKT.Sp.LPS.3d
NKT.19-8-TCRb+CD1daGalCerTet+.Lu
NKT.19-8-TCRb+CD1daGalCerTet+.Sp
NKT.19-8-TCRb+CD1daGalCerTet+.Th
NKT.19-8-TCRb+CD1daGalCerTet+.Lv
T.4.19-8-TCRb+.Sp
DC.8+.Sp
DC.4+.Sp
DC.pDC.Sp
GN.BM
GN.Sp
GN.Thio.PC
MC.PC
MC.Ht
MC.SCA1hi.CX3CR1hi.PC
MC.SCA1hi.CX3CR1lo.PC
MC.SCA1lo.CX3CR1hi.PC
MC.SCA1lo.CX3CR1lo.PC
Ba.Sp
Eo.PC
Eo.Sp
MF.PC
MF.Fem.PC
MF.226+II+480lo.PC
MF.102+480+.PC
MF.RP.Sp
MF.Alv.Lu
MF.pIC.Alv.Lu
MF.microglia.CNS
MF.AT
Ep.MEChi.Th
FRC.CD140a+.Madcam-.CD35-.SLN
LEC.SLN
BEC.SLN
IAP.SLN
Expression Value Range

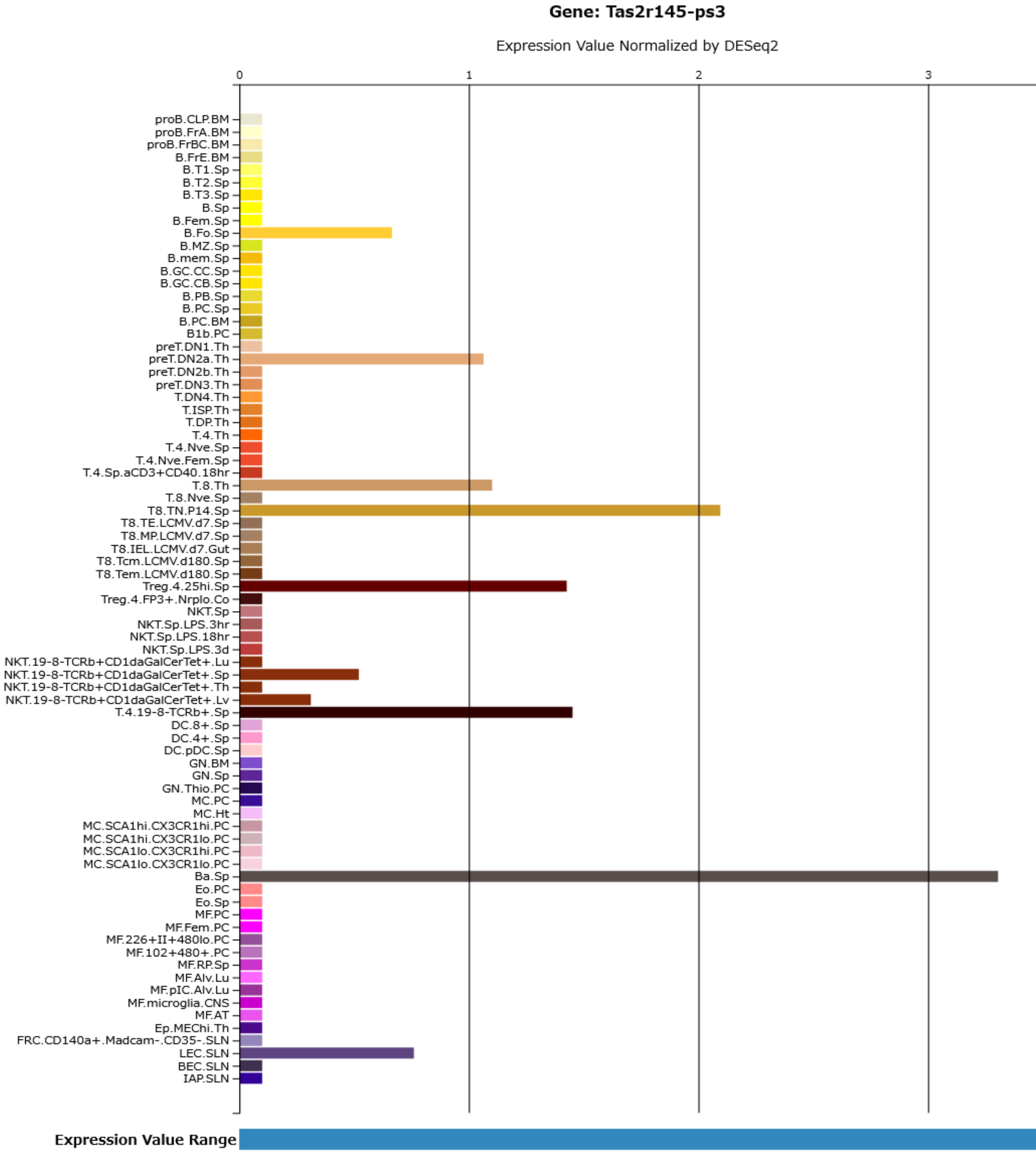
Gene: Tas2r145-ps3
Expression Value Normalized by DESeq2
0
1
2
3
proB.CLP.BM
proB.FrA.BM
proB.FrBC.BM
B.FrE.BM
B.T1.Sp
B.T2.Sp
B.T3.Sp
B.Sp
B.Fem.Sp
B.Fo.Sp
B.MZ.Sp
B.mem.Sp
B.GC.CC.Sp
B.GC.CB.Sp
B.PB.Sp
B.PC.Sp
B.PC.BM
B1b.PC
preT.DN1.Th
preT.DN2a.Th
preT.DN2b.Th
preT.DN3.Th
T.DN4.Th
T.ISP.Th
T.DP.Th
T.4.Th
T.4.Nve.Sp
T.4.Nve.Fem.Sp
T.4.Sp.aCD3+CD40.18hr
T.8.Th
T.8.Nve.Sp
T8.TN.P14.Sp
T8.TE.LCMV.d7.Sp
T8.MP.LCMV.d7.Sp
T8.IEL.LCMV.d7.Gut
T8.Tcm.LCMV.d180.Sp
T8.Tem.LCMV.d180.Sp
Treg.4.25hi.Sp
Treg.4.FP3+.Nrplo.Co
NKT.Sp
NKT.Sp.LPS.3hr
NKT.Sp.LPS.18hr
NKT.Sp.LPS.3d
NKT.19-8-TCRb+CD1daGalCerTet+.Lu
NKT.19-8-TCRb+CD1daGalCerTet+.Sp
NKT.19-8-TCRb+CD1daGalCerTet+.Th
NKT.19-8-TCRb+CD1daGalCerTet+.Lv
T.4.19-8-TCRb+.Sp
DC.8+.Sp
DC.4+.Sp
DC.pDC.Sp
GN.BM
GN.Sp
GN.Thio.PC
MC.PC
MC.Ht
MC.SCA1hi.CX3CR1hi.PC
MC.SCA1hi.CX3CR1lo.PC
MC.SCA1lo.CX3CR1hi.PC
MC.SCA1lo.CX3CR1lo.PC
Ba.Sp
Eo.PC
Eo.Sp
MF.PC
MF.Fem.PC
MF.226+II+480lo.PC
MF.102+480+.PC
MF.RP.Sp
MF.Alv.Lu
MF.pIC.Alv.Lu
MF.microglia.CNS
MF.AT
Ep.MEChi.Th
FRC.CD140a+.Madcam-.CD35-.SLN
LEC.SLN
BEC.SLN
IAP.SLN
Expression Value Range

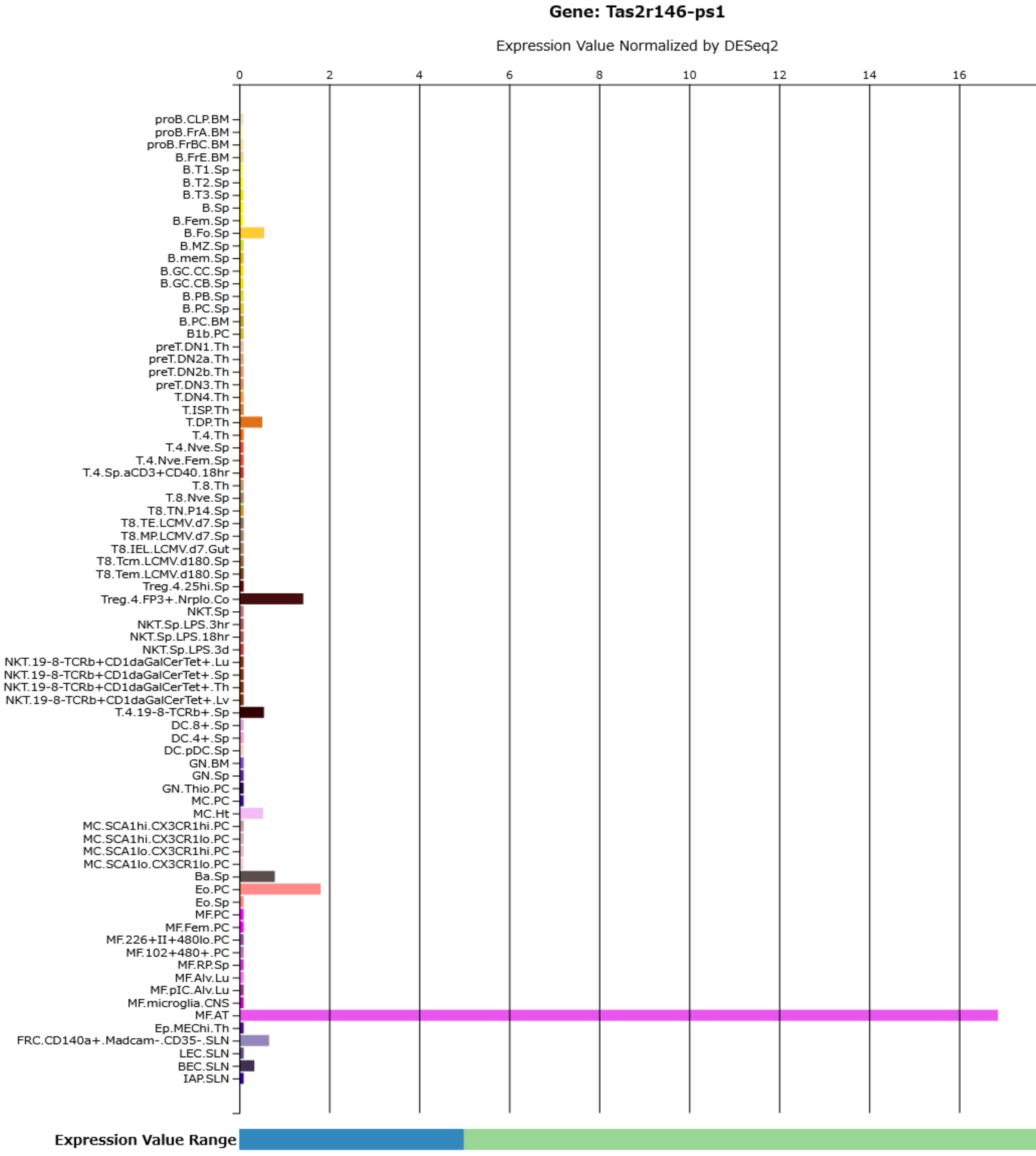
Gene: Tas2r146-ps1
Expression Value Normalized by DESeq2
0
2
4
6
8
10
12
14
16
proB.CLP.BM
proB.FrA.BM
proB.FrBC.BM
B.FrE.BM
B.T1.Sp
B.T2.Sp
B.T3.Sp
B.Sp
B.Fem.Sp
B.Fo.Sp
B.MZ.Sp
B.mem.Sp
B.GC.CC.Sp
B.GC.CB.Sp
B.PB.Sp
B.PC.Sp
B.PC.BM
B1b.PC
preT.DN1.Th
preT.DN2a.Th
preT.DN2b.Th
preT.DN3.Th
T.DN4.Th
T.ISP.Th
T.DP.Th
T.4.Th
T.4.Nve.Sp
T.4.Nve.Fem.Sp
T.4.Sp.aCD3+CD40.18hr
T.8.Th
T.8.Nve.Sp
T8.TN.P14.Sp
T8.TE.LCMV.d7.Sp
T8.MP.LCMV.d7.Sp
T8.IEL.LCMV.d7.Gut
T8.Tcm.LCMV.d180.Sp
T8.Tem.LCMV.d180.Sp
Treg.4.25hi.Sp
Treg.4.FP3+.Nrplo.Co
NKT.Sp
NKT.Sp.LPS.3hr
NKT.Sp.LPS.18hr
NKT.Sp.LPS.3d
NKT.19-8-TCRb+CD1daGalCerTet+.Lu
NKT.19-8-TCRb+CD1daGalCerTet+.Sp
NKT.19-8-TCRb+CD1daGalCerTet+.Th
NKT.19-8-TCRb+CD1daGalCerTet+.Lv
T.4.19-8-TCRb+.Sp
DC.8+.Sp
DC.4+.Sp
DC.pDC.Sp
GN.BM
GN.Sp
GN.Thio.PC
MC.PC
MC.Ht
MC.SCA1hi.CX3CR1hi.PC
MC.SCA1hi.CX3CR1lo.PC
MC.SCA1lo.CX3CR1hi.PC
MC.SCA1lo.CX3CR1lo.PC
Ba.Sp
Eo.PC
Eo.Sp
MF.PC
MF.Fem.PC
MF.226+II+480lo.PC
MF.102+480+.PC
MF.RP.Sp
MF.Alv.Lu
MF.pIC.Alv.Lu
MF.microglia.CNS
MF.AT
Ep.MEChi.Th
FRC.CD140a+.Madcam-.CD35-.SLN
LEC.SLN
BEC.SLN
IAP.SLN
Expression Value Range
